# Complex system modelling reveals oxalate homeostasis is driven by diverse oxalate-degrading bacteria

**DOI:** 10.1101/2024.10.28.620613

**Published:** 2024-10-28

**Authors:** Sromona D. Mukherjee, Carlos A. Batagello, Ava Adler, Jose Agudelo, Anna Zampini, Mangesh Suryavanshi, Andrew Nguyen, Teri Orr, Denise Dearing, Manoj Monga, Aaron W. Miller

**Affiliations:** 1 Department of Cardiovascular and Metabolic Sciences, Cleveland Clinic, Cleveland, OH, USA; 2 Division of Urology, Hospital das Clínicas, University of Sao Paulo Medical School, Sao Paulo, Brazil; 3 Department of Urology, Glickman Urological and Kidney Institute, Cleveland Clinic, Cleveland, OH, USA; 4 M Health Fairview Southdale Hospital, Edina, MN, USA; 5 Department of Biology, New Mexico State University, Las Cruces, NM, USA; 6 School of Biological Sciences, University of Utah, Salt Lake City, UT, USA; 7 Department of Urology, University of California San Diego, San Diego, CA, USA

**Keywords:** oxalate, gut microbiome, kidney stones, urinary stone disease, dietary toxins, antinutrients, complex systems, functional redundancy, homeostatic feedback, diet-mirobe-host

## Abstract

Decades of research have made clear that host-associated microbiomes touch all facets of health. However, effective therapies that target the microbiome have been elusive given its inherent complexity. Here, we experimentally examined diet-microbe-host interactions through a complex systems framework, centered on dietary oxalate. Using multiple, independent molecular, animal, and *in vitro* experimental models, we found that microbiome composition influenced multiple oxalate-microbe-host interfaces. Importantly, administration of the oxalate-degrading specialist, *Oxalobacter formigenes*, was only effective against a poor oxalate-degrading microbiota background and gives critical new insights into why clinical intervention trials with this species exhibit variable outcomes. Data suggest that, while heterogeneity in the microbiome impacts multiple diet-host-microbe interfaces, metabolic redundancy among diverse microorganisms in specific diet-microbe axes is a critical variable that may impact the efficacy of bacteriotherapies, which can help guide patient and probiotic selection criteria in probiotic clinical trials.

## Introduction

Research into the host-associated microbiome is at an inflection point. Decades of research have revealed that host-associated microbiomes are intimately linked to host health and touch all aspects of host physiology^1,2^. The first phase of microbiome research was focused on descriptive studies that characterized differences in the microbiome by host species, body site, and disease phenotypes^3–5^. Clinical case:control metagenome wide association studies that found differences in microbiome composition between healthy and disease cohorts gave rise to the ambiguous term “dysbiosis” and much of the literature is focused on “balancing” the gut microbiota^6^. In the second, and current, phase of research, studies have moved towards gaining more mechanistic insight into specific microbe-host interactions that influence host physiology and disease^6–9^. However, it is increasingly being recognized that, given the wide variability in microbiome composition, host genetics, and lifestyles, understanding mechanisms and causal relationships between gut bacteria and host physiological responses is not enough for the development of effective bacteriotherapies that target the gut microbiota^10,11^. In fact, many probiotic clinical trials, with known mechanistic links between the probiotics and host physiology, have exhibited wide variability in results^12–15^. It is currently unclear what sources of variability may drive the response to targeted bacteriotherapies. Some variables that have been proposed include heterogeneity in microbiome composition, alcohol consumption, and bowel movement quality^10^.

Complex system theory is one means to potentially constrain hypotheses and bridge the gap between mechanistic studies and effective bacteriotherapies^16,17^. Studies of the host-associated microbiome often casually acknowledge that these systems are complex and, while, there has been progress made in developing mathematical approaches to complex modelling of host-microbiome relationships^16–19^, few studies have bridged the gap between experimental biology and complex system theory. Mammals and their gut microbiome, are often considered as a holobiont^20^, defined as a discrete unit that exhibits collective action and evolves as a unit. Using evolutionary theory approaches, it is hypothesized that the form and function of the microbial part of the holobiont is driven primarily by host-microbe influences, but which require microbe-microbe interactions to help manage the enormous burden of microbiome constraint, which is termed an “ecosystem on a leash”^21^. Under this hypothesis, microbe-host interactions, in which host-associated microbes evolve to produce metabolic byproducts for the sole benefit of the host, are necessarily rare. Essential features of complex systems include functional redundancy^21,22^ and cooperation through chains of direct interactions^21–23^, distinct functional nodes that process and transfer resources^22,23^, fractality^22,23^, and adaptability through specific homeostatic feedback mechanisms to maintain relatively consistent internal conditions given a dynamic external environment^21,24^. While considerable progress has been made in understanding the nature of complex systems, there is a lack of consensus on basic terminology and broadly applicable analytical or experimental models^25^. This limitation is due in part because complex systems necessarily transect many divergent entities in nature and is thus studied in diverse scientific disciplines^25^. Thus, while some essential features of complexity have been elucidated within specific real-world systems, such as the conversion of polysaccharides to butyrate by multiple species in the gut^26^, or feedback loops in blood pressure regulation^27^, experimental evidence of multiple features of complexity within a single system is sparse, especially in microbe-host systems.

To overcome the limitations of applying complex systems theory to the microbe-host holobiont, new models and experimental frameworks are needed that balance complexity with tractability. Antinutrients, produced in plants to deter herbivory, disrupt homeostasis by targeting the function of the microbiome, host, or both^28^, and provide an effective focal point to study complexity. Oxalate is an antinutrient present in many plant-based foods, which typically provides the majority of oxalate in circulation^29^, but is also produced as a terminal metabolite in the liver^30^. While some host genetic mutations increase endogenous production of oxalate^31^, mammals do not produce enzymes capable of degrading oxalate^32^. However, multiple oxalate-degrading bacteria exist in the gut, which degrade oxalate through one of a handful of simple metabolic pathways involving one or two genes^33–35^, which isolates this function to the gut microbiota. As such, oxalate degradation as a function, exhibits a moderate amount of complexity compared to other gut microbiota functions, such as the production of trimethylamine N-oxide (TMAO), that requires host input^36^. Beyond the moderate complexity, it is known that oxalate-degrading bacteria are susceptible to antibiotics and that antibiotic use decreases oxalate degradation^37–41^. Elevated levels of oxalate induce oxidative stress, activates the inflammasome, and disrupts epithelial barrier function through tight junction proteins^42–44^. This molecule has been linked to diseases including kidney stones and chronic kidney disease^45–47^, breast cancer^48^, and cardiometabolic disorders such as atherosclerosis^49^, obesity^50^, and diabetes^50^. *Oxalobacter formigenes*, which uses oxalate as a sole carbon and energy source^33^, is a well-studied oxalate-degrading species of bacteria. The negative, at times lethal, effects of oxalate, along with the identification and mechanisms of oxalate-degrading bacteria in the gut, have been worked out for several decades^33,51–54^, pre-dating the current microbiome era. Despite this knowledge, clinical intervention trials involving *Oxalobacter formigenes*, which is perhaps the most effective oxalate-degrading species known, have successfully resulted in a significant reduction in urine oxalate levels in only 43% of studies^15,55^. Oxalate-degrading lactic acid bacteria have been successful in 37.5% of studies^15^. Both treatments led to a wide variability in patient responses. Given these data and the well worked out mechanisms of oxalate metabolism, it is clear that having an understanding of the mechanistic links between the gut microbiota and host physiology, alone, is demonstrably not sufficient to develop effective bacteriotherapies. Therefore, even though the history of oxalate-microbe-host interactions is much greater than most other microbe-host systems, oxalate degradation represents an accurate reflection of the challenges faced by microbiome research and is a prime candidate for complex system modelling to understand the critical variables that determine responsiveness to bacteriotherapies.

The objectives of the current study were to evaluate oxalate-microbe-host interactions, within the framework of complex systems. We used multi-omic approaches utilizing multiple independent *in vivo* and *in vitro* models to understand the critical variables that influence the gut microbiota’s maintenance of oxalate homeostasis and its impact on the host. We targeted several potential oxalate-microbe-host interfaces that include the gut microbiota itself, which is one of the first lines of defense against antinutrients^56,57^, intestinal epithelium, which is an important barrier between microbe and host^21^, the liver, which is important for the biotransformation of dietary or microbial metabolites^58^, along with the kidneys and vasculature, where calcium oxalate can potentially form calcified deposits or induce inflammation^49,59–61^. Collectively, data indicate that multiple oxalate-microbe-host interfaces are influenced significantly by gut microbiota composition and that harboring diverse microorganisms capable of degrading oxalate can limit the impact of *O. formigenes* as a probiotic. Based on results from this study, we propose a phased approach for the development of bacteriotherapies whereby clinical case:control studies determine whether or not a clinical phenotype is associated with the microbiome and the microbial taxa/functions associated with specific phenotypes (Phase I). Hypothesized taxa and/or microbial functions should be mechanistically resolved through *in vitro* and germ-free animal studies (Phase II). Finally, mechanistic insights should be applied to a complex systems theoretical framework to identify those variables that most impact the potential success of bacteriotherapies (Phase III). Such a phased approach would have broad implications for patient and probiotic selection in the development of targeted bacteriotherapies.

## Results

### Defining the oxalate-microbe-host as a complex system

To use complex system modelling as a means to identify the most pertinent variables that impact oxalate-microbe-host interactions, we first had to define the system as a series of nodes, whereby oxalate is processed, connections, where the end-product of one node is transferred to another node, and fractals, which are autonomous subunits or layers that exhibit analogous, but unique, functions to other layers. For antinutrients, such as oxalate, we can define four unique nodes where these molecules can either be transformed or where they can transform the host. The first node is the stomach where the low pH can biotransform some molecules, such as glucosinolates^62^. Some proportion of the molecules can be absorbed into the circulation in the stomach. Stomach contents are then transferred to the intestines, the second node, where they are first exposed to high densities of gut bacteria, particularly in the colon. Within the gut microbiota, there are two fractal layers that include microbial species and the genes within each species. In each of these fractal layers, there are three hypotheses that can be generated about how microbes can degrade oxalate or other antinutrients. These include a) one species/gene, one function; b) multiple species/genes, one function; or c) multiple species, multiple functions. Canonically, the hypothesis is that one single species, *O. formigenes*, is primarily responsible for oxalate degradation^63^, which is achieved through a two gene metabolic pathway (oxalyl-CoA decarboxylase and formyl-CoA transferase)^53,54^. However, more recent studies show that many species of gut bacteria degrade oxalate and multiple metabolic pathways can be used to degrade oxalate that comprise of one or two genes^34,35,64,65^. Furthermore, studies have shown that there are broad interactions between the gut microbiota and oxalate degradation^66–72^. Finally, two of the most common by-products of oxalate degradation are CO_2_ and formate. Importantly, these by-products can be used in downstream metabolic pathways such as acetogenesis, methanogenesis, and sulfate-reduction^73–76^. Given the inconsistent results in *O. formigenes* trials and the recognition that there are broad gut microbiota-oxalate interactions that maintain oxalate homeostasis, we can hypothesize that, instead of relying on a single species, oxalate homeostasis is maintained either by diverse oxalate-degrading bacteria or by cooperative networks whereby a small number of oxalate-degrading bacteria produce substrates to be used in downstream metabolic pathways (i.e. multiple species, one function or multiple species, multiple functions). Again, some proportion of oxalate in the intestines gets absorbed into circulation or is excreted in the stool. However, oxalate can also be secreted back into the gut through transporters such as SLC26a6^77,78^. Another proportion of the molecule can be transported through the portal vein to the liver for hepatic biotransformation^79^. Importantly, for oxalate, the liver is a source, rather than a sink^32^. However, for other molecules, such as trimethylamine that is produced by gut bacteria, the liver is critical for converting the molecule to the more pathogenic TMAO form^36^. Some complex antinutrients, such as creosote, require both the gut microbiota and the liver^80^. The liver can also be divided into fractal layers based on cell type and genes. Oxalate or other antinutrients that are neither excreted in the stool, nor degraded by the gut microbiota, nor degraded by the liver, can impact one or more organs and interact with the host through localized immune responses, with the remaining being excreted in the urine. As such, this model sets up multiple testable hypotheses, which are explored in depth, below.

### Constitutive and oxalate-dependent effects of the microbiome impact host hepatic gene expression

**Fig. 1.**
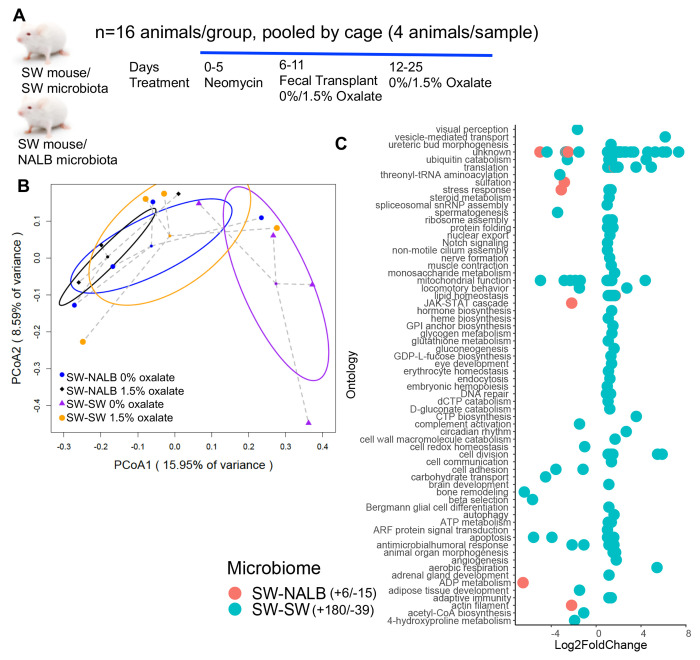
Oxalate exposure impacted host hepatic activity in a microbiota dependent fashion. A) Swiss Webster mice were given neomycin, followed by an allograft (SWM) or xenograft (NALB) fecal transplant, then maintained on a 0% or 1.5% oxalate diet prior to necropsy for fecal metabolomics (Fig. 2) and hepatic transcriptomics. B) PCoA of normalized, whole-transcriptome data. p=0.02 for microbiome composition, p=0.07 for dietary oxalate content, p=0.3 in 2-way analysis; 2-way PERMANOVA. C) Total number of hepatic genes significantly stimulated or inhibited by dietary oxalate. Significant genes are plotted by Log2FoldChange. Positive values reflect genes increased with oxalate exposure and negative values are genes decreased with exposure. FDR < 0.05, Wald test. Hepatic genes are annotated to pathway (Kegg, Uniprot, PubChem, Metacyc) and the total number of genes that exhibit a positive or negative shift with oxalate exposure are listed in the legend. Complete gene list is in Table S2.

Both the gut microbiota and the liver are important organs for the neutralization of antinutrients^58,81^, indicative of functional redundancy, cooperation, and fractality. While it is known that the liver does not degrade oxalate itself, it could still be impacted by oxalate, in gut microbiota dependent ways. To determine the effects of oxalate exposure and microbiome on host hepatic gene expression, we used a fecal transplant model to examine different host microbiomes with the same host genetics. Specifically, a five-day course of neomycin was used to suppress the native gut microbiota of Swiss-Webster mice (SWM)^38^, followed by fecal transplants either from SWM (allograft; SW-SW) or *N. albigulia* (NALB), which has a highly effective and transferable oxalate-degrading gut microbiota^68,69,72^, (xenograft; SW-NALB). Subsequently, animals were fed either a 0% or 1.5% oxalate diet (Evigo, Fig. 1A, Table S1). Liver tissue was obtained after three weeks and processed for bulk RNAseq. The RNAseq data analyses revealed constitutive microbiome and microbiome-dependent oxalate effects (Fig. 1B,C), whereby SW-SW mice exhibited an oxalate-dependent alteration of 219 hepatic genes, with a net increase in activity, while the SW-NALB mice exhibited an oxalate-dependent alteration of 21 genes with a net decrease in activity (Fig. 1C, Table S2). In the SW-NALB mice, the primary response was a decrease in sulfation activity with oxalate exposure, which is involved in the deactivation, detoxication, and excretion of xenobiotics (Fig 1C), and suggests oxalate may be beneficial for this host-microbe system^82^. The primary response in the SW-SW mice was an increase in mitochondrial activity, translation, protein regulation, and ribosome biogenesis, indicative of oxalate-induced hepatic stress^49,83^. Since hosts only differed by gut microbiota composition, these data demonstrate causative interactions between the gut microbiota and liver activity through oxalate-dependent and independent pathways. Within the framework of complex systems, results show microbe-host cooperation whereby oxalate effectively processed within the SW-NALB gut microbiota reduced overall liver activity, indicative of a beneficial impact. Data also suggest that both the gut microbiota and the immune system are involved in oxalate remediation (redundancy), such that if oxalate cannot be neutralized in the gut microbiota or liver, then the molecule will be processed through host immune response mechanisms (fractality), in this case indicated through an overall increase in hepatic activity and specifically in mitochondrial activity.

### Constitutive and oxalate-dependent effects of the microbiome impact microbial metabolic activity in the gut

**Fig. 2.**
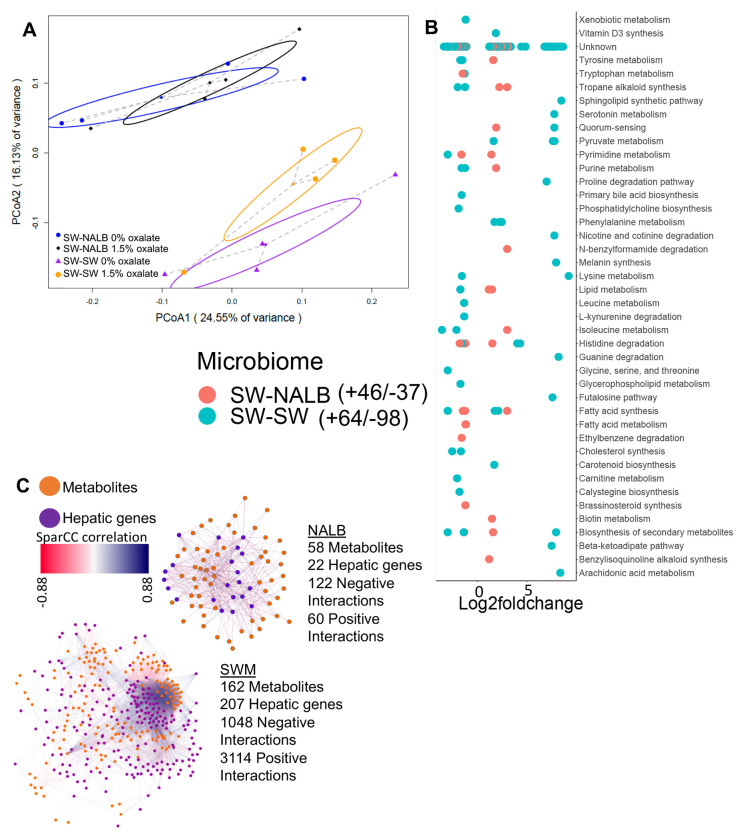
Oxalate exposure impacted microbial metabolic activity in a microbiota dependent fashion. A) PCoA of protein normalized, log-transformed metabolomic data. p=0.001 for microbiome composition, p=0.1 for dietary oxalate content, p=0.3 in 2-way analysis; 2-way PERMANOVA. B) Total number of fecal metabolites significantly stimulated or inhibited by dietary oxalate. Significant metabolites are plotted by Log2FoldChange. Positive values reflect metabolites increased with oxalate exposure and negative values are metabolites decreased with exposure. FDR < 0.05, Mann-Whitney U or Fisher’s exact test. Metabolites are annotated to pathway (Kegg, Uniprot, PubChem, Metacyc) the total number of metabolites that exhibit a positive or negative shift with oxalate exposure are listed in the legend. Complete list is in Table S3. C) Change in the host-microbe interaction network upon exposure to oxalate, quantified as hepatic gene-microbe metabolite correlations R > +/− 0.3 and FDR < 0.05 with SparCC, visualized in Cytoscape. All host-microbe interactions are listed in Table S4.

Microbe-microbe interactions are important features of the microbe-host holobiont within the conext of complex systems, both in terms of constraining the microbiome and in processing dietary components^21,22,26,56^. To assess changes to microbial metabolic output with oxalate exposure, we used the same animals as above. Following the diet trial, colon stool was collected post-necropsy and processed for untargeted metabolomics, which is a measure of total microbial metabolic output. Collectively, results are indicative of constitutive microbiome and microbiome-dependent oxalate effects (Fig. 2A,B). The SW-SW mice exhibited an alteration of 162 microbial metabolites upon oxalate exposure, with a net decrease in activity compared to the no oxalate group, whereas the SW-NALB mice exhibited an alteration of 83 microbial metabolites, with a net increase in activity compared to the no oxalate group (Fig. 2B, Table S3). In SW-NALB mice, the primary response was an increase in lipid metabolism and a shift (increase/decrease) in fatty acid, secondary metabolite, and alkaloid profiles. In the SW-SW mice, the primary response was an increase in fatty acid synthesis and phenylalanine metabolism, and a decrease in synthesis of secondary metabolites, cholesterol, and alkaloids (Fig. 2B). Therefore, while oxalate had a much greater impact on gut microbial metabolism in SW-SW mice overall, the metabolic pathways impacted were similar. Net changes in microbial metabolites produced are indicative of a negative impact of oxalate on microbial activity in SW-SW and positive impact in SW-NALB mice. Integration of host hepatic gene expression and gut metabolomic data shows that oxalate induces a small decrease in hepatic activity overall for SW-NALB mice and a large increase in hepatic activity for SW-SW mice (Fig. 2C, Table S4). From a complex systems perspective, data reflect a causative effect of oxalate for the shift in microbial metabolic output. Specifically, the SW-NALB mice exhibit hallmarks of homeostatic feedback with oxalate exposure to maintain a consistent metabolic output, defined by the relatively small, net negative, microbial metabolite-hepatic gene network compared to the large, net positive, network of SW-SW mice. Additionally, data further support the cooperation, redundancy, and fractality of the gut-liver axis. While SW-NALB exhibit a small increase in microbial metabolic activity and decrease in liver activity, the SW-SW mice saw a large decrease in microbial metabolic activity, coupled with a large increase in hepatic activity, which is reflected in the multi-omic network profiles (Fig. 2C).

### Oxalate stimulates the growth of microbial populations involved in oxalate metabolism, formate metabolism, and their precursors

**Fig. 3.**
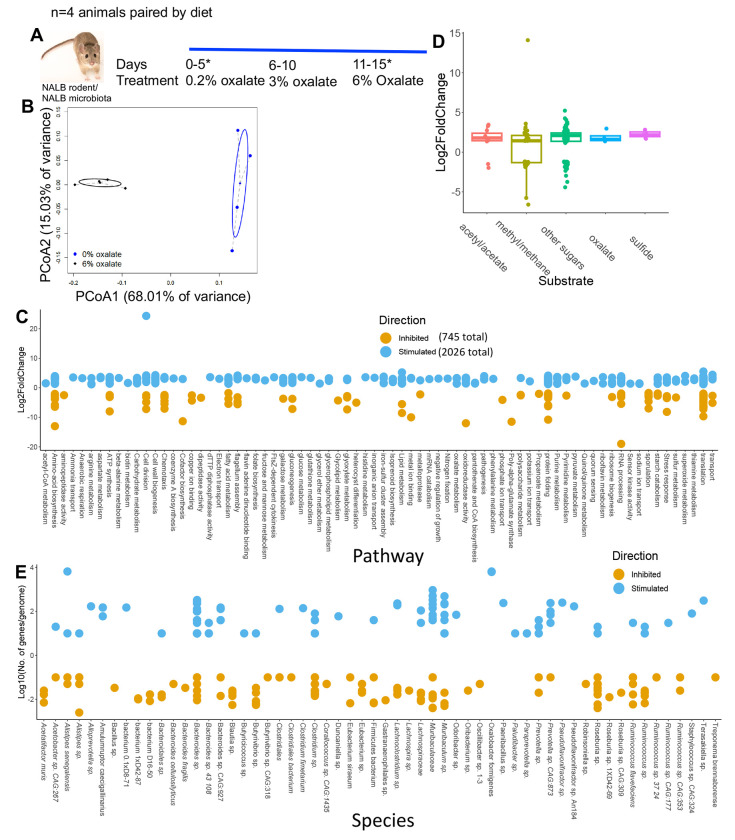
Oxalate exposure stimulates taxonomically diverse microorganisms, with a few strains that dominate the response. A) *Neotoma albigula* with native microbiota were given increasing amounts of dietary oxalate up to 6% w/w. *indicates sampling timepoints. B) PCoA of normalized metagenomic data. p=0.02; PERMANOVA. C) Total number of microbial genes significantly stimulated or inhibited by dietary oxalate, annotated to pathway (Kegg, UniProt, PubChem, Metacyc) and listed by Log2FoldChange. The total number of genes stimulated or inhibited by oxalate are listed in the legend. Genes with unknown annotation are not listed. The complete list of annotated genes is listed in Table S5. D) Number of significantly differentiated genes involved in oxalate degradation, sulfate reduction, acetogenic, methanogenic, or sugar metabolic pathways or utilization of by-products of those pathways stimulated (positive) or inhibited (negative) by dietary oxalate. Genes are listed by their Log2FoldChange between no and high oxalate diets. FDR < 0.05, Wald test. E) The number of genes/genome significantly altered by oxalate, mapped to microbial genomes extracted from *N. albigula*. Number of genes/genomes are log10-transformed to show the distribution more clearly. A total of 92% of genomes had at least one significantly altered gene population mapped to them.

To gain a deeper understanding of microbe-microbe interactions associated with oxalate exposure and to identify specific microorganisms that positively respond to oxalate exposure, NALB with their native microbiota were pair-fed increasing amounts of oxalate, from 0.2% to 6% (Fig. 3A). Stool was sampled after 5 days on the 0.2% and 6% diets and processed for shotgun metagenomics. Analysis of metagenomic data revealed that oxalate had a significant impact on metagenome composition (Fig. 3B), with a significant increase of 1073 gene populations and decrease of 382 gene populations (Fig. 3C; Table S5). Changes in gene abundance included four oxalate metabolism gene populations, a shift in 48 gene populations involved in formate metabolism (32 +/16 −), and a shift in genes related to glycine and glyoxylate/dicarboxylate metabolism (6+/4−), which are precursors to oxalate (Fig. 3D). A total of 128 differentially abundant genes were involved in sugar metabolism. Altered gene populations primarily belonged to *O. formigenes* and *Alistipes senegalensis*, with many genes belonging to the *Muribaculum* genus (Fig. 3E). *Muribaculum spp* harbor oxalate-degrading genes, which suggests oxalate metabolic redundancy in the gut microbiota^84^. The oxalate-dependent metagenomic divergence of the NALB gut microbiota (Fig. 3), combined with the lack of change in the microbial metabolomic profile with oxalate exposure (Fig. 2), suggest that oxalate stimulates taxonomically diverse, but metabolically redundant microorganisms, in support of maintaining homeostasis. Given that data came from the same hosts sampled longitudinally, these data also reflect a microbiota that is adaptive to oxalate exposure, which is another important characteristic of complex systems.

### Oxalate and formate metabolism are highly redundant functions in the NALB gut

**Fig. 4.**
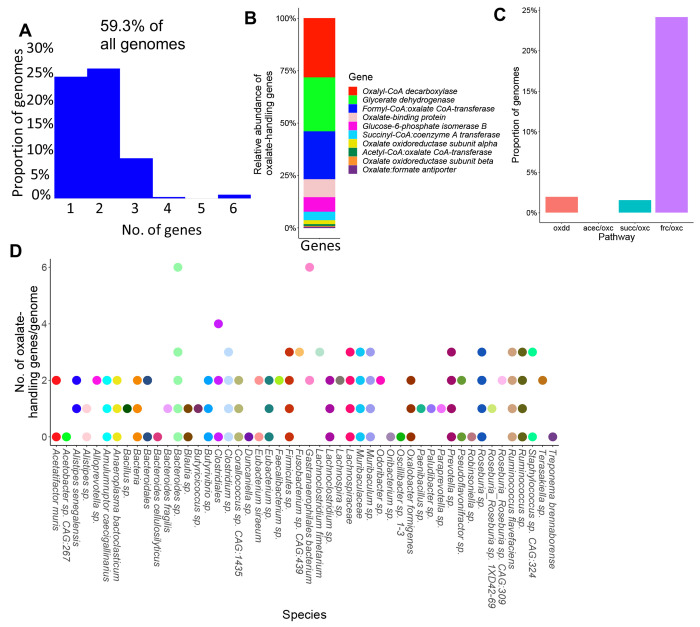
Genes related to the metabolism or handling of oxalate are present in >50% of 248 full length NALB microbial genomes from the gut. A) Proportion of the genomes extracted from *N. albigula* that had at least one oxalate-related gene. B) Relative distribution of oxalate-handling genes by gene function. C) Proportion of genomes that have a complete pathway for oxalate degradation, specifically. oxdd=oxalate oxidoreductase; acec=acetyl-CoA:oxalate CoA-transferase; oxc=Oxalyl-CoA decarboxylase; succ=succinyl-CoA:coenzyme A transferase; frc=Formyl-CoA transferase; D) Number of oxalate-handling genes by genome.

To investigate the hypothesis that oxalate stimulates a taxonomically diverse, metabolically redundant community, 248 full length genomes were extracted from shotgun metagenomic data (Fig. S1). Genes for oxalate metabolism, formate metabolism, or the formate metabolic pathways of acetogenesis, methanogenesis, and sulfate reduction, were derived from the KEGG pathway database^85^ and mapped to full length genomes. This analysis provides a targeted assessment for metabolic redundancy aimed at oxalate metabolism and pathways associated with the by-products of oxalate metabolism (potential cooperation). A total of 59.3% of genomes contained at least one gene associated with oxalate metabolism or handling (Fig. 4A), most represented by oxalyl-CoA decarboxylase, glycerate dehydrogenase, and formyl-CoA:oxalate CoA transferase (Fig. 4B). However, only 27.8% of genomes harbored a complete metabolic pathway for oxalate degradation (Fig. 4C). Taxa with oxalate genes were dominated by *Bacteroides, Muribaculaceae, Clostridium, Ruminococcus*, and *Lachnospiraceae* (Fig. 4D). Formate metabolism genes were found in 97.18% of genomes, which was dominated by serine hydroxymethyltransferase, and formate-tetrahydrofolate ligase (Fig. S2A–C). Acetogenic genes were also present in 97.18% of genomes, dominated by acetate kinase and formate-tetrahydrofolate ligase (Fig. S3A–C). Methanogenic genes were present in 100% of genomes, dominated by phosphoserine phosphatase, atp-dependent 6-phosphofructokinase, and phosphate acetyltransferase (Fig. S4A–C). Sulfate-reducing genes were present in 31.05% of genomes, dominated by bifunctional oligoribonuclease and PAP phosphatase, FMN reductase, and cysteine synthase (Fig. S5A–C). Data show highly redundant oxalate-associated metabolic pathways and thus provide evidence for very robust homeostatic feedback mechanisms to handle oxalate and metabolic by-products within the NALB gut microbiota. Additionally, the broad diversity of species that contain oxalate-related genes suggest that the distribution of metabolic genes is somewhat independent of the distribution of microbial species, which suggests that microbial genes exist in an autonomous fractal layer, to some degree. This hypothesis is supported by studies which show a high degree of horizontal gene transfer within the gut microbiota as a means of adaptation^86^.

### Oxalate metabolism is driven both by substrate availability and microbiota composition

**Fig. 5.**
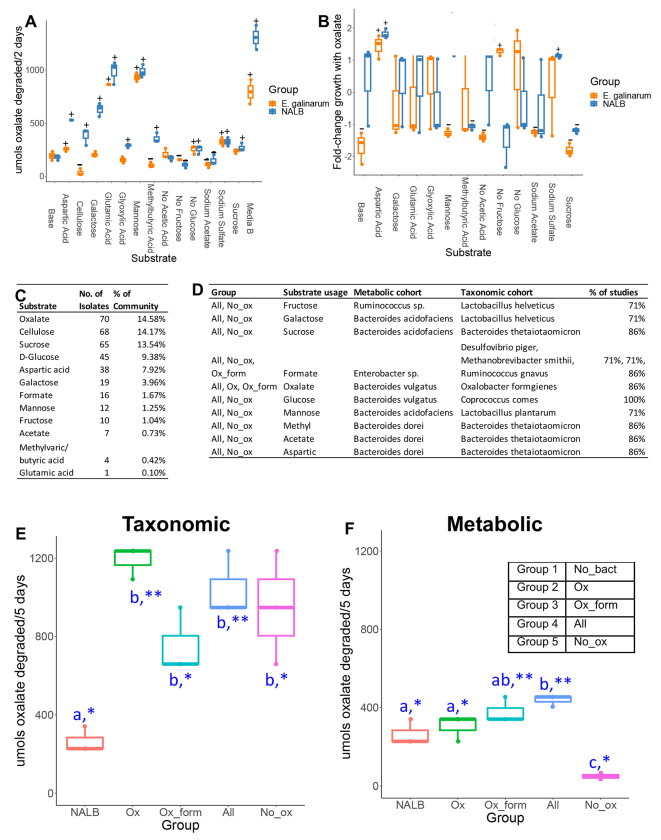
Microbial community composition and available substrates impact oxalate metabolism and the impact of oxalate on growth. A) Substrates associated with metabolic pathways enriched by exposure to dietary oxalate *in vivo* differentially impact oxalate metabolism. p<0.001 in one-way and two-way ANOVA against bacterial group and substrate; +/− reflects p<0.05, Holm’s-corrected, pairwise t-test compared to base media for an increase (+) or decrease (−) in oxalate degradation. B) Substrates differentially impact the influence of oxalate on microbial growth; p<0.001 in two-way ANOVA against substrate and bacterial group, and one-way analysis against substrate; +/− reflects p<0.05, Holm’s-corrected, one-sample t-test compared 0 (no impact of oxalate) for an increase (+) or decrease (−) in growth due to oxalate exposure. The impact of oxalate on growth was not calculated for cellulose or Media B. C) Culture-based means to quantify proportion of the NALB community that can use substrates identified through shotgun metagenomics as sole carbon and energy sources. D) Defined microbial communities to assess oxalate metabolism *in vitro* and *in vivo*. Listed are the microbial consortia, which substrate the microbes utilize that corresponds to the shotgun metagenomic data, taxonomic classification of microorganisms used in the two cohorts, and the proportion of studies in which microorganisms in the taxonomic cohort were stimulated by oxalate exposure. E,F) Oxalate metabolism in minimal media with 20mM oxalate from the microbial communities listed in 5D, in comparison to the NALB community. p<0.001, ANOVA comparing microbial groups. *p<0.05, **p<0.01; ***p<0.001; Holm’s corrected, one sample t-tests against 0 (no oxalate metabolism). Blue letters reflect statistical groups between microbial groups for oxalate metabolism.

Adaptability and homeostatic feedback within complex systems is driven by the convergence of system components and resource availability^24^. To examine the confluence of resource availability and oxalate metabolism, a custom medium based on previously published gut microbiota media^87^ was modified by adding substrates associated with metabolic pathways enriched by oxalate (Fig. 3C, Table S6). The oxalate-degrading species *Enterococcus gallinarum*, previously isolated from NALB^70^ and the whole NALB community were assessed. Chosen substrates impacted oxalate metabolism and the impact of oxalate on growth, particularly at the community level (Fig. 5A,B). A minimal media with the same substrates added as sole carbon and energy sources (Table S7) allowed for quantification of the proportion of the NALB microbiota that could use each substrate as sole carbon and energy sources. Culturomic data recapitulates molecular data to show a considerable amount of redundancy surrounding oxalate metabolism (Fig. 5C). Isolates generated from this assay were used for subsequent study (metabolic cohort; Figure 5D). Additionally, a second cohort was defined and commercially purchased based both on known metabolic functions and the proportion of studies that saw an increase in their taxonomic population with oxalate consumption (Fig. 5D; taxonomic cohort). Where possible, isolates from human sources were obtained. Cohorts, defined in the STAR methods, were used to delineate hypotheses that either carbon and energy substrates are sufficient to explain known effects of the oxalate-degrading microbial network or that additional aspects of taxa commonly stimulated by dietary oxalate are required to explain past results (taxa defined through previous meta-analysis of studies)^15^. Oxalate metabolism with the metabolic and taxonomic cohorts was assessed *in vitro* in minimal media with oxalate as a sole carbon and energy source (Fig. 5E,F). There were considerable differences in oxalate metabolism in both cohorts, dependent on the microbes present. However, significant oxalate metabolism occurred even in the absence of *O. formigenes*, indicative of metabolic redundancy. Collectively, data show that both resource availability and community composition impacts oxalate metabolism, which helps to define the adaptive nature of the NALB gut microbiota. Additionally, results further bolster evidence for redundancy surrounding oxalate metabolism.

### Severity of oxalate-induced microbe-host effects is dependent on microbial oxalate metabolism, independent of taxonomy

**Fig. 6.**
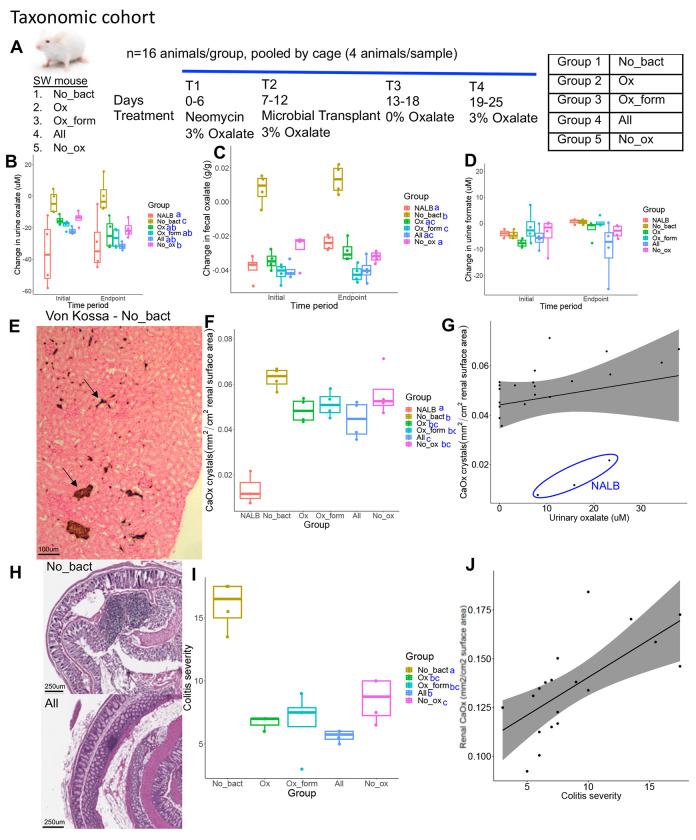
Microbial community composition (taxonomic cohort) impacted the effect of exogenous oxalate on host health. A) Swiss Webster mice were given neomycin, followed by inoculation of microbial consortia that included either no bacteria or the taxonomic cohort listed in Figure 5C. B,C) The effect of microbial transplants on urinary (B) or fecal (C) oxalate levels over the course of the diet trial, compared to baseline. ANOVA p<0.001 for microbial group, but was not significant by timeperiod or 2-way analyses for both B & C. D) The effect of microbial transplants on urinary formate levels over the course of the diet trial, compared to baseline. ANOVA was not significant in one-way and two-way analyses. E) Renal calcium oxalate deposition. Arrows show stained calcium deposits, which were quantified through an automated algorithm in QuPath. F) Quantification of renal calcium oxalate deposition by group. p<0.001, ANOVA. Blue letters reflect statistical groups between microbial groups for renal calcification by Holm’s corrected paired t-tests. G) Pearson correlation between urinary oxalate and renal calcium oxalate deposition. R=0.22, p=0.32 with NALB samples included (blue circle); R=0.7, p=0.001 excluding the NALB group. H) Representative colon tissues from the No_bact (Group 1) and All (Group 4) groups, exhibiting high and low colitis severity scores, respectively. Tissues were stained with hematoxylin and eosin and scored based on standardized, multifactorial metrics. I) Quantification of colitis severity by group. p<0.001, ANOVA. Blue letters reflect statistical groups between microbial groups for renal calcification by Holm’s corrected paired t-tests. J) Pearson correlation between colitis severity and renal calcium oxalate deposition. R=0.7, p=0.002. Colitis severity was not quantified for the NALB group.

To delineate hypotheses of metabolic redundancy or cooperation for mitigating the negative effects of oxalate on the gut microbiota and host, two independent diet trials were conducted with analogous microbial communities derived from the metabolic and taxonomic cohorts. Following antibiotic suppression of the gut microbiota, SWM were given microbial transplants from either the metabolic or taxonomic cohorts in a longitudinal, crossover experimental design with either a 0% or 3% oxalate diet (Envigo; Fig. 6A, S8A; Table S1). The 0% oxalate diet was designed to test the stability of oxalate-degraders since *O. formigenes* is often lost when oxalate is removed from the diet^15^. Transplanted microbial communities included the same as those for *in vitro* studies (Fig. 5D–F). Animal metrics and microbial were tracked over the course of the trial as was urinary/fecal oxalate, urinary formate, inflammatory cytokines, and creatinine. Renal calcium oxalate (CaOx) deposition, cardiac fibrosis, and colitis was assessed through histopathology. CaOx deposition and cardiac fibrosis was quantified through a semi-automated process, based on stain color that differentiates calcium deposits (Von Kossa) or collagen (Mason’s Trichrome). Colitis severity was assessed by two independent reviewers in hematoxylin and eosin stained tissues through a standardized, multi-factorial assessment^88^.

Using microorganisms from the taxonomic cohort, while the change in urinary and fecal oxalate levels were greatest in mice given *O. formigenes*, the change in oxalate levels were significantly greater than the no bacteria controls (Group 1) even in the absence of *O. formigenes*, consistent with *in vitro* results (Fig. 6B,C). Interestingly, the change in urinary formate levels was not different in any microbial group for the taxonomic cohort (Fig. 6D). While IL1β was below detection levels, IL6 exhibited levels consistent with oxalate induction, which decreased over the course of the trial (Fig. 6B, S6A). Differences in IL18 were only seen as an increase over time in the No_ox (Group 5) (Fig. S6B). The only changes in urinary creatinine seen was an increase and decrease in the NALB and All (Group 4) (Fig. S6C), respectively, which indicates that while the NALB bacteria may induce some inflammation, the minimal community present in the All (Group 4) group may limit these effects and actually improve kidney health under conditions of oxalate exposure. While some differences were seen in water or food metrics, these can largely be explained by batch effects of the two trials conducted (Fig. S6D–G). We did see a greater increase in body mass in animals receiving the No_ox (Group 5) microorganisms than when *O. formigenes* was administered alone (Group 2), which can not be explained by batch effects. Renal CaOx deposition, cardiac fibrosis, and colitis severity all closely tracked oxalate levels (Fig. 6E–J, S6H–I) and did not depend on *O. formigenes* if the other probiotic microbes were present. Collectively, data suggest that oxalate homeostasis and oxalate-induced pathologies are mitigated by the presence of diverse oxalate-degrading bacteria and that adding *O. formigenes* on an already effective oxalate-degrading microbiota will not improve oxalate homeostasis.

The microbiota composition of colon contents from this diet trial was significantly different from excreted feces (Fig. S7A). The microbiome composition in stool from mice was not different based on probiotic administration (Fig. S7B), but within group differences for the All (Group 4) group was significantly lower than other groups (Fig. S7C), indicative of a protective effect against oxalate exposure. This conclusion is corroborated by the change in alpha diversity in which the microbiota of animals given the All (Group 4) group exhibited the greatest post-antibiotic recovery and is the only group that saw recovery beyond a marginally significant increase in diversity, post-antibiotics (Fig. S7D). In general, there was not much loss of the inoculated bacteria throughout the study, in any group (Fig. S7E).

Overall, metabolic cohort transplants were less effective than the taxonomic cohort in terms of inducing oxalate metabolism, particularly in the group without bacteria (Group 1) isolated against oxalate (Figs. 6B,C, S8B,C). However, there was a much greater change in format metabolism, dependent on the microbes present (Fig. S8D). Despite the more moderate change in oxalate levels, there were similar oxalate-associated effects on renal calcium oxalate deposition (Fig. S8F,G), which was not dependent on formate levels (Fig. S8H). With the metabolic cohorts, the presence of known oxalate degraders appeared to be more important for oxalate homeostasis than the taxonomic cohort, since absence of known oxalate-degraders led to oxalate levels similar to the No_bact (Group 1) controls (Figs. 6B,C; S8B,C). Collectively, data show that while the taxa chosen for the taxonomic cohort enabled efficient oxalate even in the absence of *O. formigenes*, the bacteria present in the metabolic cohort had a greater influence on formate levels. In both diet trials, urinary oxalate, but not formate correlated with CaOx deposition (Fig. S8G,H – metabolic, data not shown - taxonomic). The effects of oxalate and transplant group differed between the two cohort studies with mice in the metabolic cohort exhibiting higher levels of IL-6 of microbial transplant groups compared to the negative controls. However, similar to the taxonomic cohort, there were no differences in IL18. Urinary creatinine increased significantly over time for all groups receiving a microbial transplant, in contrast with the taxonomic cohort (Figs. S6A–C,S9A–C). For the metabolic cohort, there similar group-based differences observed in water based metrics. However, animals in the taxonomic cohort exhibited greater positive changes in food intake and body mass than the metabolic cohort (Fig. S9D–G).

In the metabolic cohort, we did not observe the same trends in the microbiota composition, antibiotic recovery, or persistence as we did in the taxonomic trial indicative of less effective microbial communities at protecting the community as a whole (Fig. S10A–D), though probiotic bacteria were generally persistent (Fig. S10E).

Overall, data from the taxonomic and metabolic cohorts indicate that oxalate metabolism results from a defined community of microorganisms that includes redundancy in oxalate metabolism as the primary driver. Importantly, the efficacy of *O. formigenes* was apparent when administered alone, but the effect was diminished when co-inoculated with other oxalate-degrading microbes.

## Discussion

While research in the last two decades have made clear that the gut microbiota is intimately tied to all facets of host health, translating those insights into actionable biotherapies has been difficult due to the inherent complexity and heterogeneity present in the microbe-host system. The study of complex systems holds enormous potential to constrain hypotheses and offer insight into effective bacteriotherapy development^23^. However, the field is limited by inconsistent terminologies, concepts, and tractable experimental frameworks^25^. The objective of the current study was to examine oxalate-microbe-host interactions within a complex systems framework. Collectively, results of the study offer quantifiable and generalizable metrics of diet-microbe-host systems that can be used to guide more effective patient and probiotic selection criteria for clinical trials involving bacteriotherapies that target the gut microbiota.

Microbial oxalate metabolism, which has been researched for several decades, predating the current microbiome era, is an ideal focal point to understand pertinent variables that impact probiotic success. Here, we found that heterogeneity in microbiome composition, independent of host genetics, significantly impacted multiple diet-microbe-host interfaces that included hepatic activity (Figs. 1,2), gut microbiota metabolism (Figs. 2,3), renal mineralization (Fig. 6E,F; S8E,F), intestinal inflammation (Fig. 6H,I), and cardiac fibrosis (Fig. S6H,I). Importantly, we found that oxalate degradation was highly redundant among diverse species in the gut of *N. albigula*, which consumes a high oxalate diet in the wild (Fig. 4). Consistent with past studies, administering *O. formigenes* led to a significant reduction in both stool and urine oxalate (Fig. 6B,C). However, when co-administered with other oxalate-degrading microorganisms, there was no additive effect. Oxalate-based results were similar, but to a lower degree, in the metabolic cohort (Fig. S8B,C). In taxonomic cohort studies, we did not see significant differences in the change in urine formate, based on microbial transplant group (Fig. 6D), in contrast to metabolic cohort studies (Fig. S8D). Collectively, data offer strong support for the hypothesis that metabolic redundancy among diverse taxa, is the primary driver of oxalate homeostasis, rather than metabolic cooperation in which the by-products of oxalate degradation are used in downstream pathways such as acetogenesis, methanogenesis, and sulfate reduction. However, data on the metabolic cooperation hypothesis were inconclusive and there are multiple known microbe and host sources of formate, which may confound results in these studies^74,89–91^.

Modelling the microbe-host system through an evolutionary perspective, it has previously been suggested that while host control over the microbiome is the primary driver of the form and function of the gut microbiota, given the enormous biomass and diversity of the gut microbiota, microbe-microbe competition is also required to constrain the microbiota in mammalian microbe-host systems^21^. In the current (Figs. 2B, 5B) and previous studies^33,34,64,65^, we and others have found that oxalate can differentially exhibit positive or negative effects on microbial growth and metabolism dependent on the species and environment present. These data provide two alternative ecological pressures to degrade oxalate. The first is to use oxalate as a carbon and energy source for growth, as is the case with *O. formigenes*^33^. The second is to degrade oxalate to remove it as a toxin, as is the case with some *Lactobacilli* and *Bifidobacteria*^64,65^. The data showing oxalate degradation as a metabolically redundant function among multiple diverse microorganisms that maintains oxalate homeostasis (Figs. 4, 6, S8), suggest that the amount of oxalate consumed or produced by the liver is too great for a single, slow-growing oxalate-degrading specialist can handle alone, in contrast to prevailing hypotheses^63^, and in support of the ecosystem on a leash hypothesis. Importantly, the near universal presence of formate metabolism genes suggest that formate may be an even greater source of ecological pressure (Figs. S2–S5). Collectively, data from the current and previous studies on the effect of oxalate exposure on the gut microbiota^68,71^ support the hypothesis that the gut microbiota serves as an adaptive organ^92–94^ in which specific, metabolically redundant microbes respond to and eliminate dietary components, for the benefit of themselves, but which can residually protect or harm host health depending on the dietary molecules and gut microbiota composition^6,92–94^.

Oxalate degradation, as a focal point in diet-microbe-host interactions, is a special case in which the effects of oxalate on host health and the mechanisms of microbial oxalate degradation have largely been worked out for decades^33,51–54^. Furthermore, oxalate degradation exhibits moderate complexity, both in that it is isolated to the gut microbiota and is performed through a handful of simple metabolic pathways^31–35^. Despite this knowledge, clinical trials designed to reduce systemic oxalate using either *O. formigenes* or other oxalate-degrading bacteria have exhibited a wide variety of patient and trial responses^15^. However, knowledge about other diet-microbe-host links are largely in their infancy. Many of the other known features exhibit much greater complexity than oxalate degradation. For instance, while the production of short chain fatty acids are widely viewed as beneficial for both the gut microbiota and host health^95–100^, many short chain fatty acids are known and are produced by a wide variety of species^101,102^. Other important diet-microbe-host links, such as the production of secondary bile acids or TMAO^6,36,103–105^, also involve host hepatic activity, increasing the complexity further. Given the complexity of diet-microbe-host interactions and the fact that understanding the mechanisms through which the gut microbiota modifies the diet to influence host health is demonstrably insufficient for the development of effective bacteriotherapies^12–15^, we propose a three-phase, pre-clinical experimental workflow for the development of targeted bacteriotherapies. In the first phase of research, case:control metagenome-wide studies and research into other factors that modify the microbiome, such as prior antibiotic exposure, can identify disease phenotypes that are influenced by the microbiome, along with the microbial taxa and genes responsible for this association. In the second phase of research, hypothesized taxa and/or metabolic functions can be explored through *in vitro* and germ-free animal studies, using appropriate models to determine the mechanistic links that drive disease phenotypes through diet-microbe-host interactions. Finally, in phase three, these mechanistic links can be applied to a complex system theoretical framework, as done in the current study, to identify those variables most pertinent to successfully influence specific phenotypes through targeted changes to the gut microbiota. Such a phased research structure will provide for much more effective probiotic and patient selection criteria prior to clinical trials.

There were some important limitations to the current study. First, while we did conduct one study with *N. albigula*, most animal studies here sought to eliminate host genetics from the equation, as a means of simplification. Host genetics are another layer of complexity that were not examined here and may have an impact on oxalate homeostasis beyond the gut microbiota. Second, in animal studies with the refined communities of microorganisms, the taxonomic and metabolic cohorts studies were conducted separately and some batch effects are apparent, such as with the change in creatinine values or urine output. Finally, it is apparent from our data and the literature that there are multiple host and microbial sources of formate. As such, data pertaining to formate utilization by our transplant communities are inconclusive.

In conclusion, using a complex systems theoretical framework, we examined the oxalate-microbe-host interactions of multiple oxalate-microbe-host interfaces and found multiple microbiome-dependent effects of oxalate. The negative effects of oxalate were mitigated by metabolically redundant oxalate-degrading bacteria, more so than by metabolic cooperation or a single oxalate-degrading species. Critically, we found that while *O. formigenes* can lower urine oxalate when placed on a background of a poor oxalate-degrading community, as current theory predicts, this effect is lost when coadministered with other oxalate-degrading bacteria. Collectively, data help to resolve why the gut microbiota of the white throated woodrat, *Neotoma albigula*, effectively responds to and degrades even very high dietary oxalate levels^68,69,72^ and offer a clear pathway for more effective patient and probiotic selection for future clinical trials to reduce urine oxalate. More importantly, the conceptual and experimental framework developed in this study, based on complex systems theory, paves the way for a phased approach to microbiome research in which clinical microbiome insights (Phase I) drive mechanistic insights between the gut microbiota and host physiology (Phase II), that can be applied to a complex system model to constrain hypotheses and identify the most pertinent variables that drive microbe-host interactions that influence host physiology and health (Phase III). Such an approach will allow for much more efficacious probiotic and patient selection criteria in clinical intervention trials.

## Supplementary Material

1

## Data Availability

Sequence reads from the animal study are available at the Sequence Read Archive under Accession numbers for gut microbiota genomes: PRJNA833303, Shotgun metagenomics: PRJNA839366, Liver transcriptomics: PRJNA1018952 Gut microbiota data: 16SPRJNA1018559
