# Supplementary material for: Complex system modelling reveals oxalate homeostasis is driven by diverse oxalate-degrading bacteria
Source: bioRxiv. 2025 Feb 19:2024.10.28.620613. Originally published 2024 Oct 28. Preprint. [Version 2] doi: 10.1101/2024.10.28.620613 (PMC11565779; doi:10.1101/2024.10.28.620613)
Supplement: Supplement 6 [file NIHPP2024.10.28.620613v2-supplement-6.pdf]

bioRxiv preprint doi: https://doi.org/10.1101/2024.10.28.620613; this version posted February 19, 2025. The copyright holder for this preprint (which was not certified by peer review) is the author/funder, who has granted bioRxiv a license to display the preprint in perpetuity. It is made available under aCC-BY 4.0 International license.

Supplemental information titles and legends

Supplementary Figures and table legends
Figs. S1 to S10
Tables S1 and S9

Supplementary figure legends:

Fig. S1. Phylogeny of genomes extracted from the *N. albigula* metagenome, in comparison to >3000 full length microbial genomes. Stars indicate taxonomic placement of extracted genomes. All other points indicate reference genomes.

Fig. S2. Representation of formate metabolism genes in the NALB metagenome derived from 248 full length genomes. A) Proportion of the genomes extracted from *N. albigula* that had at least one formate metabolism gene. B) Representation of formate metabolism genes among the metagenome, broken down by gene function and proportion of total formate metabolism genes. C) Representation of formate metabolism genes broken down by taxon.

Fig. S3. Representation of acetogenic genes in the NALB metagenome derived from 248 full length genomes. A) Proportion of the genomes extracted from *N. albigula* that had at least one acetogenic gene. B) Representation of acetogenic genes among the metagenome, broken down by gene function and proportion of total acetogenic genes. C) Representation of acetogenic genes broken down by taxon.

Fig. S4. Representation of methanogenic genes in the NALB metagenome derived from 248 full length genomes. A) Proportion of the genomes extracted from *N. albigula* that had at least one methanogenic gene. B) Representation of methanogenic genes among the metagenome, broken down by gene function and proportion of total methanogenic genes. C) Representation of methanogenic genes broken down by taxon.

Fig. S5. Representation of sulfate-reducing genes in the NALB metagenome derived from 248 full length genomes. A) Proportion of the genomes extracted from *N. albigula* that had at least one sulfate-reducing gene. B) Representation of sulfate-reducing genes among the metagenome, broken down by gene function and proportion of total sulfate-reducing genes. C) Representation of sulfate-reducing genes broken down by taxon.

Fig. S6. Microbial transplant composition impact on urinary inflammation, renal health, and overall mouse health for the taxonomic cohort. A-G) The effect of microbial transplants on urinary IL-6 (A), IL-18 (B), and creatinine (C) levels, along with water intake (D), urine output (E), food intake (F) and body mass (G). H) Masson's Trichrome staining of heart tissue reveals fibrosis. Shown are representative images from the No_bact (Group 1) and All (Group 4) groups. I) Quantification of cardiac fibrosis from

bioRxiv preprint doi: https://doi.org/10.1101/2024.10.28.620613; this version posted February 19, 2025. The copyright holder for this preprint (which was not certified by peer review) is the author/funder, who has granted bioRxiv a license to display the preprint in perpetuity. It is made available under aCC-BY 4.0 International license.

different microbial transplant groups. Statistical significance - 2-way ANOVA (shown on charts) and post-hoc, Holm's corrected, paired t-tests. Blue letters reflect statistical groups between microbial transplants and *p<0.05, **p<0.01; ***p<0.001 for longitudinal comparisons within each microbial transplant group.

Fig. S7. Effect of microbial transplants on microbial community composition for the taxonomic cohort. A,B) Beta-diversity analysis based on a weighted UniFrac dissimilarity matrix of colon feces collected at the end of the study period vs. stool (A; . p=0.006, PERMANOVA) or in the endpoint stool samples by group (B; no significant differences). Similar results for (B) were obtained with colon feces. Statistical differences shown by blue letters in legend. C) Within group beta-diversity distance of colon feces. p=0.006, one-way ANOVA. Blue letters represent statistical groups, based on post-hoc, Holm's corrected, paired t-tests. Similar results were found with endpoint stool samples. D) Change in phylogenetic diversity of stool for each microbial transplant group. Stats, shown on graph, are based on Pearson correlations. E) Normalized counts of transplanted microorganisms in the stool across timepoints and in the colon feces at the end of the diet trial.

Fig. S8. Microbial community composition (metabolic cohort) impacted the effect of exogenous oxalate on kidney health. A) Experimental design. Swiss Webster mice were given neomycin, followed by inoculation of one of five microbial consortia that included either no bacteria, the NALB community, or the metabolic cohort listed in Figure 5C. Animals were maintained on a 3% oxalate diet throughout the trial except for a 0% oxalate washout period after the microbial transplant period. B,C) The effect of microbial transplants on urinary (B) or fecal (C) oxalate levels over the course of the diet trial, compared to baseline. ANOVA p<0.001 for microbial group (B&C), and p<0.01 for both timeperiod and in 2-way analysis for (B only). D) The effect of microbial transplants on urinary formate levels over the course of the diet trial. ANOVA p<0.001 for microbial group and timeperiod. E) Renal calcium oxalate deposition based on Von Kossa staining of renal tissue sections. Arrows show calcium deposits stained black, which were quantified through an automated algorithm in QuPath. F) Quantification of renal calcium oxalate deposition by group. p=0.014; ANOVA. G,H) Pearson correlation between urinary oxalate (R=0.7, p<0.001) (G) or formate (R=0.026, p=0.9) (H) and renal calcium oxalate deposition. Where applicable, statistical significance was assessed through an ANOVA and post-hoc, Holm's corrected, paired t-tests. Statistical groups shown by blue letters.

Fig. S9. Microbial transplant composition impact on urinary inflammation, renal health, and overall mouse health for the metabolic cohort. A-G) The effect of microbial transplants on urinary IL-6 (A), IL-18 (B), and creatinine (C) levels, along with water intake (D), urine output (E), food intake (F) and body mass (G). Statistical significance - 2-way ANOVA (shown on charts) and post-hoc, Holm's corrected, paired t-tests. Blue letters reflect statistical groups between microbial transplants and *p<0.05, **p<0.01; ***p<0.001 for longitudinal comparisons within each microbial transplant group.

bioRxiv preprint doi: https://doi.org/10.1101/2024.10.28.620613; this version posted February 19, 2025. The copyright holder for this preprint (which was not certified by peer review) is the author/funder, who has granted bioRxiv a license to display the preprint in perpetuity. It is made available under aCC-BY 4.0 International license.

Fig. S10. Effect of microbial transplants on microbial community composition for the metabolic cohort. A,B) Beta-diversity analysis based on a weighted UniFrac dissimilarity matrix of colon feces collected at the end of the study period vs. stool (A; . p=0.006, PERMANOVA) or in the endpoint stool samples by group (B; no significant differences). Similar results for (B) were obtained with colon feces. Statistical differences shown by blue letters in legend. C) Within group beta-diversity distance of colon feces. p=0.006, one-way ANOVA. Blue letters represent statistical groups, based on post-hoc, Holm's corrected, paired t-tests. Similar results were found with endpoint stool samples. D) Change in phylogenetic diversity of stool for each microbial transplant group. Stats, shown on graph, are based on Pearson correlations. E) Normalized counts of transplanted microorganisms in the stool across timepoints and in the colon feces at the end of the diet trial.

Fig. S11. Effect of oxalate and formate on oxalate quantification using an enzymatic, ELISA-based assay. A) Correlation between oxalate or oxalate + formate, added to water, on oxalate concentration quantified through the enzymatic kit. B) Ratio of the calculated oxalate vs. the added concentration of oxalate and formate. C) The effect of adding oxalate or formate to urine, or of extracting oxalate from urine. R values and p-values (A) or p-values from one-way ANOVA (B,C) shown on chart. Blue letters represent statistical groups, based on pairwise t-tests.

bioRxiv preprint doi: https://doi.org/10.1101/2024.10.28.620613; this version posted February 19, 2025. The copyright holder for this preprint (which was not certified by peer review) is the author/funder, who has granted bioRxiv a license to display the preprint in perpetuity. It is made available under aCC-BY 4.0 International license.

Supplementary legends

Table S1. Diets used in animal studies.

Table S2. The number of host hepatic genes in each metabolic pathway that exhibited a significant increase (positive numbers) or decrease (negative numbers) in expression upon consumption of a 1.5% oxalate diet.

Table S3. The number of microbial metabolites in each metabolic pathway that exhibited a significant increase (positive numbers) or decrease (negative numbers) in expression upon consumption of a 1.5% oxalate diet by the host.

Table S4. The significant positive and negative correlations between microbial metabolites and host hepatic gene expression for those metabolites/genes significantly altered by 1.5% oxalate consumption. Data are listed with the host microbiome, metabollite and gene ID, long with correlation values and false discovery rate corrected p-values.

Table S5. The number of microbial genes in each metabolic pathway that exhibited a significant increase (positive numbers) or decrease (negative numbers) in abundance upon consumption of a 6% oxalate diet by the host.

Table S6. Media recipe used to test the impact of substrates on oxalate metabolism.

Table S7. Media recipe used to test the proportion of the NALB community that can utilize substrates as sole carbon and energy sources.

Table S8. Microorganisms used in the taxonomic cohort.

Table S9. Information for 248 complete genomes extracted from the shotgun metagenomic data from the *N. albigula* gut microbiota.

bioRxiv preprint doi: https://doi.org/10.1101/2024.10.28.620613; this version posted February 19, 2025. The copyright holder for this preprint (which was not certified by peer review) is the author/funder, who has granted bioRxiv a license to display the preprint in perpetuity. It is made available under aCC-BY 4.0 International license.

Supplementary Figures

Fig. S1

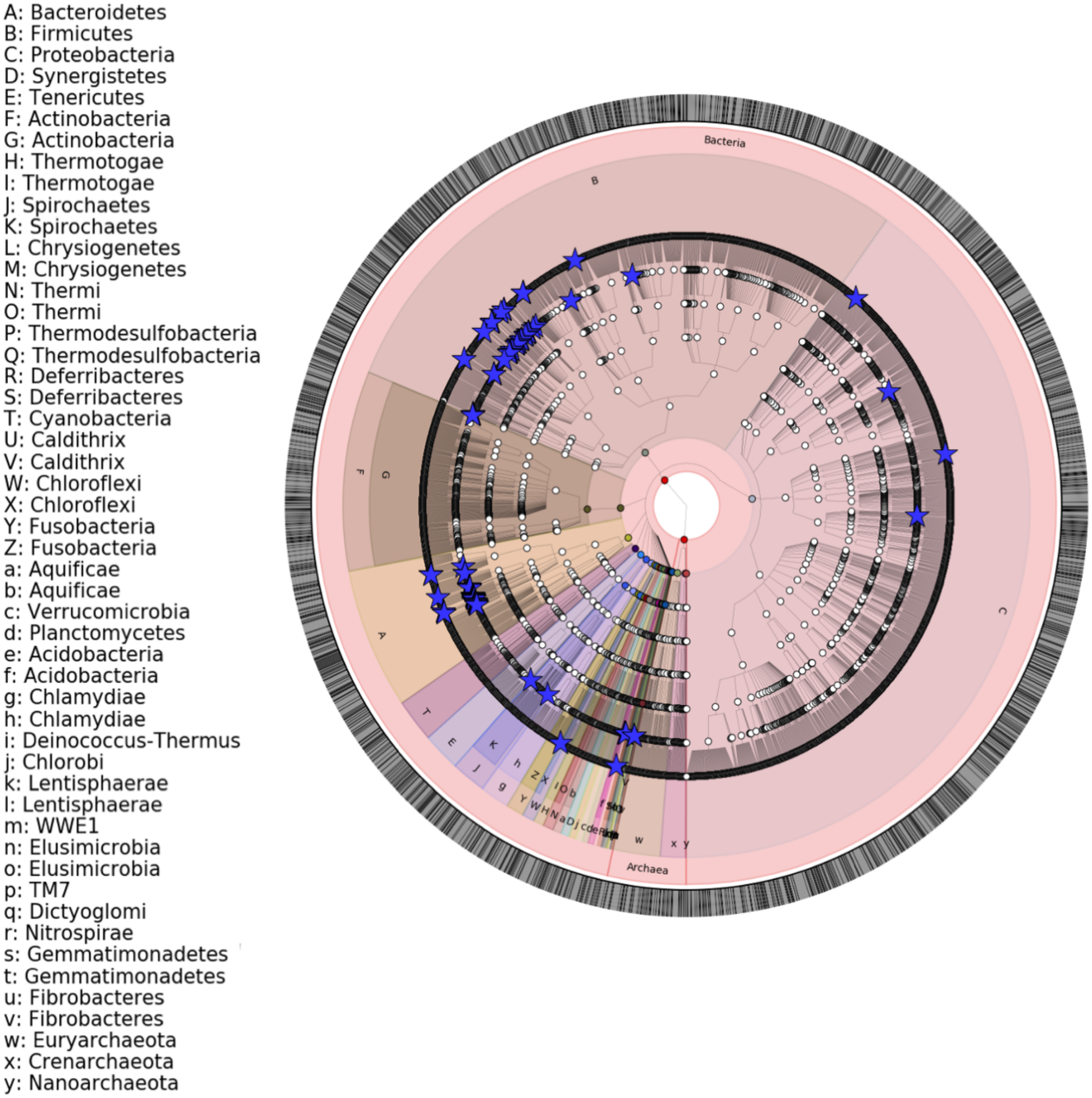

.

bioRxiv preprint doi: https://doi.org/10.1101/2024.10.28.620613; this version posted February 19, 2025. The copyright holder for this preprint (which was not certified by peer review) is the author/funder, who has granted bioRxiv a license to display the preprint in perpetuity. It is made available under aCC-BY 4.0 International license.

Fig. S2

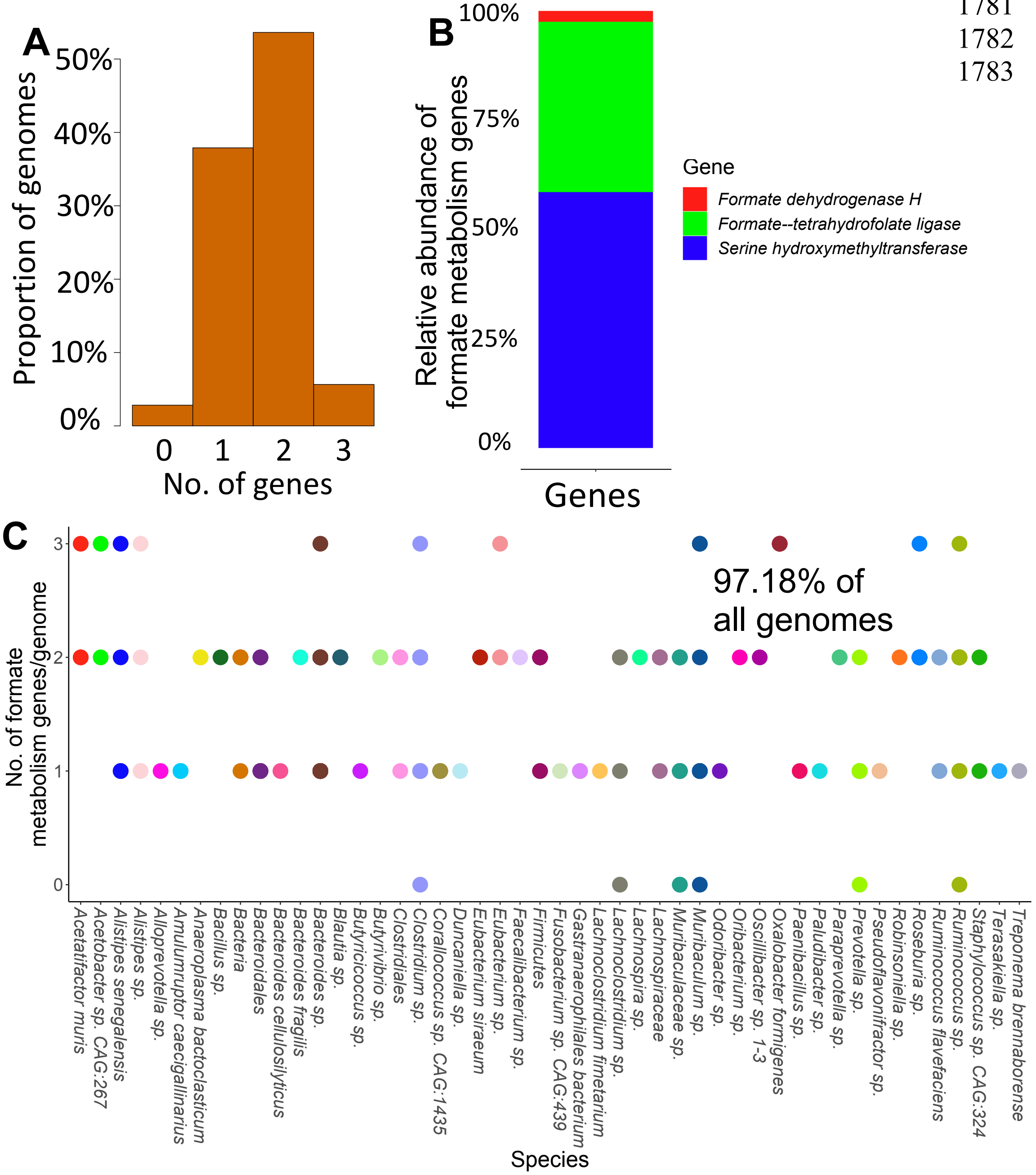

bioRxiv preprint doi: https://doi.org/10.1101/2024.10.28.620613; this version posted February 19, 2025. The copyright holder for this preprint (which was not certified by peer review) is the author/funder, who has granted bioRxiv a license to display the preprint in perpetuity. It is made available under aCC-BY 4.0 International license.

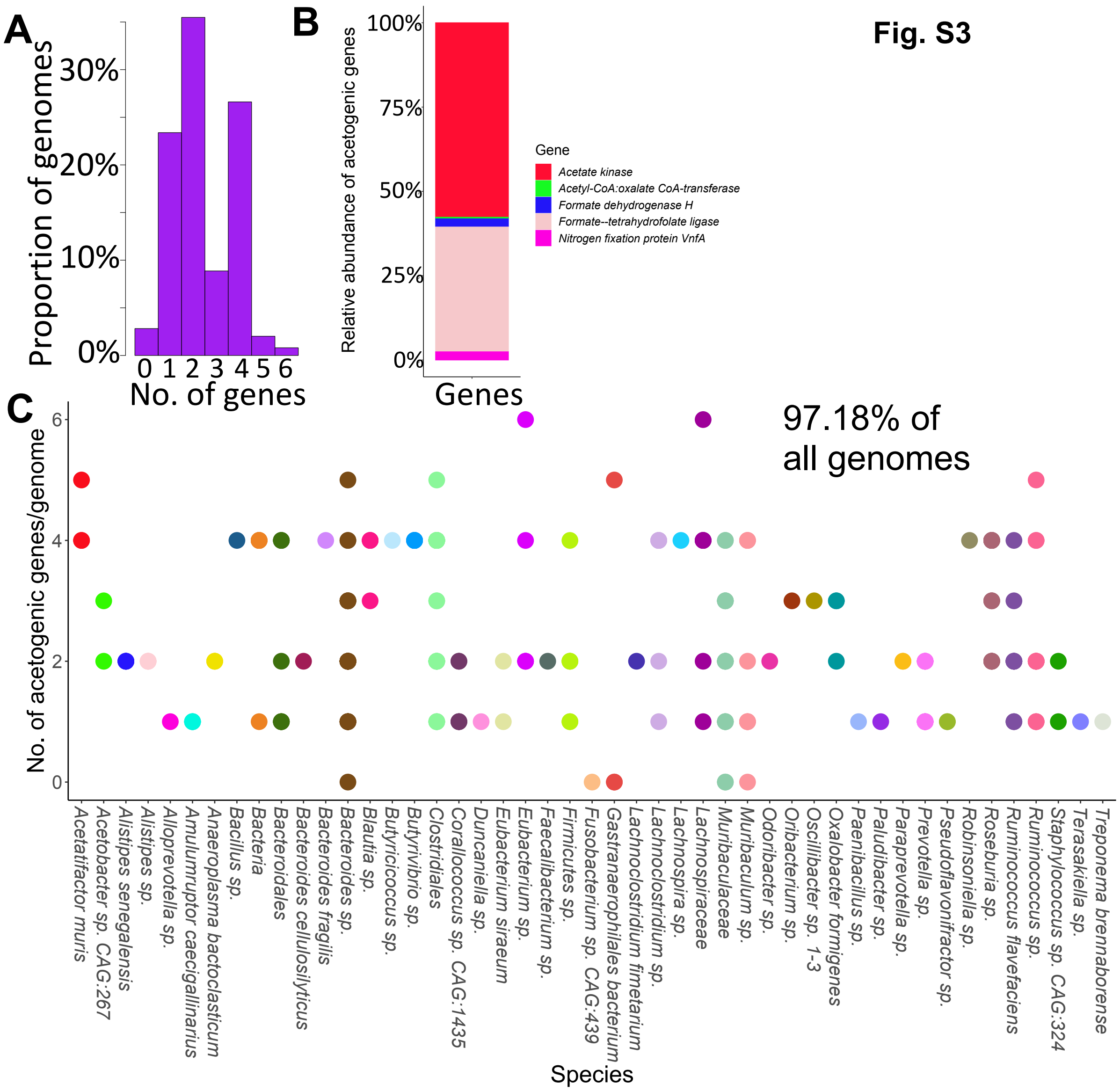

1784

bioRxiv preprint doi: https://doi.org/10.1101/2024.10.28.620613; this version posted February 19, 2025. The copyright holder for this preprint (which was not certified by peer review) is the author/funder, who has granted bioRxiv a license to display the preprint in perpetuity. It is made available under aCC-BY 4.0 International license.

Fig. S4

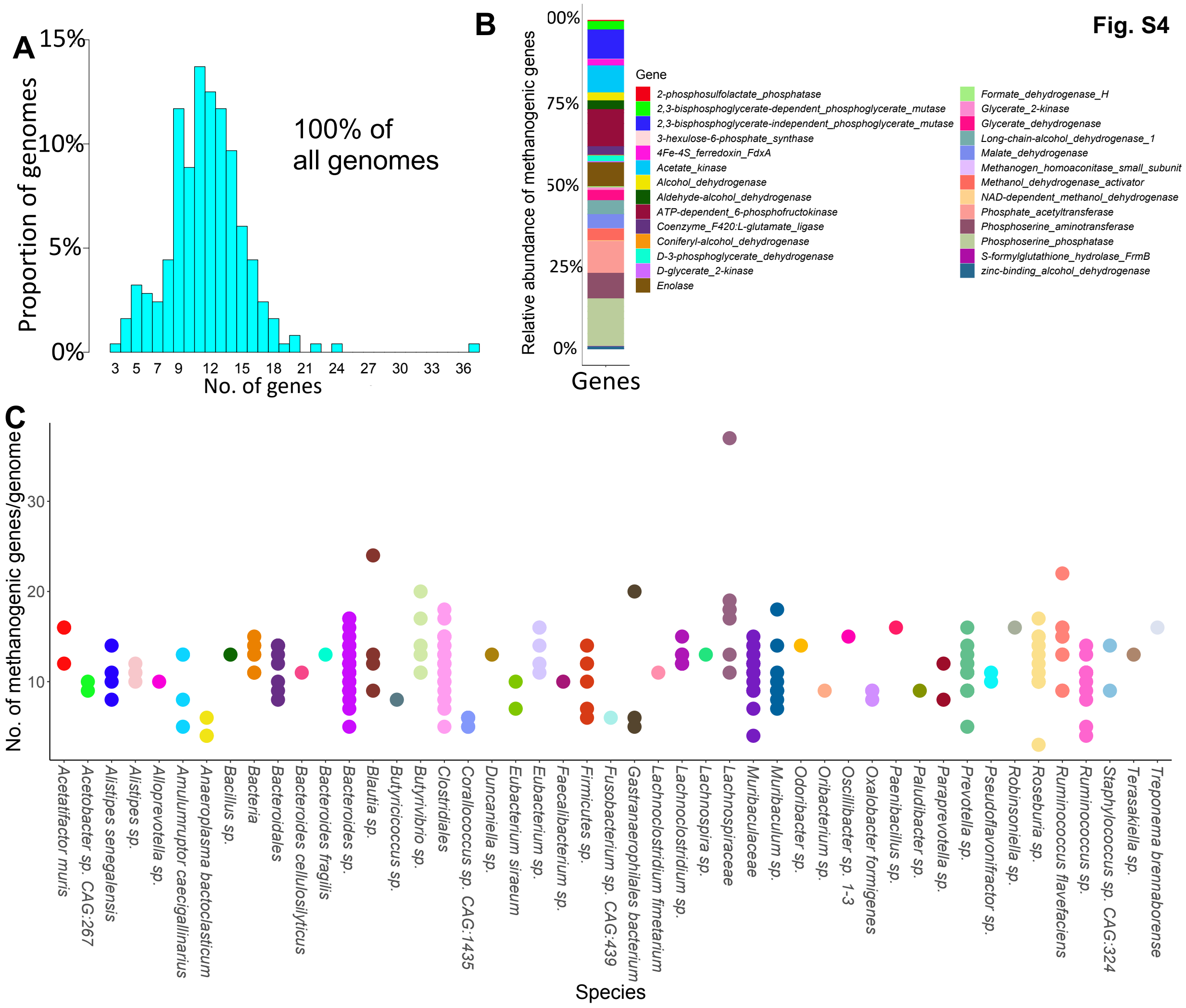

bioRxiv preprint doi: https://doi.org/10.1101/2024.10.28.620613; this version posted February 19, 2025. The copyright holder for this preprint (which was not certified by peer review) is the author/funder, who has granted bioRxiv a license to display the preprint in perpetuity. It is made available under aCC-BY 4.0 International license.

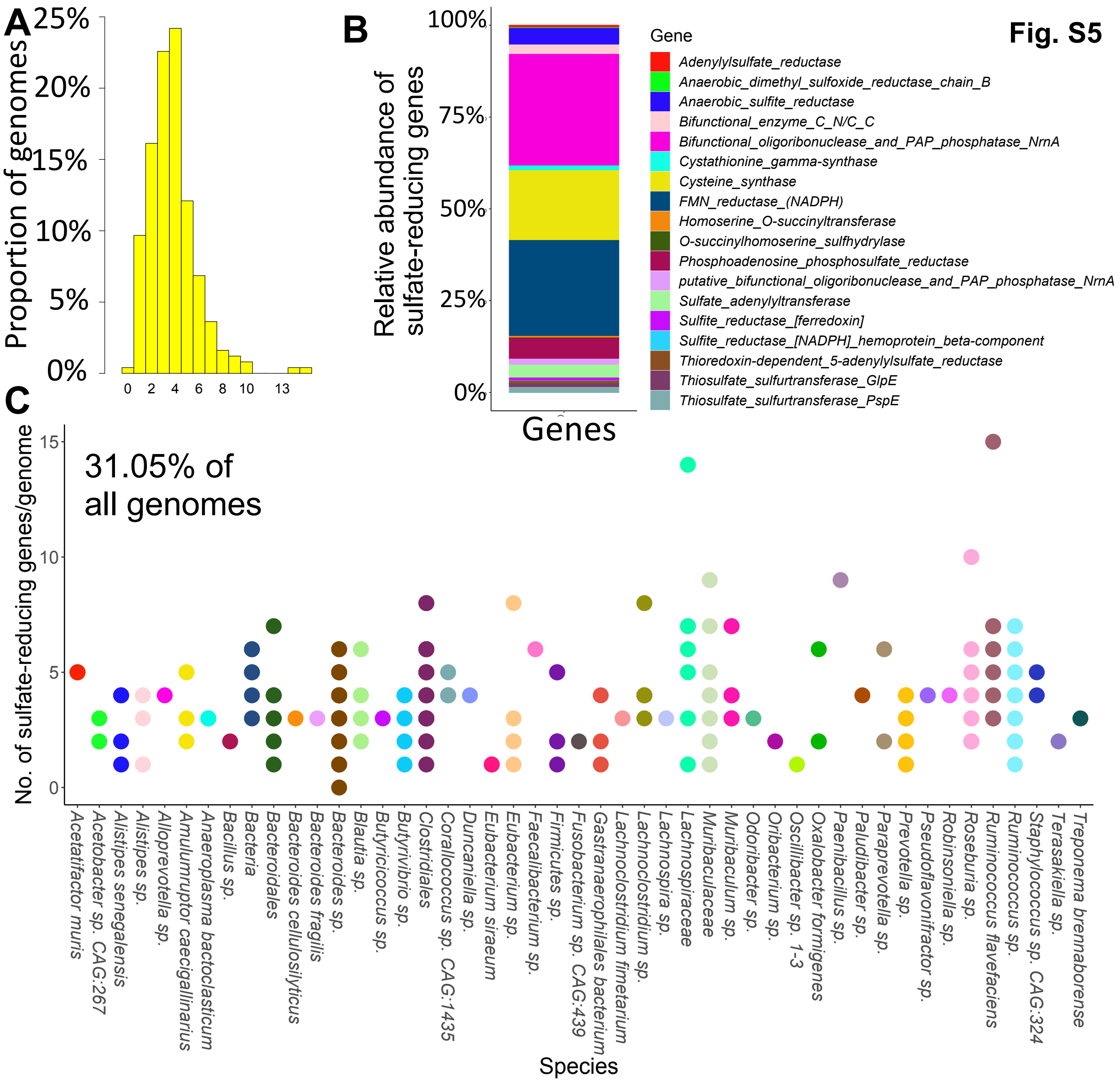

bioRxiv preprint doi: https://doi.org/10.1101/2024.10.28.620613; this version posted February 19, 2025. The copyright holder for this preprint (which was not certified by peer review) is the author/funder, who has granted bioRxiv a license to display the preprint in perpetuity. It is made available under aCC-BY 4.0 International license.

1787

Taxonomic cohort

Fig. S6

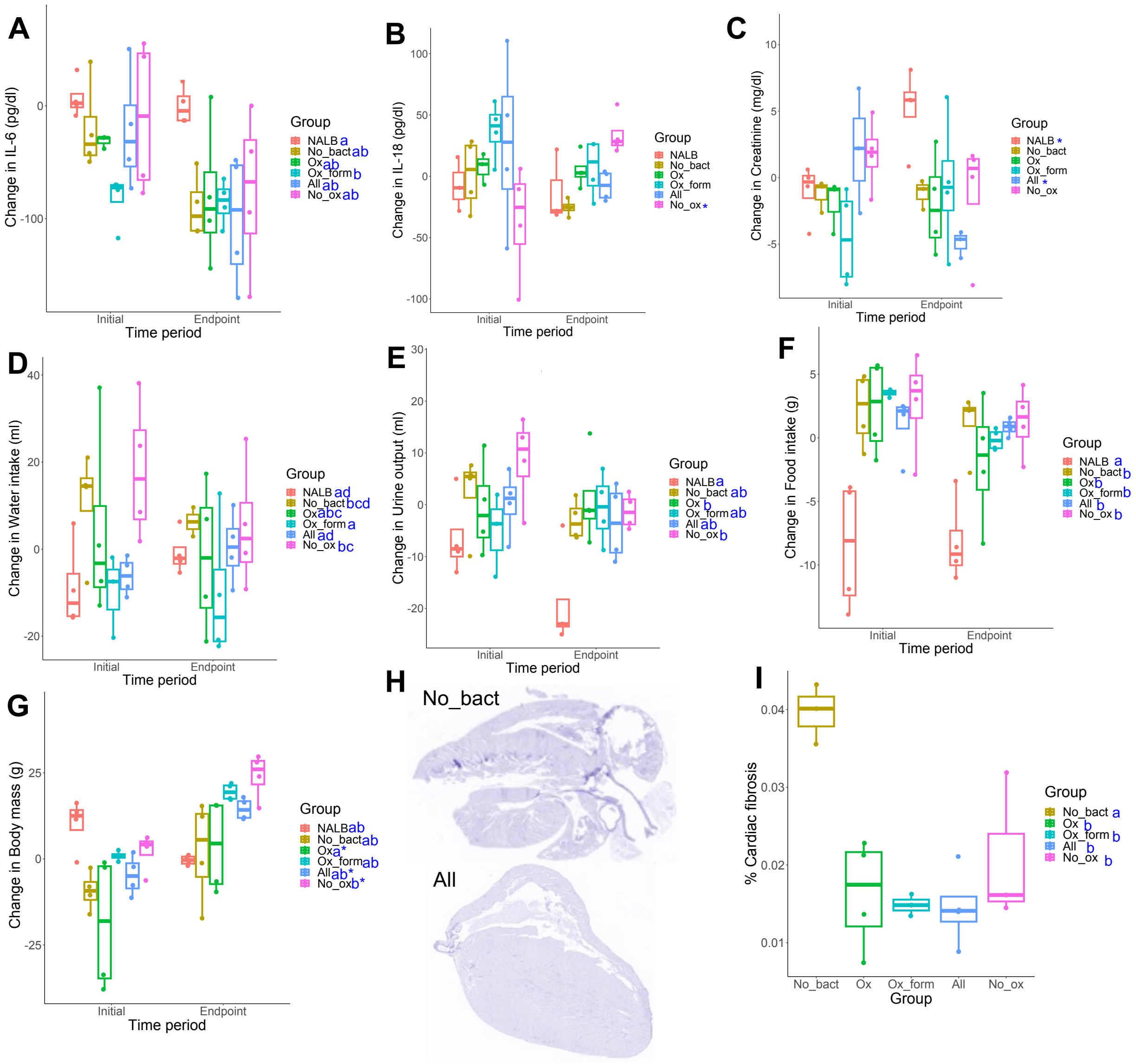

Group 1	No_bact
Group 2	Ox
Group 3	Ox_form
Group 4	All
Group 5	No_ox

1788
1789

bioRxiv preprint doi: https://doi.org/10.1101/2024.10.28.620613; this version posted February 19, 2025. The copyright holder for this preprint (which was not certified by peer review) is the author/funder, who has granted bioRxiv a license to display the preprint in perpetuity. It is made available under aCC-BY 4.0 International license.

Taxonomic cohort

Fig. S7

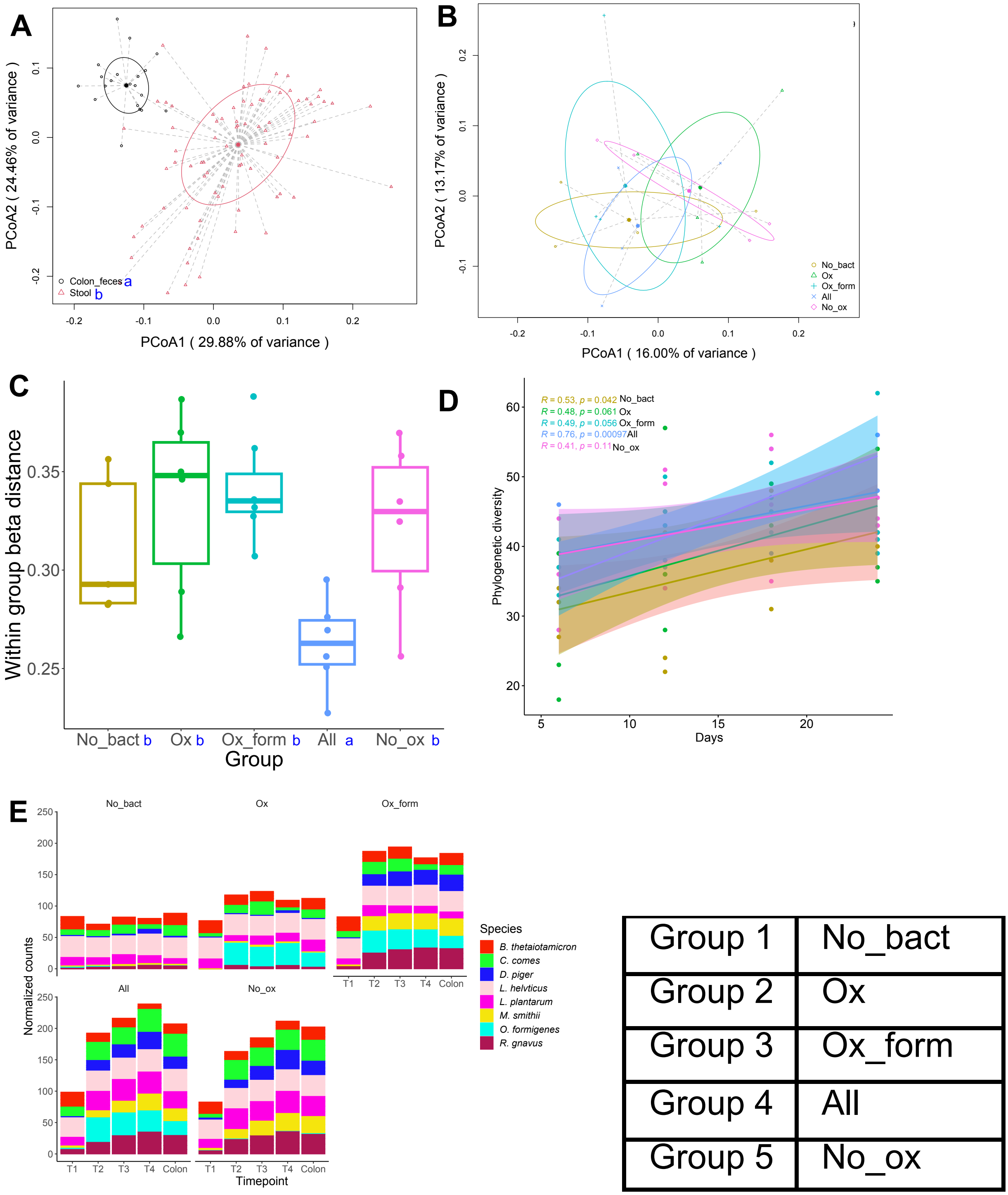

Group 1	No_bact
Group 2	Ox
Group 3	Ox_form
Group 4	All
Group 5	No_ox

bioRxiv preprint doi: https://doi.org/10.1101/2024.10.28.620613; this version posted February 19, 2025. The copyright holder for this preprint (which was not certified by peer review) is the author/funder, who has granted bioRxiv a license to display the preprint in perpetuity. It is made available under aCC-BY 4.0 International license.

Metabolic cohort

Fig. S8

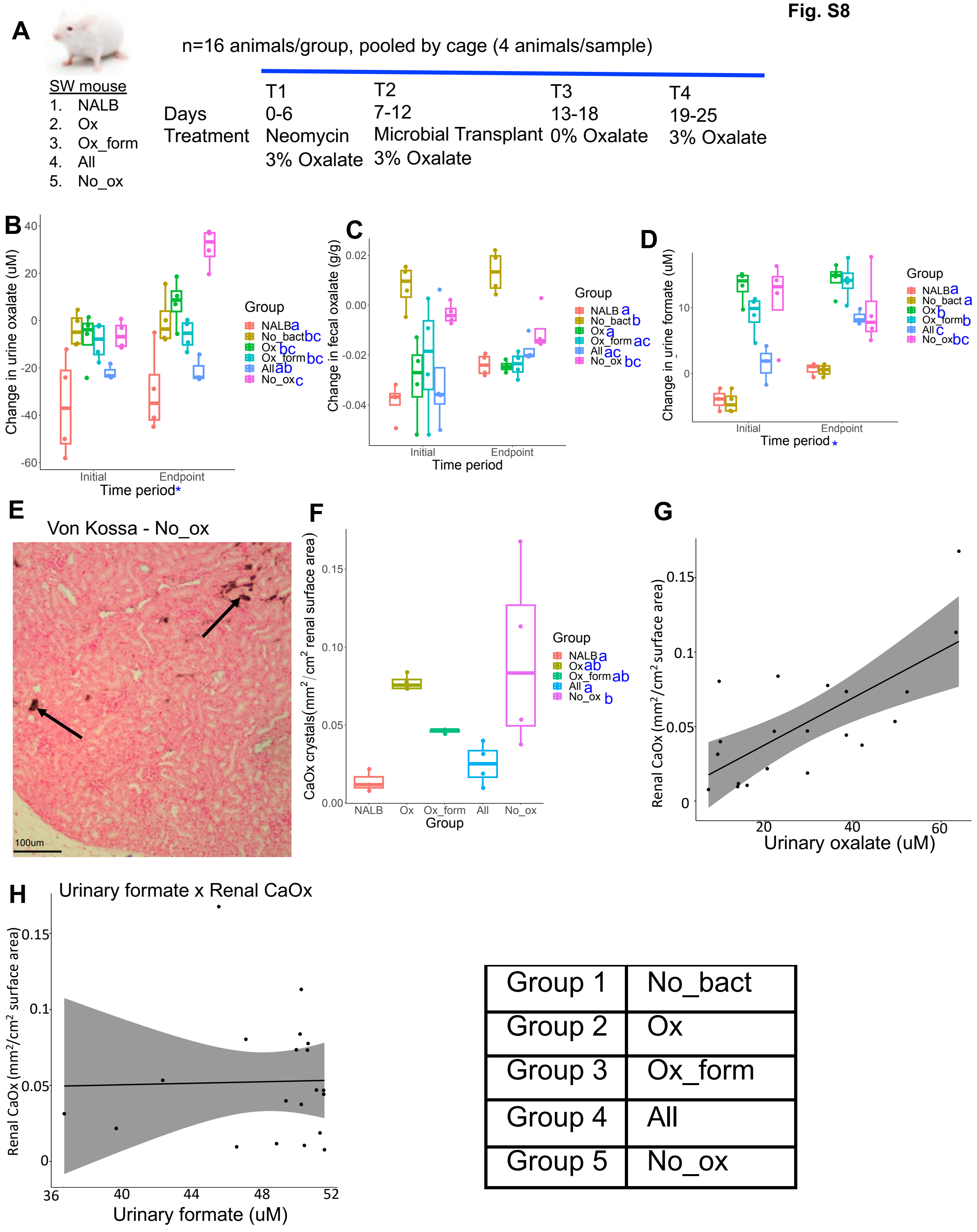

Group 1	No_bact
Group 2	Ox
Group 3	Ox_form
Group 4	All
Group 5	No_ox

bioRxiv preprint doi: https://doi.org/10.1101/2024.10.28.620613; this version posted February 19, 2025. The copyright holder for this preprint (which was not certified by peer review) is the author/funder, who has granted bioRxiv a license to display the preprint in perpetuity. It is made available under aCC-BY 4.0 International license.

Metabolic cohort

Fig. S9

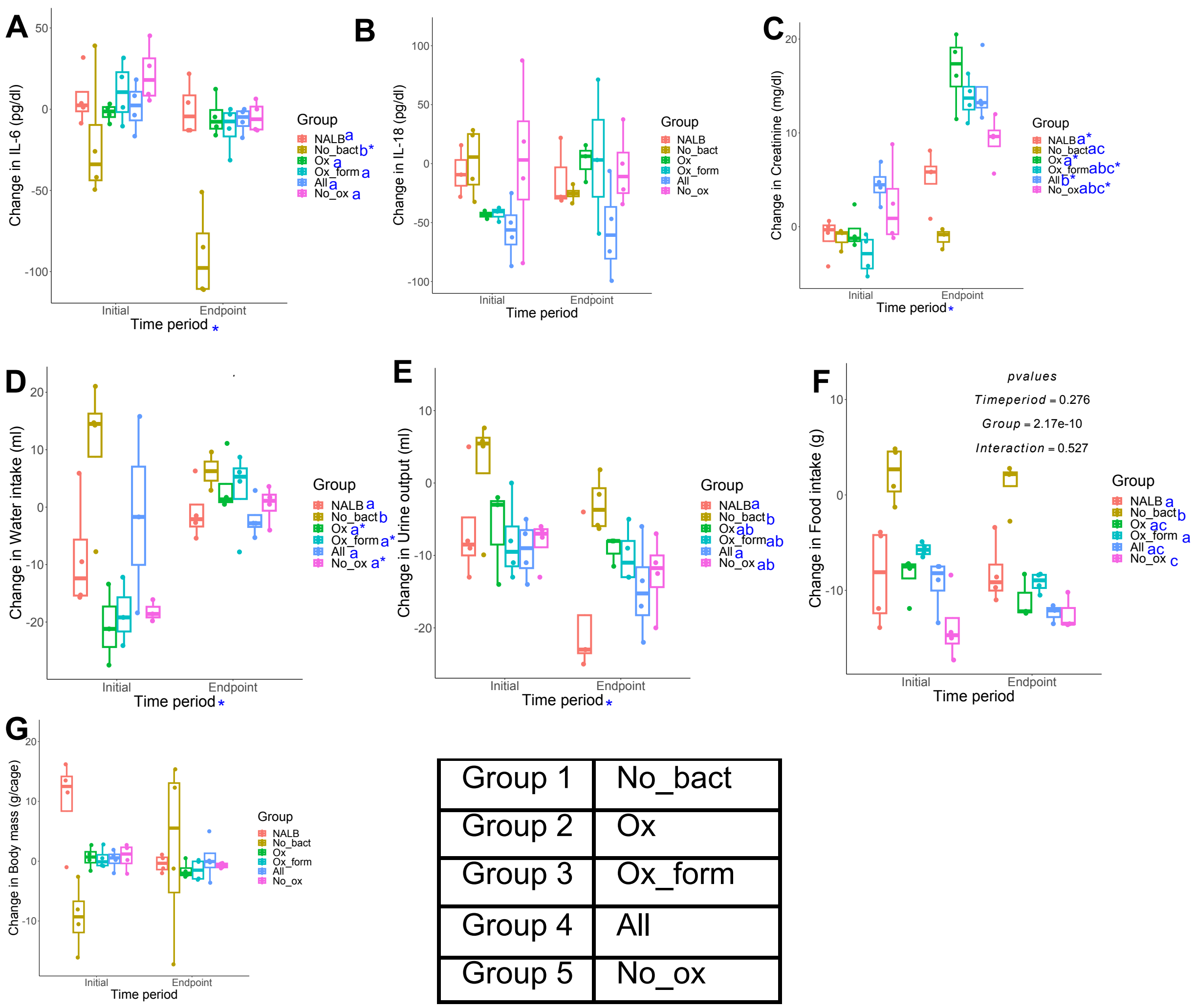

Group 1	No_bact
Group 2	Ox
Group 3	Ox_form
Group 4	All
Group 5	No_ox

bioRxiv preprint doi: https://doi.org/10.1101/2024.10.28.620613; this version posted February 19, 2025. The copyright holder for this preprint (which was not certified by peer review) is the author/funder, who has granted bioRxiv a license to display the preprint in perpetuity. It is made available under aCC-BY 4.0 International license.

Metabolic cohort

Fig. S10

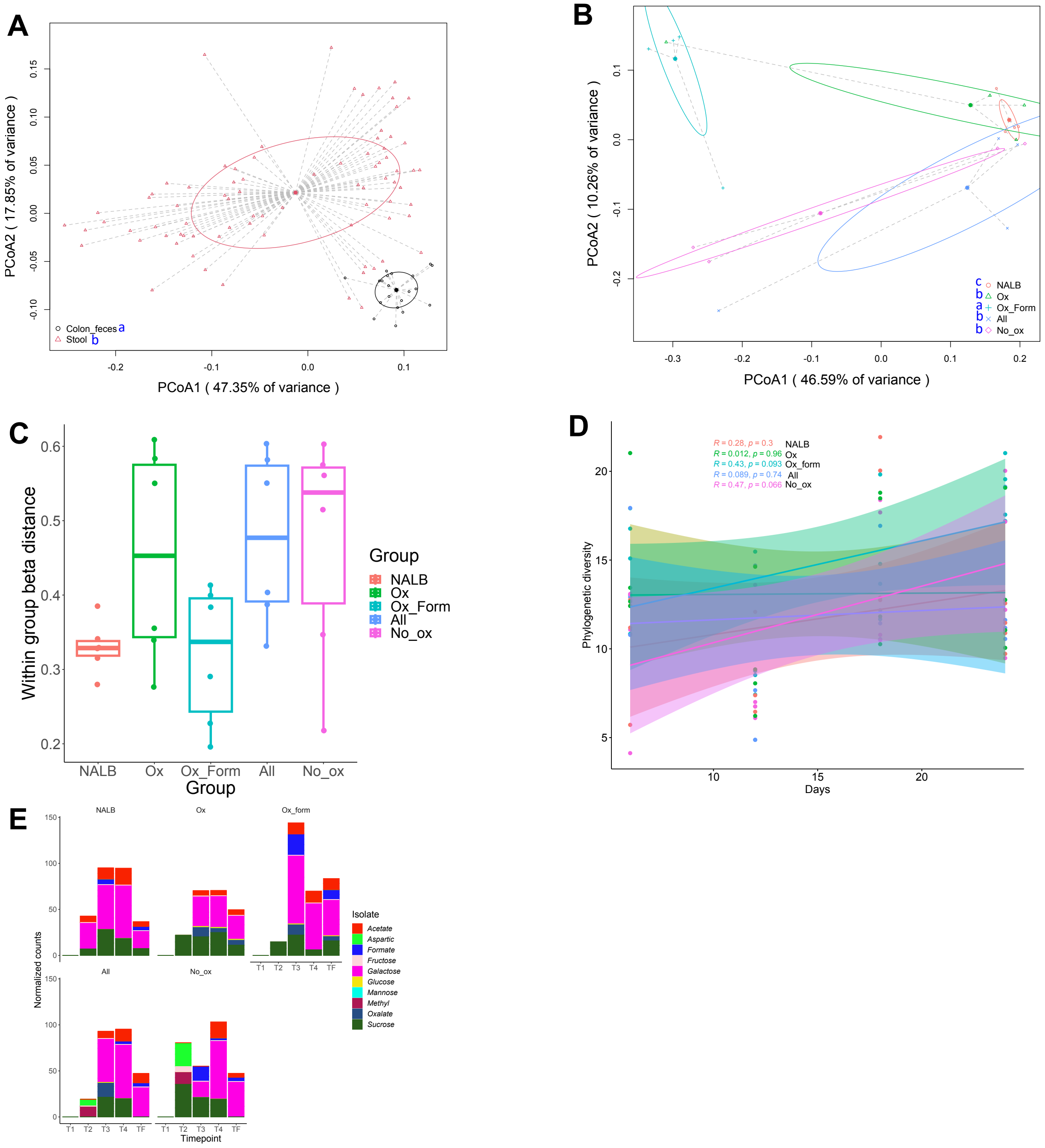

bioRxiv preprint doi: https://doi.org/10.1101/2024.10.28.620613; this version posted February 19, 2025. The copyright holder for this preprint (which was not certified by peer review) is the author/funder, who has granted bioRxiv a license to display the preprint in perpetuity. It is made available under aCC-BY 4.0 International license.

Fig. S11

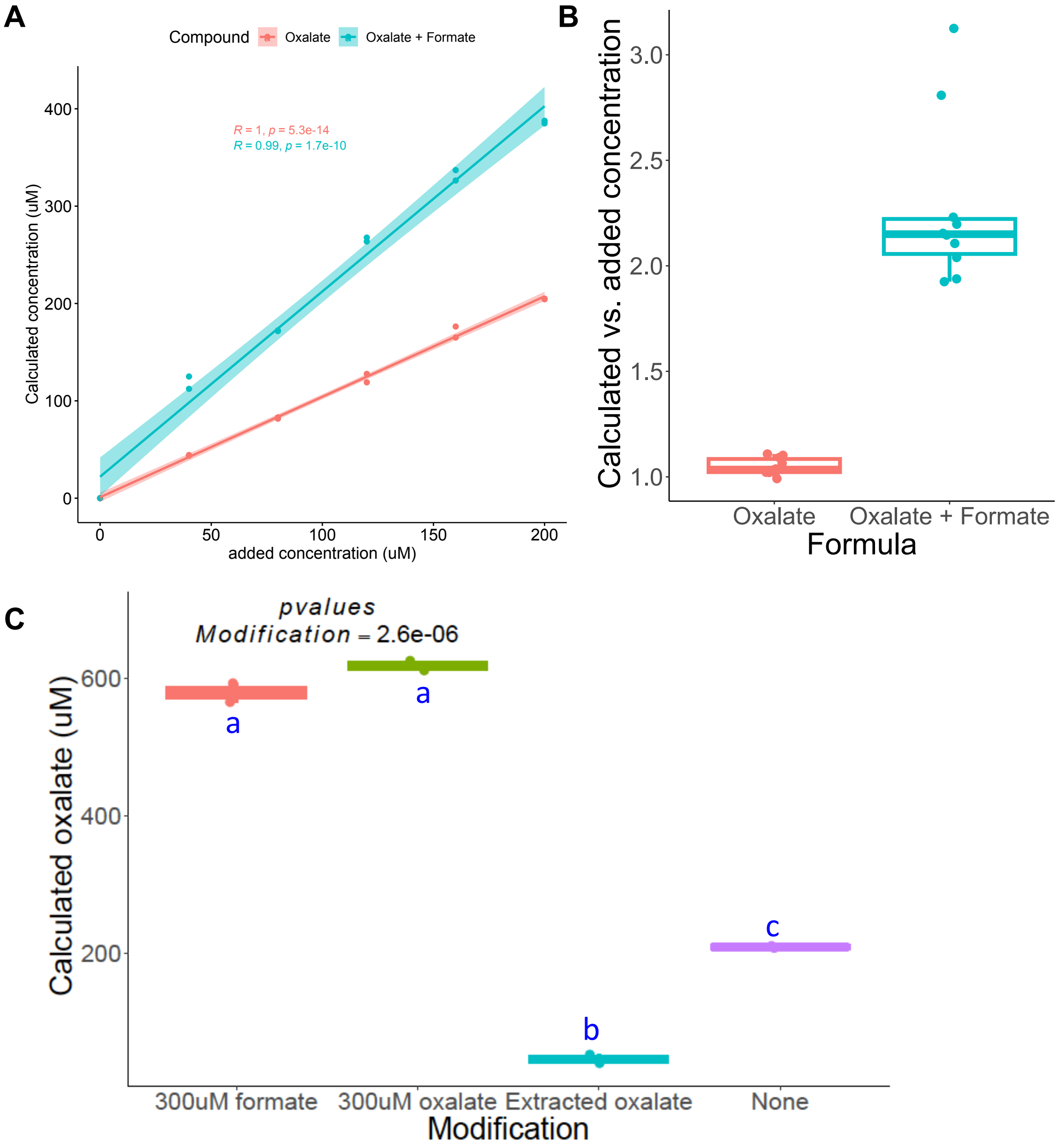

bioRxiv preprint doi: https://doi.org/10.1101/2024.10.28.620613; this version posted February 19, 2025. The copyright holder for this preprint (which was not certified by peer review) is the author/funder, who has granted bioRxiv a license to display the preprint in perpetuity. It is made available under aCC-BY 4.0 International license.

Supplementary Tables

Table S1.

**Ingredients**	**0% oxalate (g/Kg)**	**1.5% oxalate (g/Kg)**	**3% oxalate (g/Kg)**
Casein	200	200	200
L-Cysteine	3	3	3
Corn Starch	285.5	NA	NA
Maltodextrin	120	120	120
Sucrose	100	185.4	185.4
Anhydrous Milkfat	NA	200	200
Soybean Oil	70	70	70
Cellulose	150	150	150
Mineral Mix	42	42	42
Vitamin Mix	12	12	12
Choline Bitartrate	2.5	2.5	2.5
TBHQ, antioxidant	0.014	0.054	0.054
Sodium Oxalate	0	15	30

bioRxiv preprint doi: https://doi.org/10.1101/2024.10.28.620613; this version posted February 19, 2025. The copyright holder for this preprint (which was not certified by peer review) is the author/funder, who has granted bioRxiv a license to display the preprint in perpetuity. It is made available under aCC-BY 4.0 International license.

Table S6.

**Component**	**Amount/L**	**Concentration**	**Comments**
Tryptone Peptone	2 g	0.20%	
Yeast Extract	1 g	0.10%	
Meat Extract	5 g	0.50%	
KH_2PO4	100 mL	100 mM	1M stock solution pH 7.2
$NaHCO_3$	0.4 g	4.8 mM	
$NaCl_2$	0.08 g	1.37 mM	
CaCl2	1 mL	0.80%	0.8g/100mL stock
Vitamin K (menadione)	1 mL	5.8 mM	1 mg/mL stock solution
$FeSO_4$	1 mL	1.44 mM	0.4 mg $FeSO_4$/mL stock solution
Histidine Hematin Solution	1 mL	0.10%	1.2 mg hematin/mL in 0.2M histidine
Tween 80	2 mL	0.05%	25% stock solution
Aspartic acid	0.02g		
Mannose	0.9g		
Galactose	1.8g		
Sodium acetate	6g	0.60%	
Sodium formate	4g	0.40%	
Isovaleric acid	0.1 mL	1 mM	Must be added in fume hood. Keep media, gloves, and tips in fume hood after addition for at least 5 minutes.
Propionic acid	2 mL	8 mM	Must be added in fume hood. Keep media, gloves, and tips in fume hood after addition for at least 5 minutes.
Butyric acid	2 mL	4 mM	Must be added in fume hood. Keep media, gloves, and tips in fume hood after addition for at least 5 minutes.
Resazurin	4 mL	4 mM	0.25 mg/mL stock solution
Noble Agar	12 g	1.20%	Only add agar if making plates. If broth, omit.
pH to 7			
Add DI water to 910ml			
Autoclave			
Prepare glucose, cellobiose, maltose, fructose, sucrose, and methyl-x compounds as below			
Move media to anaerobic chamber and add remaining components			
D-glucose	0.4 g	2.2 mM	Add to 10ml DI H2O and filter sterilize
Cellobiose	1 g	2.9 mM	Add to 20ml DI H2O and filter sterilize
Maltose	1 g	2.8 mM	Add to 10ml DI H2O and filter sterilize
Fructose	1 g	2.2 mM	Add to 10ml DI H2O and filter sterilize
Sucrose	1.71g		Add to 10ml DI H2O and filter sterilize
Methylvaleric acid	561ul		Combine with methyl butyric and filter sterilize
Methylbutyric acid	517ul		
sodium sulfate	2g	0.20%	Add to 10ml DI H2O and filter sterilize in anaerobic chamber
L-cysteine	0.5 g	3.2 mM	Add to 10ml DI H2O and filter sterilize in anaerobic chamber
$MgSO_4$-$7H_20$	1ml	0.008 mM	Stock 0.2g/100ml & autoclave
ATCC Vitamin Mix	10 mL	1%	
ATCC Trace Mineral Mix	10 mL	1%	
**Let media sit in anaerobic chamber for at least 24hrs to be fully reduced before inoculation			

bioRxiv preprint doi: https://doi.org/10.1101/2024.10.28.620613; this version posted February 19, 2025. The copyright holder for this preprint (which was not certified by peer review) is the author/funder, who has granted bioRxiv a license to display the preprint in perpetuity. It is made available under aCC-BY 4.0 International license.

Table S7.

Component	Amount/L	Concentration	
KH_2PO4	100 mL	100 mM	
$NaHCO_3$	0.4 g	4.8 mM	
$NaCl_2$	0.08 g	1.37 mM	
CaCl2	1 mL	0.80%	0.8g/100mL stock; autoclave
Vitamin K (menadione)	1 mL	5.8 mM	1 mg/mL stock solution; protect from light; autoclave
$FeSO_4$	1 mL	1.44 mM	0.4 mg $FeSO_4$/mL stock solution; filter sterilize
Histidine Hematin Solution	1 mL	0.1%	1.2 mg hematin/mL in 0.2M histidine; filter sterilize after preparation of stock; protect from light; store at 4C
NH4Cl	1 g		
Tween 80	2 mL	0.05%	
$MgSO_4$-$7H_20$***	1 mL	0.008 mM	
ATCC Vitamin Mix***	10 mL	1%	
ATCC Trace Mineral Mix***	10 mL	1%	
For sole C&E media, add one of the following to Media A			
Aspartic acid	0.02g		
Mannose	0.9g		
Galactose	1.8g		
Sodium acetate	6g	0.60%	
Sodium formate	4g	0.40%	Requires sodium sulfate
D-glucose	0.4 g	2.2 mM	Add to 10ml DI H2O and filter sterilize
Fructose	1 g	2.2 mM	Add to 10ml DI H2O and filter sterilize
Sucrose	1.71g		Add to 10ml DI H2O and filter sterilize
Methylvaleric acid	561ul		Add both methylvaeric and methylbutyric
Methylbutyric acid	517ul		
Oxalate	6.75g	50mM	
pH to 7			
Filter sterilize and bring into anaerobic chamber			
Cysteine	0.5g		Add to 10ml DI H2O and filter sterilize in anaerobic chamber
sodium sulfate	2g	0.20%	Only add if adding sodium formate

bioRxiv preprint doi: https://doi.org/10.1101/2024.10.28.620613; this version posted February 19, 2025. The copyright holder for this preprint (which was not certified by peer review) is the author/funder, who has granted bioRxiv a license to display the preprint in perpetuity. It is made available under aCC-BY 4.0 International license.

Table S8.

**Species**	**Commercial source**	**Isolation source**
Oxalobacter formgienes	ATCC 35274	human
Desulfovibrio piger	DSMZ749	Sheep rumen
Methanobrevibacter smithii	DSMZ11975	animal feces
Ruminococcus gnavus	ATCC 29149	human
Coprococcus comes	ATCC 27758	human
Bacteroides thetaiotaomicron	ATCC 700349	human
Lactobacillus helveticus	ATCC 55796	Unknown
Lactobacillus plantarum	ATCC 700210	Unknown
