# Supplementary material for: Complex system modelling reveals oxalate homeostasis is driven by diverse oxalate-degrading bacteria
Source: bioRxiv. 2024 Oct 28:2024.10.28.620613. Preprint. [Version 1] doi: 10.1101/2024.10.28.620613 (PMC11565779; doi:10.1101/2024.10.28.620613)
Supplement: Supplementary file 1 [file NIHPP2024.10.28.620613v1-supplement-1.pdf]

bioRxiv preprint doi: https://doi.org/10.1101/2024.10.28.620613; this version posted October 28, 2024. The copyright holder for this preprint (which was not certified by peer review) is the author/funder, who has granted bioRxiv a license to display the preprint in perpetuity. It is made available under aCC-BY 4.0 International license.

Supplemental information titles and legends

STAR Methods
Supplementary Figures and table legends
Figs. S1 to S10
Tables S1 and S8
References (*1-56*)

Star Methods

Animal studies.

Tracking animal health

Throughout all diet trials, animals were monitored for evidence of trauma, dehydration, pain, or other forms of suffering. Additionally, water and food intake, urine output, and body mass was monitored daily (i.e., Figs. S6, S9). If animals lost >10% of their baseline body mass, they were removed from the diet trial and placed back onto standard mouse chow.

Effect of oxalate on the native gut microbiota of Neotoma albigula

To determine the impact of dietary oxalate on *N. albigula* with their native microbiota, 14 adult *N. albigula* woodrats were collected from Castle Valley, UT (38.63′N, 109.41′W) in September 2014, using Sherman live traps. Animals were transported to the University of Utah and housed in individual cages (48 by 27 by 20 cm) with a 12-h/12-h light/dark cycle, at 28°C and 20% humidity. Animals were initially maintained on a 0.2% oxalate, high-fiber rabbit chow (Harlan Teklad formula 2031; Envigo) for 7 months prior to experimentation. This diet reduces the overall detectable diversity of the microbiota of these animals but maintains the members of the native microbiota overall [1]. All methods were approved by the IACUC under protocol no. 12-12010.

Subsequently, *N. albigula* woodrats were placed in a diet trial in which the oxalate concentration of the food was gradually increased over time along a gradient from 0.2% (for days 1 to 5; herein referred to as 0%), 3% (days 6 to 10), and 6% (days 11 to 15; Fig. 3A). The oxalate concentration of the diet was adjusted by adding the appropriate amount of sodium oxalate (Fisher Scientific, Pittsburgh, PA) into the powdered rabbit chow on a dry weight basis. The schedule for each concentration of dietary oxalate was chosen to ensure that the gut microbiota had time to respond to the specific diet [2,3]. During diet trials, oxalate did not have a negative impact on *N. albigula* health and neither fecal nor urinary oxalate levels increased significantly[4].

During the diet trial, animals were placed in metabolic cages to separate urine and feces into sterile 50-ml conical tubes. A subsample of feces was collected from each animal every 4 to 5 days on the 0% and 6% oxalate diets for shotgun metagenomic sequencing. Samples were frozen at −80°C until DNA extraction.

Stool collection for bacterial isolations and fecal transplant studies

bioRxiv preprint doi: https://doi.org/10.1101/2024.10.28.620613; this version posted October 28, 2024. The copyright holder for this preprint (which was not certified by peer review) is the author/funder, who has granted bioRxiv a license to display the preprint in perpetuity. It is made available under aCC-BY 4.0 International license.

Feces for fecal transplants were collected from two sources. For the Swiss-Webster feces, 20 individual animals (Taconic Farms, female, six weeks old) were placed on a 0% oxalate diet in cages with a custom-designed insert to separate urine and feces at the Cleveland Clinic (IACUC #2016-1653) [5]. For the *N. albigula* feces, 14 individual animals (mixed sex and age) were placed on a 3% oxalate diet in a metabolic chamber at the University of Utah (IACUC #12-12010) to acclimate the microbiome to oxalate metabolism[5]. After 3 days of acclimation on the respective diets, feces were collected from animals within 2 h of defecation, submerged in sterile 15% glycerol and flushed with CO_2 prior to freezing at −80 °C until use in animal studies approximately 12 months after receipt (IACUC #2016-1653) or for bacterial isolations.

Microbiota-dependent effects of oxalate on liver and gut microbiota

All animal studies were approved through the Cleveland Clinic's IACUC (IACUC #'s 2016-1653, 2020-2312). To examine the host-independent effects of oxalate on gut microbiota with either a low or high efficiency at degrading oxalate, two distinct host-microbe model systems were developed. For both models, equal numbers of male and female Swiss-Webster mice were used as the host. For the low oxalate-degrading gut microbiota, Swiss-Webster mice were given fecal transplants using feces from other Swiss-Webster mice (allografts), as discussed below. A 1.5% oxalate diet is sufficient to induce hyperoxaluria in Swiss-Webster mice, defined as a 50% increase in urinary oxalate excretion[6-8]. For the high oxalate-degrading gut microbiota, Swiss-Webster mice were given fecal transplants from *N. albigula* (xenografts). We have previously shown that fecal transplants from *N. albigula* are sufficient to induce significant and persistent oxalate metabolism[7,8]. During and after fecal transplants, animals were grouped four to a cage, with four cages assigned to each treatment group, for a total of 16 animals per experimental group. To minimize individual variability and eliminate any cage effect in subsequent metrics, samples and data collected from all animals in a cage were pooled together into an individual sample. To develop the two different mouse models, the conversion of the gut microbiota from the native microbiota to the grafted microbiota (allografts or xenografts) was completed in two stages. First, the native microbiota was depleted with a five-day course of neomycin (0.5g/L water, combined with 2g/L sucralose), while on a 0% oxalate diet. Mice were given *ad libitum* access to water during this time and throughout the experiment. Neomycin is a broad spectrum antibiotic that is poorly absorbed across the gut, and effectively reduces gut microbiota density by up to 90%[9]. After antibiotic depletion, mice were given the respective fecal transplants by first thawing feces, then aseptically mixing 32 g feces per kg body weight directly into powdered mouse chow. Fecal transplants were performed daily for 6 days (Fig. 1A), as previously described[5], during which animals were switched to either a 0% or 1.5% oxalate diet. All animals were maintained on their respective diets for two weeks following fecal transplants (Table S1). A total of four treatment groups were defined based on allografts/xenografts and oxalate diet.

Following diet trials, animals were sacrificed through CO_2 asphyxiation and cervical dislocation. Upon necropsies, liver tissue from each animal was dissected out and placed into RNAlater on ice, prior to freezing at -80ºC, within two hours of necropsies.

bioRxiv preprint doi: https://doi.org/10.1101/2024.10.28.620613; this version posted October 28, 2024. The copyright holder for this preprint (which was not certified by peer review) is the author/funder, who has granted bioRxiv a license to display the preprint in perpetuity. It is made available under aCC-BY 4.0 International license.

Additionally, colon feces were collected and placed into screw-cap tubes with o-rings, on ice, prior to freezing at -80ºC.

Microbial cohort-dependent effects on mitigating the systemic effects of oxalate on host and microbiome

To determine the effect of specific microbial cohorts at mitigating the systemic host-microbiome effects of oxalate, different combinations of two microbial cohorts were established. First, based on the metabolic pathways enriched by oxalate in the *N. albigula* gut microbiota (Fig. 3D,5C, Table S5), assessed through comparative shotgun metagenomics (described below), we hypothesized that microorganisms that engage in those metabolic pathways help to mitigate the systemic effects of oxalate. To test this hypothesis, we isolated bacteria from *N. albigula* stool (described below) and grouped microorganisms into four cohorts (Fig. 5D). These included oxalate-degrading bacteria alone (Ox; Group 2), microorganisms that can utilize formate or oxalate (Ox_form; Group 3), oxalate and formate users, in addition to the metabolism of selected sugars (All; Group 4), or the complete cohort except for oxalate degraders (No_ox; Group 5). Microorganisms isolated from *N. albigula* stool and used in microbial transplants were termed the "metabolic cohort". In addition to the metabolic cohort, a "taxonomic cohort" was defined by cross-referenced metabolic pathways enriched by oxalate with microbial taxa previously reported to be stimulated by oxalate (Fig. 5D)[10]. Microorganisms from this cohort were purchased commercially from the ATCC or DSMZ (Table S8). In addition to these cohorts, we also examined a negative control in which animals were inoculated with sterile media (No_bact (Group 1); Group 1), and a positive control in which animals were inoculated with the whole gut microbiota of *N. albigula,* extracted from stool by first vortexing in a 1:1 mixture of stool and sterile PBS, followed by centrifuging at 8000RPM for 2 minutes. The supernatant was inoculated into gut microbiota media with 20mM sodium oxalate (Table S6) overnight prior to microbial transplant.

To prepare each isolate for transplants, an overnight culture of each species was grown and mixed in equal proportions by volume for each cohort. The community was then centrifuged and supernatant was decanted. After antibiotic depletion of the native gut microbiota in Swiss-Webster mice, 38mg of pelleted bacteria of each preparation was added directly to the food and administered over the course of six days (Figs. 6, S8), as done previously[6,7]. Preparations corresponded to approximately 5 x 10^8 microorganisms for each isolate in the appropriate transplant groups. Quantification of bacterial numbers was performed through absorbance of the microbial preparations on a spectrophotometer at 600 nm, calibrated to direct microscopic counts.

As above, animals were grouped four to a cage, with samples from each cage pooled together prior to collection and analyses. Similarly, animals were maintained on custom inserts that mimic metabolic cage for the effective separation of urine and stool prior to collection. During the microbial transplant animal studies, stool and urine was collected daily and inserts disinfected. For oxalate and formate quantification, samples were collected at the end of the antibiotic depletion, microbial transplants, a 0% oxalate washout period, and after return to a 3% oxalate diet (Figs. 6B-D, S8B-D). Prior to

bioRxiv preprint doi: https://doi.org/10.1101/2024.10.28.620613; this version posted October 28, 2024. The copyright holder for this preprint (which was not certified by peer review) is the author/funder, who has granted bioRxiv a license to display the preprint in perpetuity. It is made available under aCC-BY 4.0 International license.

biochemical assays, urine was frozen (−20°C) and feces were dried at 45°C overnight. Prior to freezing, aliquots of urine were equally divided into six fractions for each of the biochemical assays performed (discussed below). Fractions for oxalate quantification were collected into 4N HCl prior to freezing to prevent the non-enzymatic conversion of ascorbic acid into oxalate [2]. For microbial inventories, a portion of feces and colon feces (discussed below) were frozen at −80°C. All animals were maintained on a 3% oxalate diet, except during the washout period (Table S1).

Following diet trials, animals were sacrificed through CO_2 asphyxiation and cervical dislocation. Necropsies were performed to aseptically remove colon feces, colon, kidneys, and heart tissue. Colon tissue was longitudinally bisected, swiss-rolled, and placed into 4% paraformaldehyde overnight for fixation at 4ºC for histopathology [11]. Kidney and heart tissues were placed into 4% paraformaldehyde overnight for fixation at 4ºC prior to histopathology. Hearts were first perfused with paraformaldehyde solution prior to immersion.

After fixation, colon, kidney, and heart tissue were placed in 70% ethanol at 4ºC prior to paraffin embedding, serial sectioning, and staining. To quantify colitis, colon tissue was stained with hematoxylin and eosin and imaged with light microscopy (Fig. 6H). Whole colons were scored for colitis by two blinded, independent reviewers using a semi-quantitative protocol that considers inflammatory infiltrates, goblet cell loss, crypt density, crypt hyperplasia, muscle thickening, submucosal inflammation, crypt abcesses, and ulcerations[12]. To quantify calcium oxalate deposition, kidney tissue was stained with Von Kossa staining[13], which turns calcium deposits black and the remaining tissue pink (Figs. 6E, S8E). Kidneys were imaged under light microscopy and calcium oxalate deposition was quantified using an automated process in QuPath, based on stain color. The area of the black calcium oxalate deposits was normalized to total kidney area. To quantify cardiac fibrosis, heart tissue was stained with Masson's trichrome stain, which stains collagen fibrosis blue and the remaining tissue red (Fig. S6H). Hearts were imaged under light microscopy and fibrosis was quantified with an automated process in QuPath, quantifying the blue and red stained areas[14]. Fibrosis was normalized to total heart surface area.

Biochemical assays.
Urinary creatinine (Fisher Scientific), formate (VWR), IL-6 (R&D Systems), IL-18 (Fisher Scientific), and IL-1β (R&D Systems) were quantified with ELISA-based assays, following manufacturer's instructions. Positive controls that included a known amount of substrate along with no substrate negative controls were included in all batches of ELISA assays. Samples, standards and controls were all run in duplicate and values averaged. Urinary and fecal oxalate were quantified with an ELISA-based assay (Sigma-Aldrich), with a modified protocol, as follows. Upon initial testing of oxalate and formate assays using solutions of sodium oxalate and sodium formate in water, it was discovered that while formate assays only had affinity for formate, the oxalate assays had equal affinity for both oxalate and formate (Figs. S11A). Specifically, when oxalate alone was added to solution, the quantified concentration of oxalate matched the amount added. However, when oxalate and formate was added at equal

bioRxiv preprint doi: https://doi.org/10.1101/2024.10.28.620613; this version posted October 28, 2024. The copyright holder for this preprint (which was not certified by peer review) is the author/funder, who has granted bioRxiv a license to display the preprint in perpetuity. It is made available under aCC-BY 4.0 International license.

concentrations, the quantified oxalate was approximately twice as high as what was added (Fig. S11B). Furthermore, when adding 300uM of formate or oxalate to human urine specimens, the amount of oxalate quantified was approximately 300uM higher than urine without any modifications (Fig. S11C). To eliminate the formate contamination on oxalate measurements, oxalate was extracted by first acidifying to pH 3 with 3M HNO_3. Acidified urine was centrifuged, and supernatant collected. The pH was then brought up to 7 with NaOH. Subsequently, 5M $CaCl_2$ was added to solution to precipitate calcium oxalate. The precipitates were extracted by centrifugation and decanting. Finally, calcium oxalate solution was acidified in 1:4 parts of 4N HCl prior to enzymatic assay, as previously described[2]. When oxalate was extracted from urine specimens in this way, prior to quantification, we saw a significant decrease in quantified oxalate (Fig. S11C), as expected given the assay affinity for formate. When solutions containing formate alone went through the extraction process, no oxalate was quantified. Given these results, urinary and fecal oxalate was extracted prior to ELISA-based assays using the manufacturer's recommendations, post-extraction. For fecal oxalate, samples were acidified with 6N H_2SO_4 to solubilize oxalate prior to following the extraction protocol above. For creatinine, cytokines, and oxalate, values generated on a 0% oxalate diet were substracted from all other values, matched by cage, with the assumption that this would isolate oxalate-induced molecule generation. For urinary formate, this substraction was not done since formate can come from multiple sources other than oxalate degradation and values on a 0% oxalate diet were not clearly lower than on a 3% oxalate diet. For all values, data are presented as the change from baseline. Data were statistically compared with two-way analyses and post-hoc, Holm's-corrected, paired t-tests.

Culturomic assays.

All assays were conducted under strict anaerobic conditions (90% N_2, 5% H_2, 5% CO_2) in an anaerobic chamber (Bactron 300).

The stool from *N. albigula* were used to determine the impact of environmental factors on oxalate metabolism *in vitro* and to isolate bacteria for the metabolic cohort. *Enterococcus gallinarum,* an oxalate-degrading species previously isolated from *N. albigula,* was also used in culturomic assays to determine the impact of environmental factors on oxalate metabolism[15]. To test environmental factors on oxalate metabolism, gut microbiota media, previously designed to support a broad array of gut bacteria[16], was used as a base media. From there, compounds were added to the media based on metabolic pathways enriched by dietary oxalate in the *N. albigula* gut microbiota (Fig. 3D, Table S6). Concentrations of added chemicals were based on previously published media recipes where necessary. To assess oxalate metabolism in different culture conditions, oxalate was quantified by extracting oxalate and quantification through titration with $KMnO_4$, as we have done previously[15].

To isolate bacteria for the metabolic cohort and to validate the metabolic redundancy of oxalate metabolism and other pathways stimulated by oxalate, a minimal media with the same substrates from above were added as sole carbon and energy sources (Table S7). In this medium, oxalate was added at 50mM to specifically select for bacteria that

bioRxiv preprint doi: https://doi.org/10.1101/2024.10.28.620613; this version posted October 28, 2024. The copyright holder for this preprint (which was not certified by peer review) is the author/funder, who has granted bioRxiv a license to display the preprint in perpetuity. It is made available under aCC-BY 4.0 International license.

can use oxalate as a carbon and energy source. Subsequently, the NALB microbiota from the stool was first diluted to ~1 bacterium/100ul and then inoculated into 5 or 10 96-well plates, depending on the rarity of positive hits, and quantifying growth through spectrophotometry. Wells in which the absorbance values at 600nm for greater than one standard deviation above the average for all well were considered positive for growth. Wells with positive growth had bacteria isolated by streaking on rich media, transferred to broth cultures, and had stocks made in 15% glycerol that were frozen at -80ºC until needed. Those isolates grown in oxalate or formate containing media were subsequently enriched in the minimal media and oxalate or formate degradation was validated through broth cultures and ELISA-based assays.

Metabolomics.
For untargeted metabolomic assays, fecal samples were re-suspended in Optima LC/MS grade water (50mg stool to 150ul water. The samples were then placed briefly in a water bath at 37°C and placed on dry ice. An aliquot of each sample was taken out for protein concentration measurement. Then, 150 µL of chilled acetonitrile containing internal standards (Betaine-d9, Carnitine-d9, Orinithine-d6, Valine-13C3, Tyrosine-13C, 15N, Estrone-13C3, and Cholesterol-13C3) was added to the remaining samples followed by centrifugation at 14,000g for 10 minutes to precipitate out the protein pellet. The supernatants were recovered and transferred to fresh tubes. The samples were dried briefly and re-suspended in 2% acetonitrile and 0.1% formic acid for subsequent LCMS analysis. One-microliter aliquots taken from each sample were pooled and this QC standard was analyzed every 10 injections. The untargeted metabolomics was performed by injecting 0.5uL of each sample onto a 10 cm C18 column (Thermo Fisher CA) coupled to a Vanquish UHPLC running at 0.35mL/min using water and 0.1% formic acid as solvent A and acetonitrile and 0.1% formic acid as solvent B. The 15-min gradient used is given below. The Orbitrap Q Exactive HF was operated in positive and negative electrospray ionization modes in different LC-MS runs over a mass range of 50-750 Da using full MS at 120,000 resolution. The data dependent acquisitions were obtained on the pooled QC sample. The DDA acquisition (DDA) include MS full scans at a resolution of 120,000 and HCD MS/MS scans taken on the top 10 most abundant ions at a resolution of 30,000 with dynamic exclusion of 4.0 seconds and the apex trigger set at 2.0 to 4.0 seconds. The resolution of the MS2 scans were taken at a stepped NCE energy of 20.0, 30.0 and 45.0.

XCMS was used to deconvolute the data using 5 ppm consecutive scan error, 7 to 75 seconds as minimum and maximum peak width, S/N threshold of 10, and span of 0.2 in positive and negative electrospray ionization modes for retention time correction. The resulting peak table was normalized to total protein concentration and further analyzed via MetaboLyzer [17]. Briefly, the ion presence threshold was set at 0.7 in each study group. Data were then log-transformed and analyzed for statistical significance via non-parametric Mann-Whitney U test. Ions present in just a subset of samples were to be analyzed as categorical variables for presence status via Fisher's exact test. All p-values were set to be corrected via the Benjamini-Hochberg step-up procedure for false discovery rate (FDR < 0.05). The data could then be utilized for PCA, putative identification assignment, and pathway enrichment analysis via KEGG[18], HMDB[19],

bioRxiv preprint doi: https://doi.org/10.1101/2024.10.28.620613; this version posted October 28, 2024. The copyright holder for this preprint (which was not certified by peer review) is the author/funder, who has granted bioRxiv a license to display the preprint in perpetuity. It is made available under aCC-BY 4.0 International license.

LIPIDMAPS[20], and BioCyc[21] databases. In addition to differential abundance analysis provided by MetaboLyzer, whole metabolome comparisons were made as a Binomial dissimilarity matrix, which is optimal for metabolomic datasets[22], with statistical analysis provided by PERMANOVA with 999 permutations and a principal component visualization in Vegan[23].

Transcriptomics.
For transcriptomic analysis, RNA was extracted from liver tissue with a TRIzol RNA purification kit (Invitrogen), following manufacturer's recommendations. Extracted RNA was converted to cDNA using a cDNA synthesis kit (Fisher Scientific) and stored at -20ºC. The cDNA quality was checked on a bioanalyzer and libraries were prepared with the TruSeq stranded mRNA library prep kit. Sequencing occurred on an Illumina HiSeq 2500 at the Genomics Core (Cleveland Clinic) for paired 100bp sequencing.

The raw sequencing files, with 33.5 million +/- 950,000 reads per sample, were pre-processed using an established protocol[24]. Specifically, FastQC and SortMeRNA was used to remove low quality reads, remove rRNA reads, and merge paired reads[25,26]. Subsequently, trimmomatic was used to remove adapter sequences[27] and the remaining high quality reads were mapped to the mm10 mouse reference genome for annotation, using HISAT[28,29]. The annotated gene counts were normalized to fragments per kilobase of transcript per million mapped reads. Comparative whole transcriptome analyses were conducted as a Bray-Curtis dissimilarity index and principal components visualization with statistical analysis provided by PERMANOVA with 999 permutations, in the Vegan package of R statistical software[23,30]. Differential abundance analysis was conducted in Cuffdiff2, with significance defined at a false discovery rate <0.05 [31]. Sequences mapping to mouse genes were annotated to metabolic pathway with the gene ontology database[32].

To integrate transcriptomic and metabolomic data, the normalized metabolite concentrations and normalized transcript counts of metabolites and genes that were significantly different between the SWM-SWM and SWM-NALB groups were correlated, within each group, using SPARCC correlations in R[33]. Significant positive and negative correlations were calculated at an R greater or less than 0.4 or -0.4, respectively and an FDR <0.05. Significant correlations were visualized in Cytoscape version 3.3.0[34].

Shotgun metagenomics.
For shotgun metagenomic analysis, DNA from *N. albigula* stool was extracted with the QIAmp DNA stool minikit (Qiagen). The extracted DNA was submitted to Argonne National Laboratory (Chicago, IL) for 150bp paired-end shotgun metagenomic sequencing on an Illumina HiSeq 2500. The low quality reads were trimmed from raw sequencing data and paired ends were merged using default parameters in BBMerge[35]. Reads were first mapped to the *Neotoma* genome [36] with BWA mem with default settings[37], and removed. Paired, non-rodent sequences were assembled in MetaSpades with default parameters[38].

bioRxiv preprint doi: https://doi.org/10.1101/2024.10.28.620613; this version posted October 28, 2024. The copyright holder for this preprint (which was not certified by peer review) is the author/funder, who has granted bioRxiv a license to display the preprint in perpetuity. It is made available under aCC-BY 4.0 International license.

Full-length microbial genes were extracted and annotated from the assembled contigs using PROKKA software[39]. Gene annotation was achieved by mapping to the UniProt and Hamap protein databases[40,41]. To dereplicate genes, ensure common nomenclature across samples, and build a reference gene catalog for the quantification of gene counts, full-length genes were clustered at 90% homology in CD-hit[42]. After the creation of a non-redundant gene catalogue, high quality reads for each sample were mapped to the annotated gene catalog to generate gene-level count tables with BWA mem.

Gene count tables were normalized and differential abundance analysis was conducted with a negative binomial Wald test in DESeq2[43]. Significantly different genes were considered at an FDR <0.05. The normalized count tables were also used to generate a weighted Bray–Curtis dissimilarity matrix in the Vegan R package, with statistical comparisons with a PERMANOVA at 999 permutations[23]. To annotate differentially abundant genes to taxonomy, genes were mapped to full length genomes extracted directly from the shotgun metagenomic data, as described below.

de novo *genome construction and calculation of metabolic redundancy*

For the *de novo* construction of genomes, the metaspades assembled contigs were binned to genomes with Autometa software[44], as previously described[45]. The Autometa algorithm takes assembled contigs as input, bins the data to genomes using contig coverage values, GC content, and the Barnes-Hut t-Distributed Stochastic Neighbor Embedding (BH t-SNE) distribution of the contigs. Taxonomy is assigned based on the consensus classification of all contigs in a genomic bin. Completeness and purity calculations are based on the presence of known, unique universal single copy genes in Autometa. After genomic binning, the contigs were oriented and scaffolded in CSAR[46], which compares the genomic bins to reference genomes of close relatives. Gaps within the genomes were filled with Abyss-Sealer[47] using the original raw sequencing reads as input. The completeness and purity of genomes were recalculated and taxonomic assignment was validated through phylogenetic analysis of the *de novo* constructed genomes in comparison with all complete NCBI genomes (Fig S1), achieved through Phylophlan, using the "—accurate" parameter which considers more phylogenetic positions at the cost of computational speed[48]. Phylophlan allows for the phylogenetic analysis of bacteria and archaea using complete genomes rather than gene amplicons, which provides greater resolution on phylogenetic analysis. Collectively, we extracted 248 high quality, full length genomes, defined as being >80% complete and >90% pure, based on current standards[49].

To quantify the proportion of the *N. albigula* microbiota that contained genes related to oxalate metabolism, formate metabolism, acetogenesis, methanogenesis, or sulfate reduction, all genes that contribute to those metabolic pathways were extracted from the KEGG database[50]. Subsequently, PROKKA[39] was run on the 248 full length genomes to extract and annotate full length genes. The PROKKA extracted genes were then cross-referenced with the gene databases from KEGG to determine the proportion of genomes that contained at least one gene in the target pathways and determine which taxa had those genes.

bioRxiv preprint doi: https://doi.org/10.1101/2024.10.28.620613; this version posted October 28, 2024. The copyright holder for this preprint (which was not certified by peer review) is the author/funder, who has granted bioRxiv a license to display the preprint in perpetuity. It is made available under aCC-BY 4.0 International license.

16S rRNA metagenomics.
Fecal DNA from mice in the SWM-NALB and SWM-SWM studies, underwent DNA extraction through a semi-automated protocol on a KingFisher Duo Prime System (Thermo Scientific) following the manufacturer's protocol for stool. The protocol includes mechanical lysis with bead-beating, piston driven lysis, and proteinase K chemical lysis. The extracted DNA, which attaches to magnetic beads in a proprietary salt solution, is removed from solution and eluted in buffer. Duplicate samples of a commercial DNA positive of known composition (Zymboiomcs, USA) were included as positive controls, as well as the DNA reagents that went through the entire workflow, sterile water, and a PCR negative as negative controls.

The fecal DNA was submitted to the Microbial Sequencing and Analytics Core at Cleveland Clinic for high throughput sequencing of the 16S rRNA gene on an Illumina MiSeq. The DNA was first PCR amplified with the 515F and 806R primers that target the V4 hypervariable region of the 16S gene. The DNA concentrations were quantified before and after PCR amplification on a Qubit and normalized prior to library prep with the Illumina Nextera XT library prep kit. The sequence run was conducted to generate 150bp, paired-end sequences.

The raw sequencing data were processed in the R statistical package (4.2.0)[30]. Quality control, removal of bimeras, and assignment of amplicon sequence variants (ASV) were completed in dada2[51]. For ASV taxonomic assignment, a combined, non-redundant database of the Silva 138 SSURef and NCBI 16S rRNA databases were used[52]. Taxa assigned to mitochondria or chloroplasts were removed from subsequent analysis. The resulting ASV's were aligned in MSA[53] and arranged into a maximum likelihood phylogeny in phangorn[54]. The resulting phylogenetic tree and ASV table were merged with sample data for loading into PhyloSeq[55]. The sequencing depth threshold required to adequately capture microbial diversity was calculated with a rarefaction analysis in Vegan[23]. Non-control samples below the depth threshold were removed from further analysis.

The raw count table of 16S rRNA sequences was normalized with the DESeq2 algorithm and α-diversity was calculated as phylogenetic diversity in Phyloseq, along with beta-diversity as a weighted UniFrac distance[56]. Alpha diversity was analyzed with a paired t-test with a Holm's correction where applicable, while beta-diversity analyses were conducted with a PERMANOVA after 999 permutations.

Supplementary figure legends:

Fig. S1. Phylogeny of genomes extracted from the *N. albigula* metagenome, in comparison to >3000 full length microbial genomes. Stars indicate taxonomic placement of extracted genomes. All other points indicate reference genomes.

bioRxiv preprint doi: https://doi.org/10.1101/2024.10.28.620613; this version posted October 28, 2024. The copyright holder for this preprint (which was not certified by peer review) is the author/funder, who has granted bioRxiv a license to display the preprint in perpetuity. It is made available under aCC-BY 4.0 International license.

Fig. S2. Representation of formate metabolism genes in the NALB metagenome derived from 248 full length genomes. A) Proportion of the genomes extracted from *N. albigula* that had at least one formate metabolism gene. B) Representation of formate metabolism genes among the metagenome, broken down by gene function and proportion of total formate metabolism genes. C) Representation of formate metabolism genes broken down by taxon.

Fig. S3. Representation of acetogenic genes in the NALB metagenome derived from 248 full length genomes. A) Proportion of the genomes extracted from *N. albigula* that had at least one acetogenic gene. B) Representation of acetogenic genes among the metagenome, broken down by gene function and proportion of total acetogenic genes. C) Representation of acetogenic genes broken down by taxon.

Fig. S4. Representation of methanogenic genes in the NALB metagenome derived from 248 full length genomes. A) Proportion of the genomes extracted from *N. albigula* that had at least one methanogenic gene. B) Representation of methanogenic genes among the metagenome, broken down by gene function and proportion of total methanogenic genes. C) Representation of methanogenic genes broken down by taxon.

Fig. S5. Representation of sulfate-reducing genes in the NALB metagenome derived from 248 full length genomes. A) Proportion of the genomes extracted from *N. albigula* that had at least one sulfate-reducing gene. B) Representation of sulfate-reducing genes among the metagenome, broken down by gene function and proportion of total sulfate-reducing genes. C) Representation of sulfate-reducing genes broken down by taxon.

Fig. S6. Microbial transplant composition impact on urinary inflammation, renal health, and overall mouse health for the taxonomic cohort. A-G) The effect of microbial transplants on urinary IL-6 (A), IL-18 (B), and creatinine (C) levels, along with water intake (D), urine output (E), food intake (F) and body mass (G). H) Masson's Trichrome staining of heart tissue reveals fibrosis. Shown are representative images from the No_bact (Group 1) and All (Group 4) groups. I) Quantification of cardiac fibrosis from different microbial transplant groups. Statistical significance - 2-way ANOVA (shown on charts) and post-hoc, Holm's corrected, paired t-tests. Blue letters reflect statistical groups between microbial transplants and *$p<0.05$, **$p<0.01$; ***$p<0.001$ for longitudinal comparisons within each microbial transplant group.

Fig. S7. Effect of microbial transplants on microbial community composition for the taxonomic cohort. A,B) Beta-diversity analysis based on a weighted UniFrac dissimilarity matrix of colon feces collected at the end of the study period vs. stool (A; . $p=0.006$, PERMANOVA) or in the endpoint stool samples by group (B; no significant differences). Similar results for (B) were obtained with colon feces. Statistical differences shown by blue letters in legend. C) Within group beta-diversity distance of colon feces. $p=0.006$, one-way ANOVA. Blue letters represent statistical groups, based on post-hoc, Holm's corrected, paired t-tests. Similar results were found with endpoint

bioRxiv preprint doi: https://doi.org/10.1101/2024.10.28.620613; this version posted October 28, 2024. The copyright holder for this preprint (which was not certified by peer review) is the author/funder, who has granted bioRxiv a license to display the preprint in perpetuity. It is made available under aCC-BY 4.0 International license.

stool samples. D) Change in phylogenetic diversity of stool for each microbial transplant group. Stats, shown on graph, are based on Pearson correlations. E) Normalized counts of transplanted microorganisms in the stool across timepoints and in the colon feces at the end of the diet trial.

Fig. S8. Microbial community composition (metabolic cohort) impacted the effect of exogenous oxalate on kidney health. A) Experimental design. Swiss Webster mice were given neomycin, followed by inoculation of one of five microbial consortia that included either no bacteria, the NALB community, or the metabolic cohort listed in Figure 5C. Animals were maintained on a 3% oxalate diet throughout the trial except for a 0% oxalate washout period after the microbial transplant period. B,C) The effect of microbial transplants on urinary (B) or fecal (C) oxalate levels over the course of the diet trial, compared to baseline. ANOVA $p<0.001$ for microbial group (B&C), and $p<0.01$ for both timeperiod and in 2-way analysis for (B only). D) The effect of microbial transplants on urinary formate levels over the course of the diet trial. ANOVA $p<0.001$ for microbial group and timeperiod. E) Renal calcium oxalate deposition based on Von Kossa staining of renal tissue sections. Arrows show calcium deposits stained black, which were quantified through an automated algorithm in QuPath. F) Quantification of renal calcium oxalate deposition by group. $p=0.014$; ANOVA. G,H) Pearson correlation between urinary oxalate ($R=0.7$, $p<0.001$) (G) or formate ($R=0.026$, $p=0.9$) (H) and renal calcium oxalate deposition. Where applicable, statistical significance was assessed through an ANOVA and post-hoc, Holm's corrected, paired t-tests. Statistical groups shown by blue letters.

Fig. S9. Microbial transplant composition impact on urinary inflammation, renal health, and overall mouse health for the metabolic cohort. A-G) The effect of microbial transplants on urinary IL-6 (A), IL-18 (B), and creatinine (C) levels, along with water intake (D), urine output (E), food intake (F) and body mass (G). Statistical significance - 2-way ANOVA (shown on charts) and post-hoc, Holm's corrected, paired t-tests. Blue letters reflect statistical groups between microbial transplants and *$p<0.05$, **$p<0.01$; ***$p<0.001$ for longitudinal comparisons within each microbial transplant group.

Fig. S10. Effect of microbial transplants on microbial community composition for the metabolic cohort. A,B) Beta-diversity analysis based on a weighted UniFrac dissimilarity matrix of colon feces collected at the end of the study period vs. stool (A; . $p=0.006$, PERMANOVA) or in the endpoint stool samples by group (B; no significant differences). Similar results for (B) were obtained with colon feces. Statistical differences shown by blue letters in legend. C) Within group beta-diversity distance of colon feces. $p=0.006$, one-way ANOVA. Blue letters represent statistical groups, based on post-hoc, Holm's corrected, paired t-tests. Similar results were found with endpoint stool samples. D) Change in phylogenetic diversity of stool for each microbial transplant group. Stats, shown on graph, are based on Pearson correlations. E) Normalized counts of transplanted microorganisms in the stool across timepoints and in the colon feces at the end of the diet trial.

bioRxiv preprint doi: https://doi.org/10.1101/2024.10.28.620613; this version posted October 28, 2024. The copyright holder for this preprint (which was not certified by peer review) is the author/funder, who has granted bioRxiv a license to display the preprint in perpetuity. It is made available under aCC-BY 4.0 International license.

Fig. S11. Effect of oxalate and formate on oxalate quantification using an enzymatic, ELISA-based assay. A) Correlation between oxalate or oxalate + formate, added to water, on oxalate concentration quantified through the enzymatic kit. B) Ratio of the calculated oxalate vs. the added concentration of oxalate and formate. C) The effect of adding oxalate or formate to urine, or of extracting oxalate from urine. R values and p-values (A) or p-values from one-way ANOVA (B,C) shown on chart. Blue letters represent statistical groups, based on pairwise t-tests.

bioRxiv preprint doi: https://doi.org/10.1101/2024.10.28.620613; this version posted October 28, 2024. The copyright holder for this preprint (which was not certified by peer review) is the author/funder, who has granted bioRxiv a license to display the preprint in perpetuity. It is made available under aCC-BY 4.0 International license.

Supplementary legends

Table S1. Diets used in animal studies.

Table S2. The number of host hepatic genes in each metabolic pathway that exhibited a significant increase (positive numbers) or decrease (negative numbers) in expression upon consumption of a 1.5% oxalate diet.

Table S3. The number of microbial metabolites in each metabolic pathway that exhibited a significant increase (positive numbers) or decrease (negative numbers) in expression upon consumption of a 1.5% oxalate diet by the host.

Table S4. The significant positive and negative correlations between microbial metabolites and host hepatic gene expression for those metabolites/genes significantly altered by 1.5% oxalate consumption. Data are listed with the host microbiome, metabollite and gene ID, long with correlation values and false discovery rate corrected p-values.

Table S5. The number of microbial genes in each metabolic pathway that exhibited a significant increase (positive numbers) or decrease (negative numbers) in abundance upon consumption of a 6% oxalate diet by the host.

Table S6. Media recipe used to test the impact of substrates on oxalate metabolism.

Table S7. Media recipe used to test the proportion of the NALB community that can utilize substrates as sole carbon and energy sources.

bioRxiv preprint doi: https://doi.org/10.1101/2024.10.28.620613; this version posted October 28, 2024. The copyright holder for this preprint (which was not certified by peer review) is the author/funder, who has granted bioRxiv a license to display the preprint in perpetuity. It is made available under aCC-BY 4.0 International license.

Supplementary Figures

Fig. S1

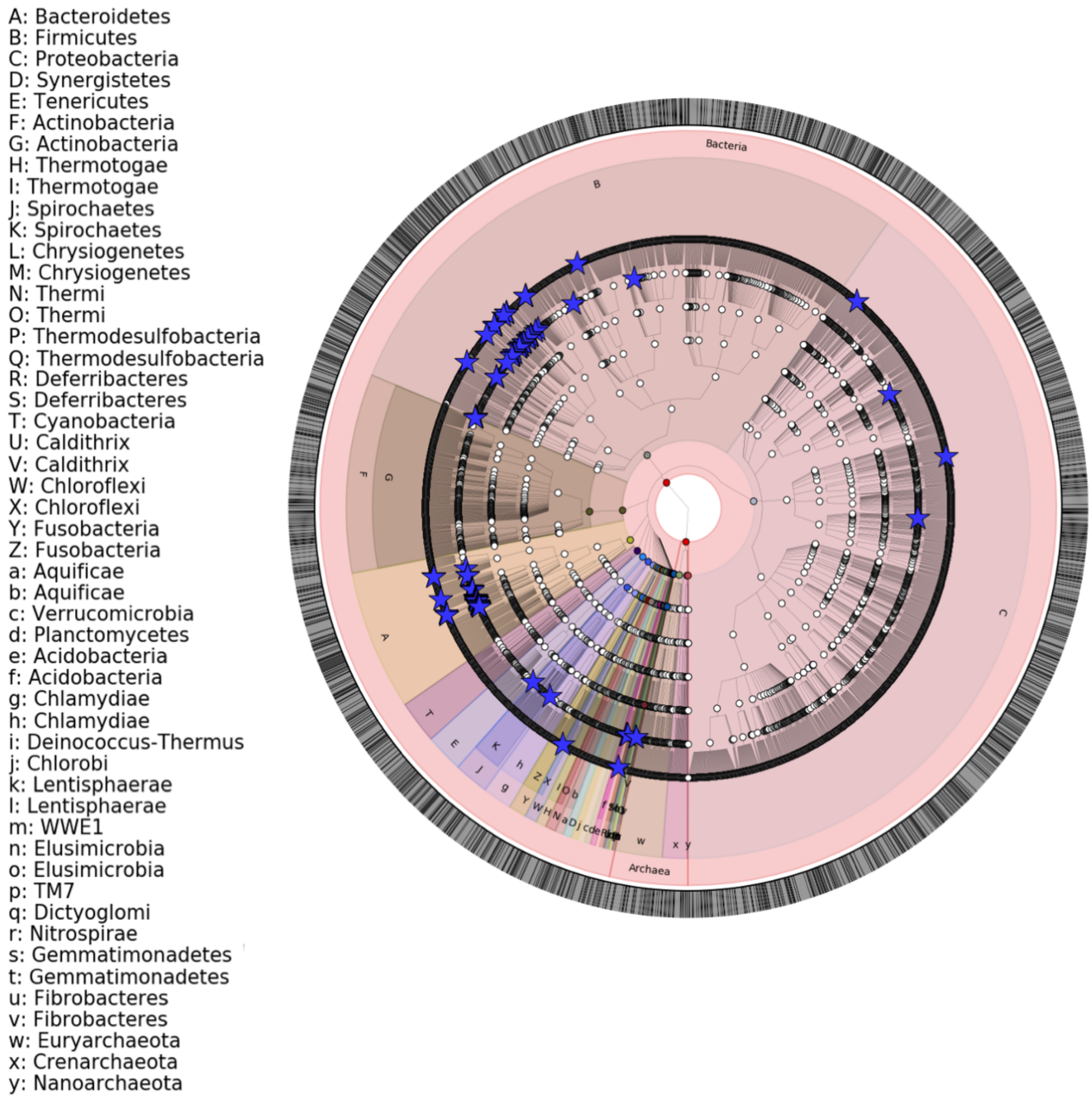

.

bioRxiv preprint doi: https://doi.org/10.1101/2024.10.28.620613; this version posted October 28, 2024. The copyright holder for this preprint (which was not certified by peer review) is the author/funder, who has granted bioRxiv a license to display the preprint in perpetuity. It is made available under aCC-BY 4.0 International license.

Fig. S2

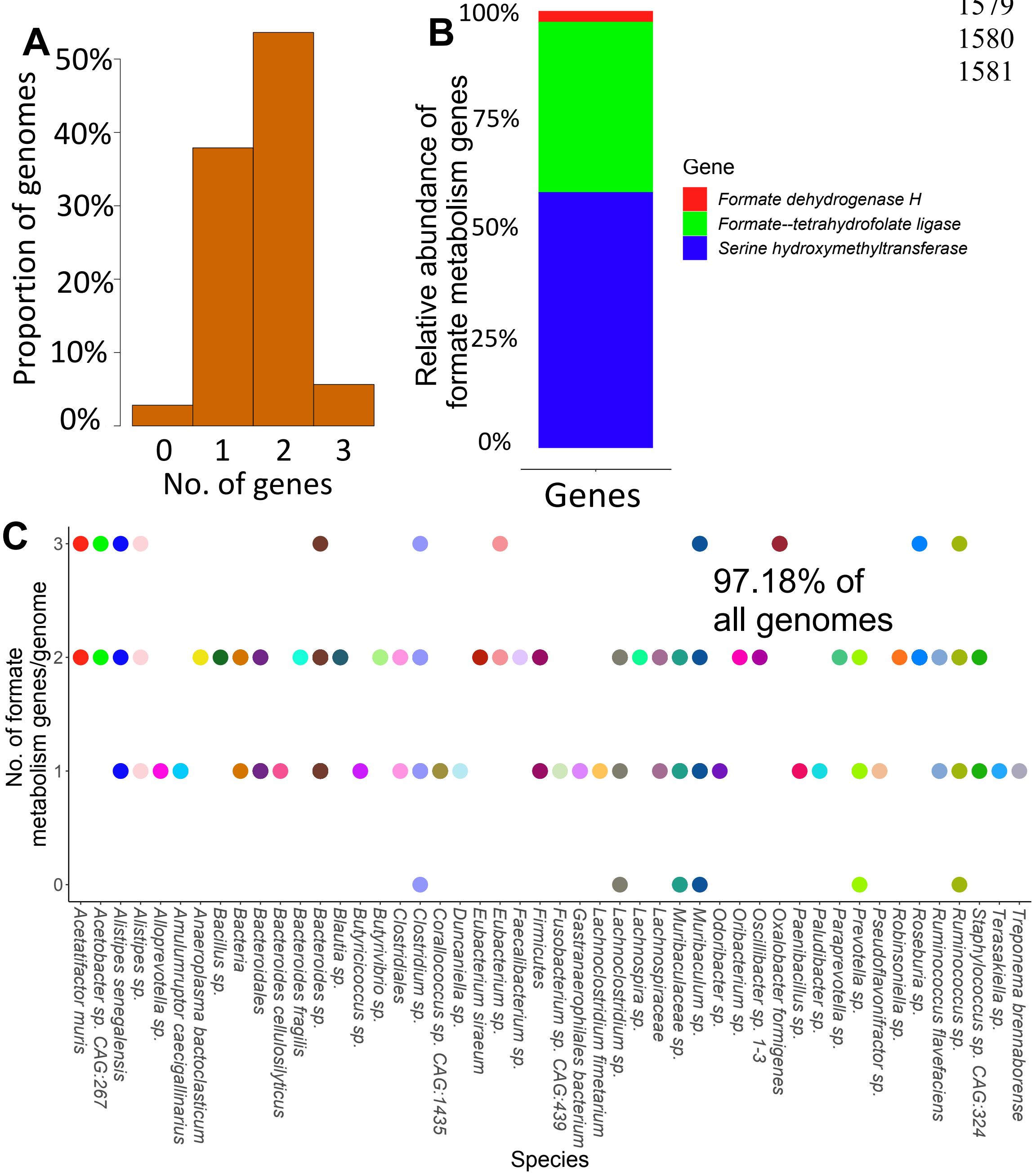

bioRxiv preprint doi: https://doi.org/10.1101/2024.10.28.620613; this version posted October 28, 2024. The copyright holder for this preprint (which was not certified by peer review) is the author/funder, who has granted bioRxiv a license to display the preprint in perpetuity. It is made available under aCC-BY 4.0 International license.

Fig. S3

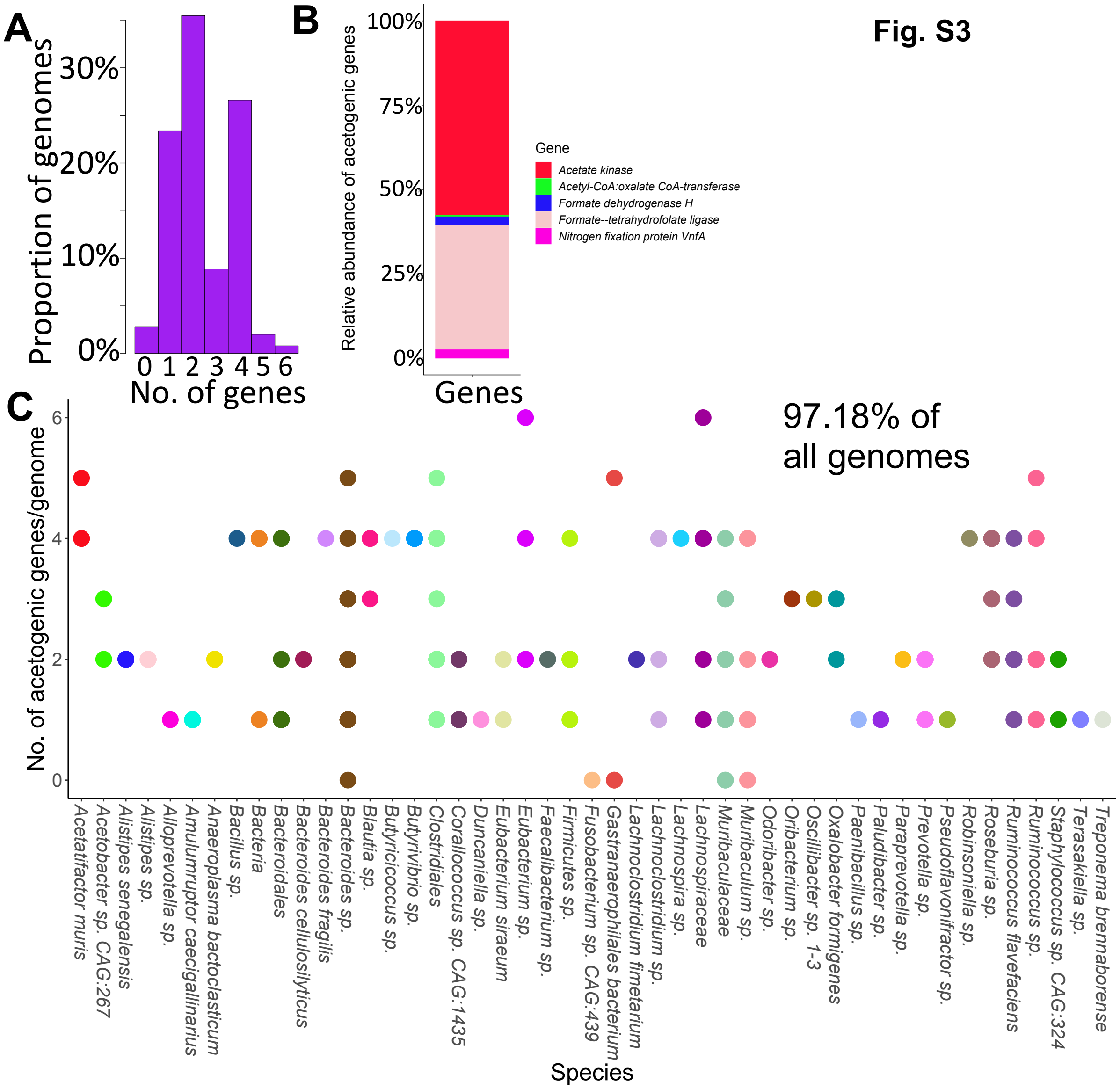

bioRxiv preprint doi: https://doi.org/10.1101/2024.10.28.620613; this version posted October 28, 2024. The copyright holder for this preprint (which was not certified by peer review) is the author/funder, who has granted bioRxiv a license to display the preprint in perpetuity. It is made available under aCC-BY 4.0 International license.

Fig. S4

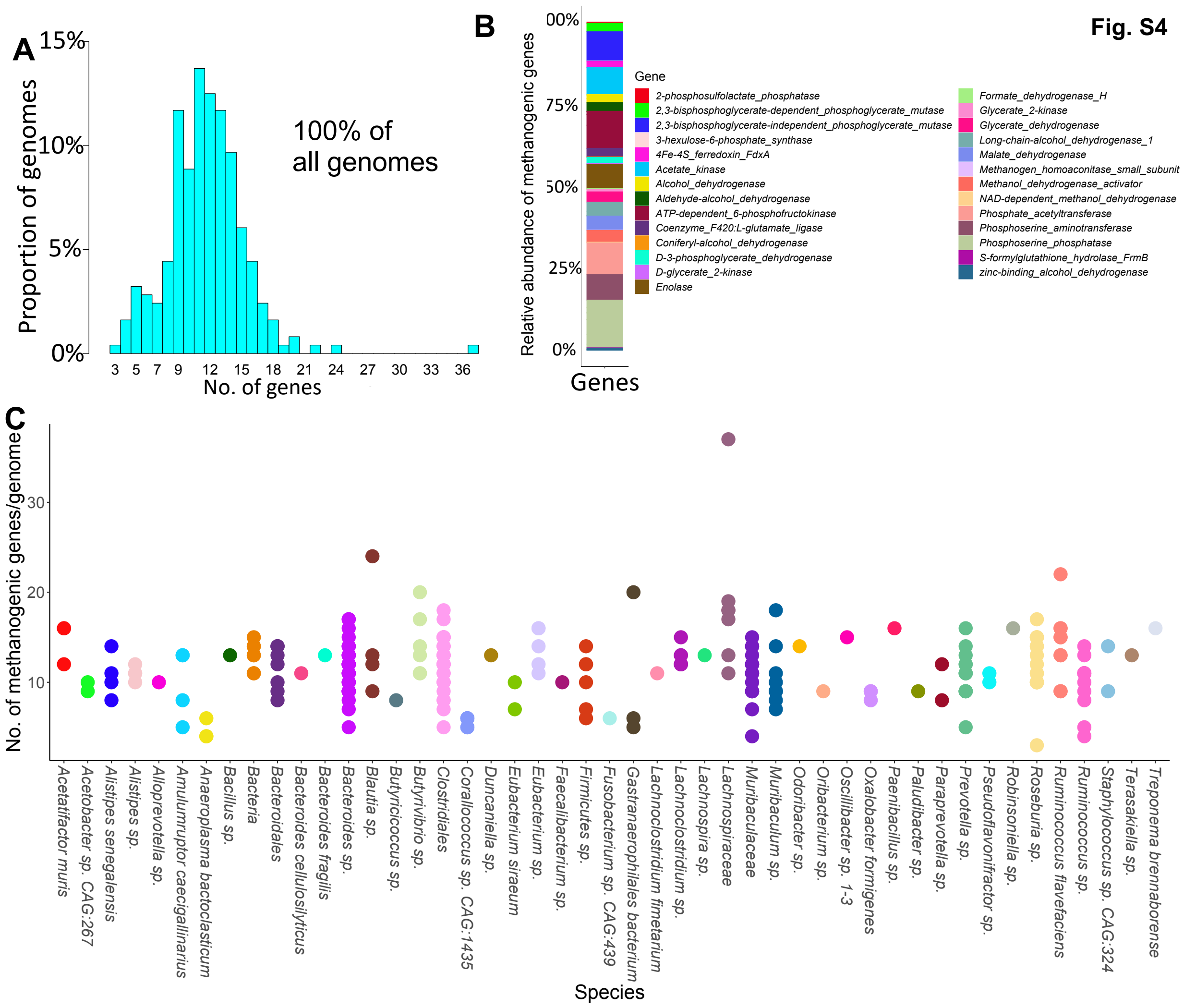

bioRxiv preprint doi: https://doi.org/10.1101/2024.10.28.620613; this version posted October 28, 2024. The copyright holder for this preprint (which was not certified by peer review) is the author/funder, who has granted bioRxiv a license to display the preprint in perpetuity. It is made available under aCC-BY 4.0 International license.

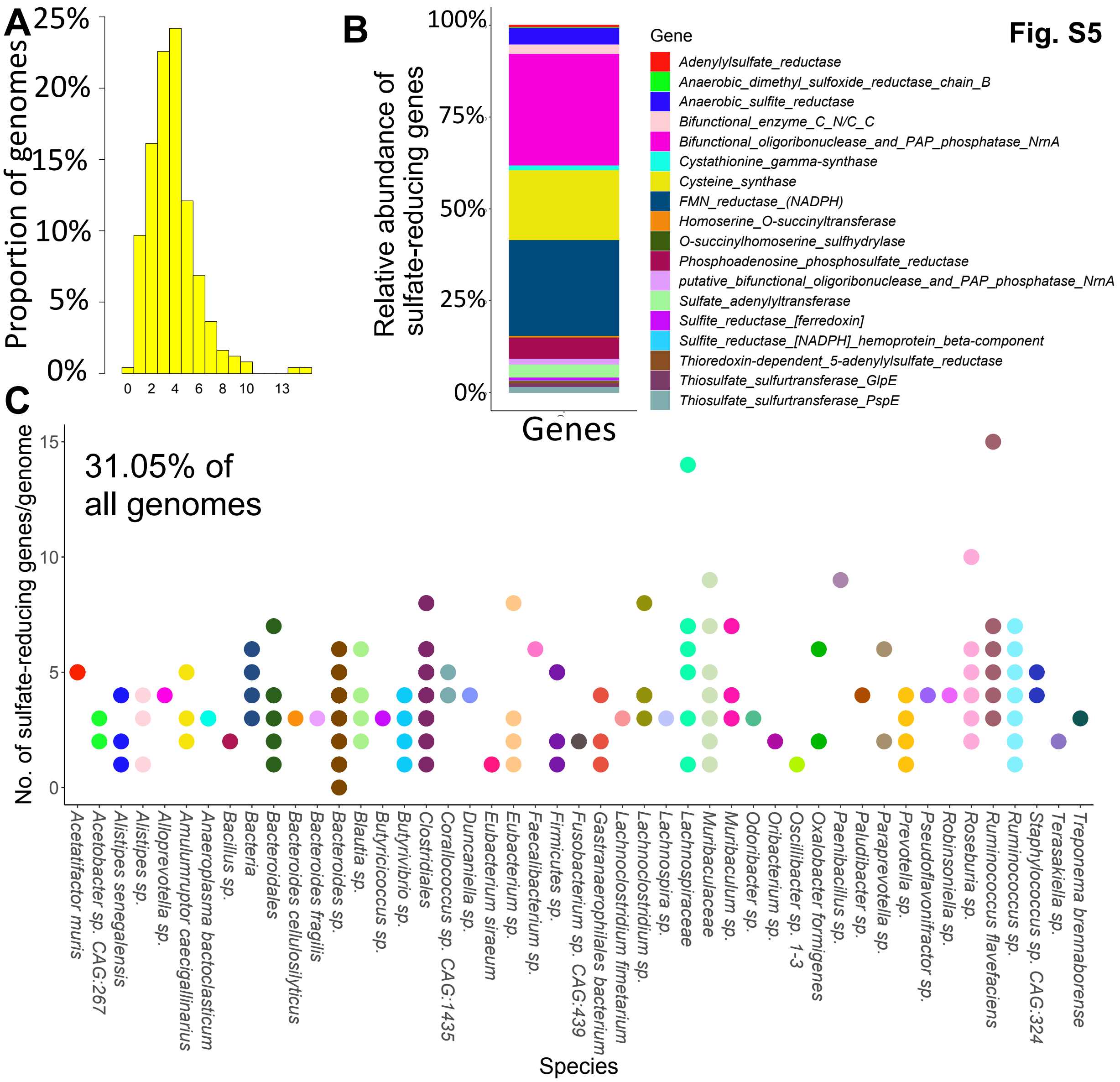

Fig. S5

bioRxiv preprint doi: https://doi.org/10.1101/2024.10.28.620613; this version posted October 28, 2024. The copyright holder for this preprint (which was not certified by peer review) is the author/funder, who has granted bioRxiv a license to display the preprint in perpetuity. It is made available under aCC-BY 4.0 International license.

Taxonomic cohort

Fig. S6

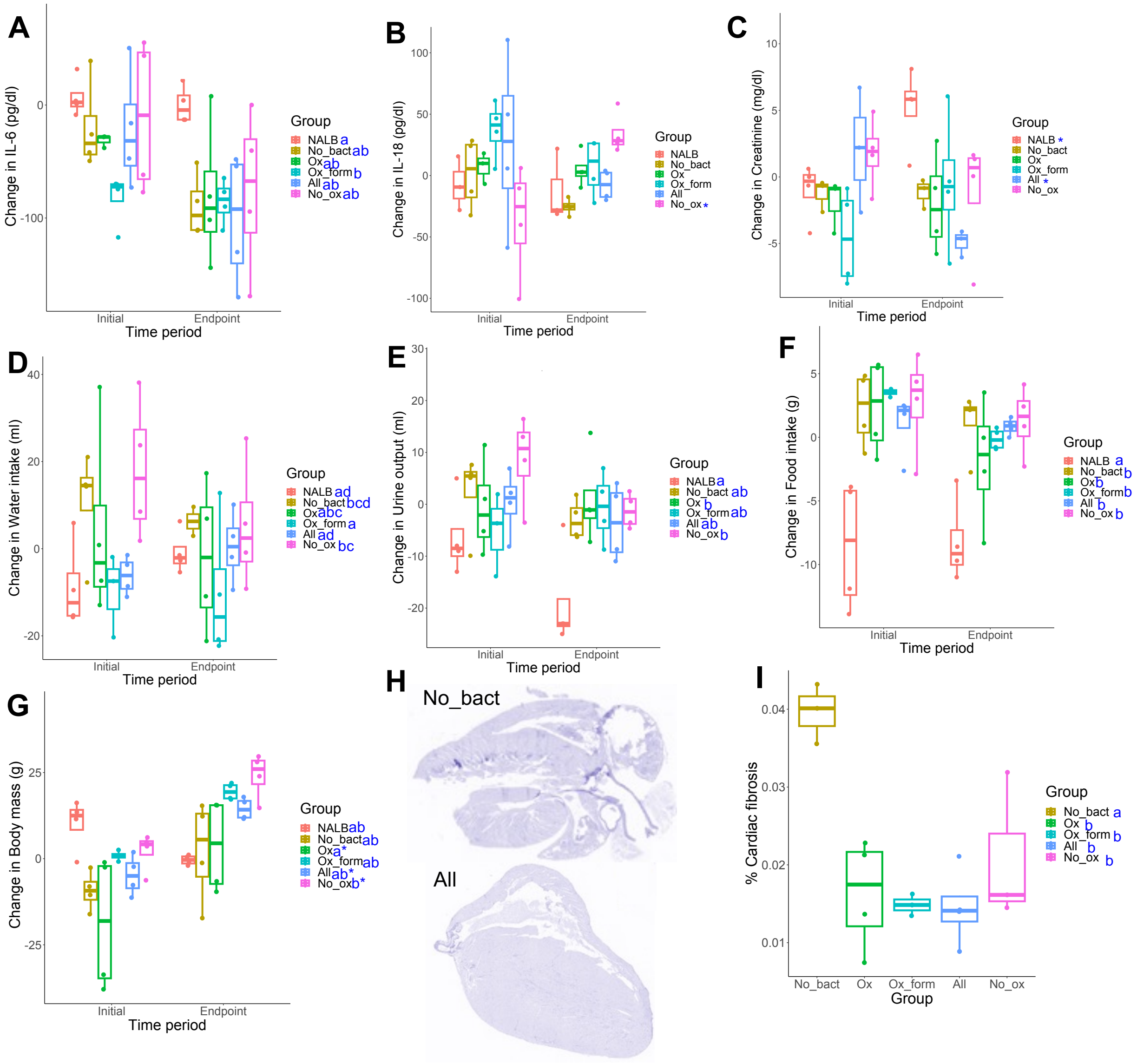

Group 1	No_bact
Group 2	Ox
Group 3	Ox_form
Group 4	All
Group 5	No_ox

bioRxiv preprint doi: https://doi.org/10.1101/2024.10.28.620613; this version posted October 28, 2024. The copyright holder for this preprint (which was not certified by peer review) is the author/funder, who has granted bioRxiv a license to display the preprint in perpetuity. It is made available under aCC-BY 4.0 International license.

Taxonomic cohort

Fig. S7

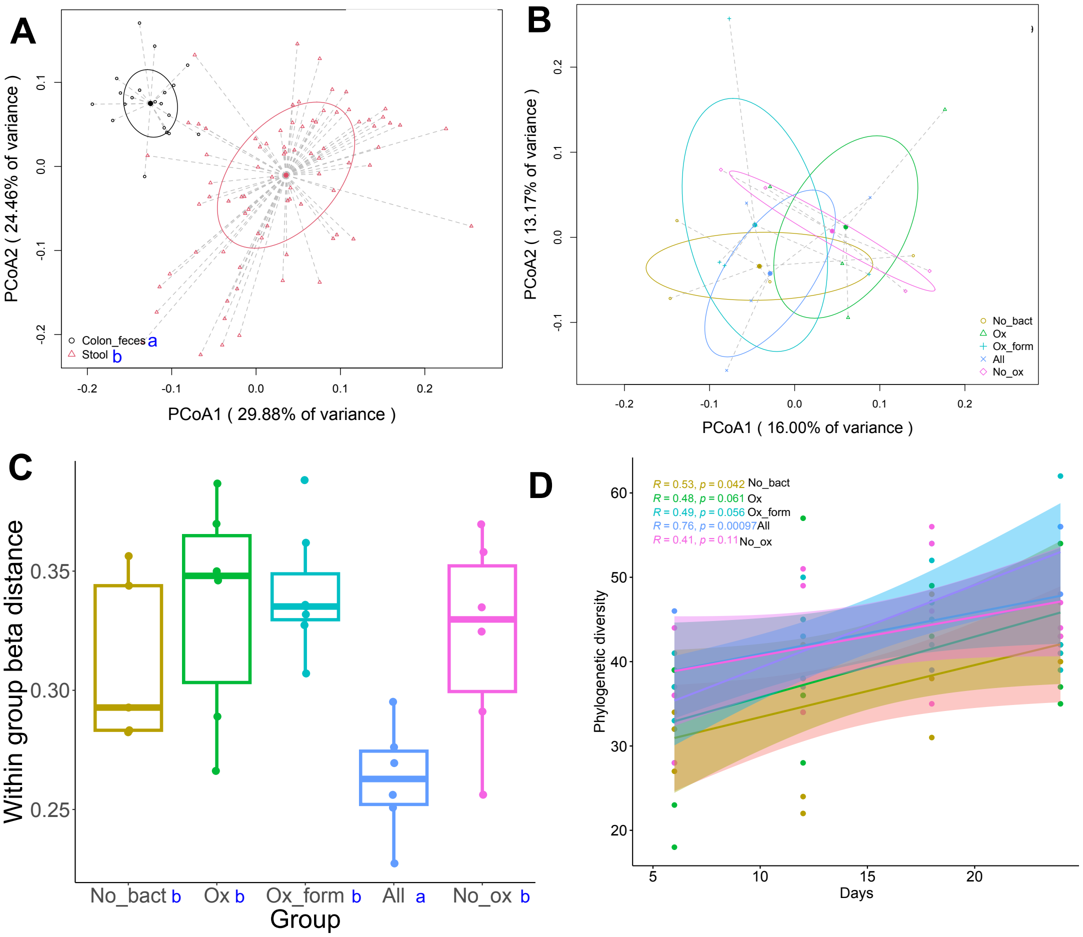

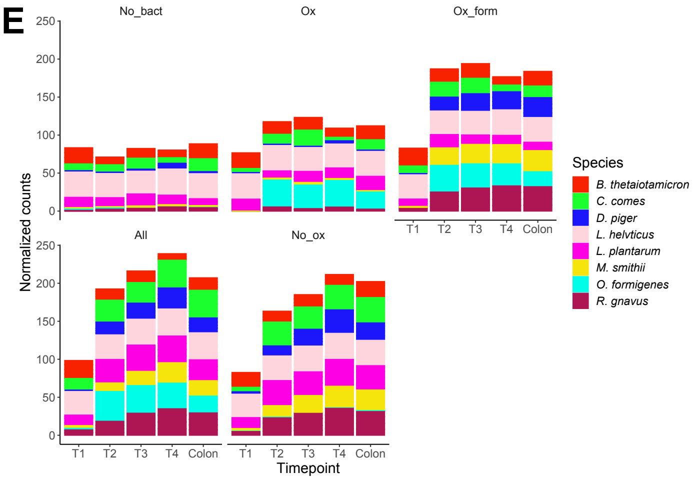

Group 1	No_bact
Group 2	Ox
Group 3	Ox_form
Group 4	All
Group 5	No_ox

bioRxiv preprint doi: https://doi.org/10.1101/2024.10.28.620613; this version posted October 28, 2024. The copyright holder for this preprint (which was not certified by peer review) is the author/funder, who has granted bioRxiv a license to display the preprint in perpetuity. It is made available under aCC-BY 4.0 International license.

Fig. S8

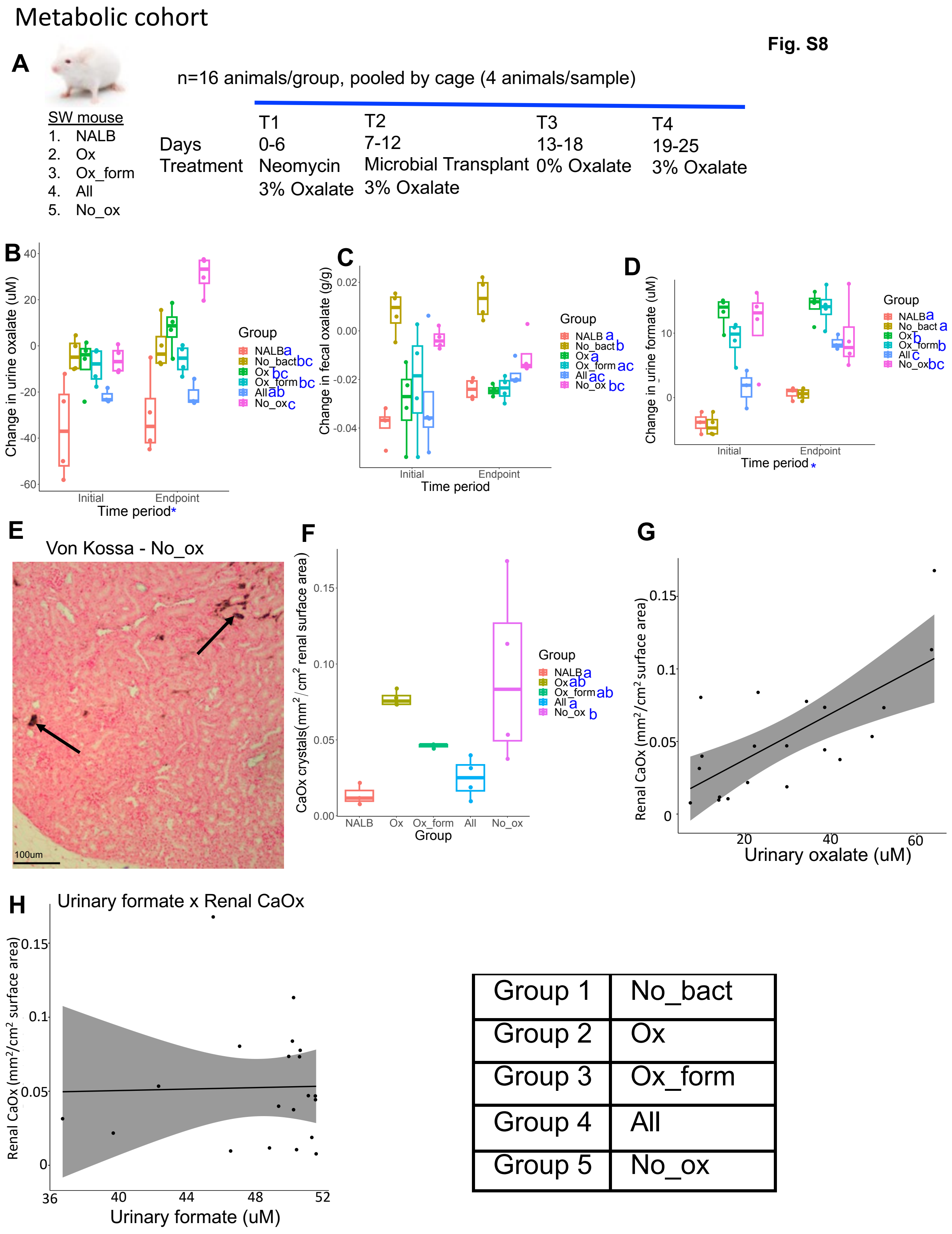

Group 1	No_bact
Group 2	Ox
Group 3	Ox_form
Group 4	All
Group 5	No_ox

bioRxiv preprint doi: https://doi.org/10.1101/2024.10.28.620613; this version posted October 28, 2024. The copyright holder for this preprint (which was not certified by peer review) is the author/funder, who has granted bioRxiv a license to display the preprint in perpetuity. It is made available under aCC-BY 4.0 International license.

Metabolic cohort

Fig. S9

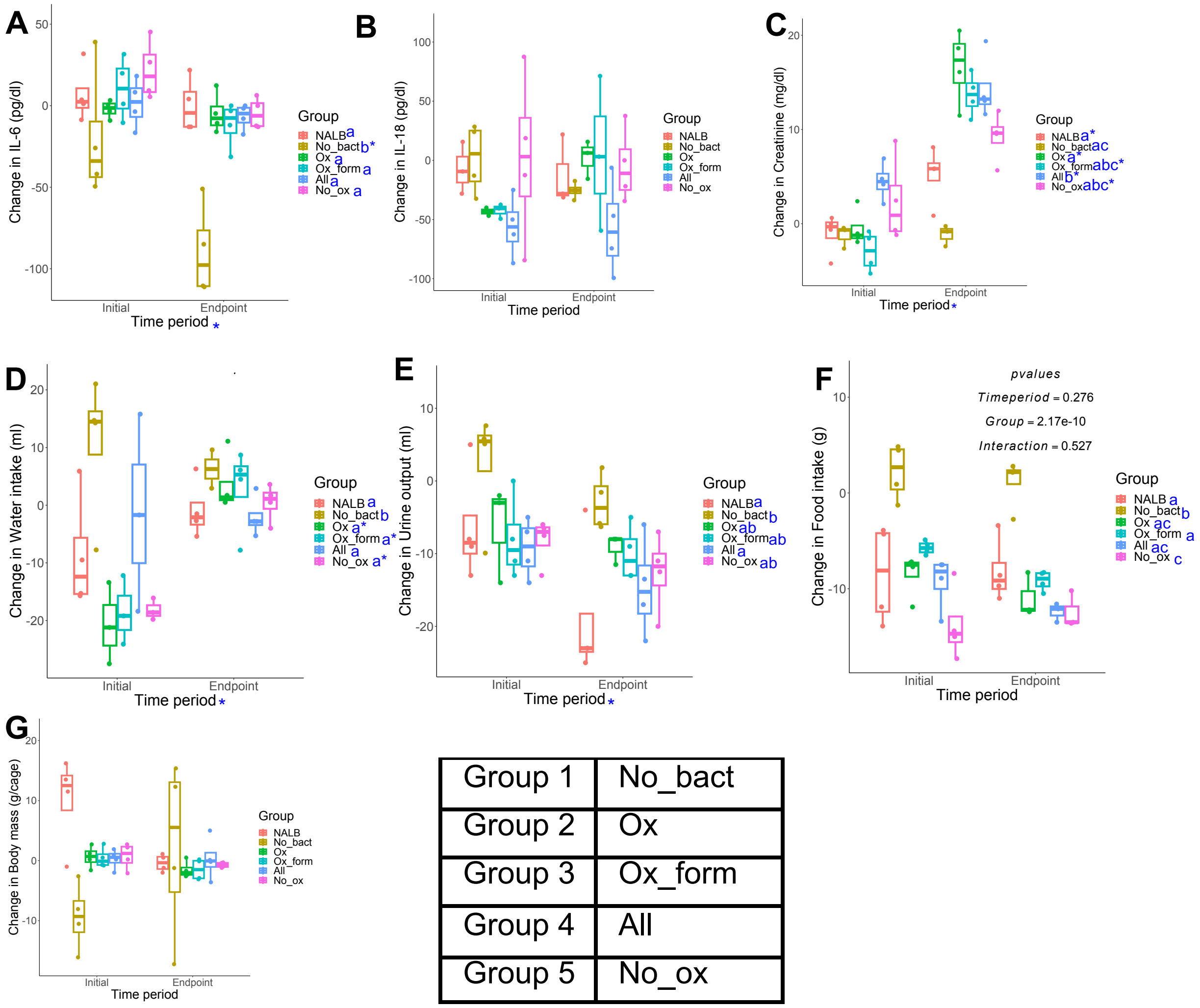

Group 1	No_bact
Group 2	Ox
Group 3	Ox_form
Group 4	All
Group 5	No_ox

bioRxiv preprint doi: https://doi.org/10.1101/2024.10.28.620613; this version posted October 28, 2024. The copyright holder for this preprint (which was not certified by peer review) is the author/funder, who has granted bioRxiv a license to display the preprint in perpetuity. It is made available under aCC-BY 4.0 International license.

Metabolic cohort

Fig. S10

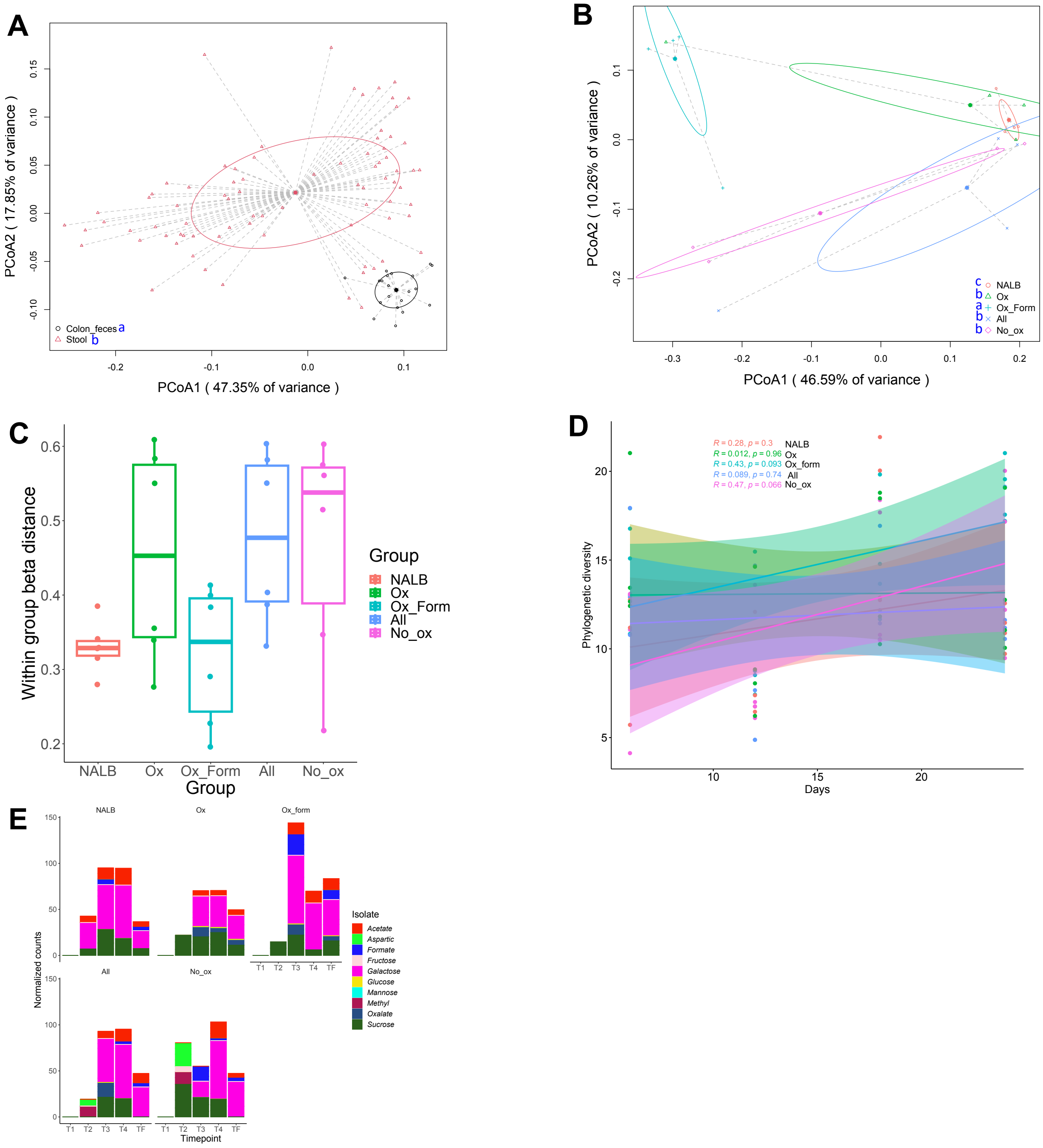

bioRxiv preprint doi: https://doi.org/10.1101/2024.10.28.620613; this version posted October 28, 2024. The copyright holder for this preprint (which was not certified by peer review) is the author/funder, who has granted bioRxiv a license to display the preprint in perpetuity. It is made available under aCC-BY 4.0 International license.

Fig. S11

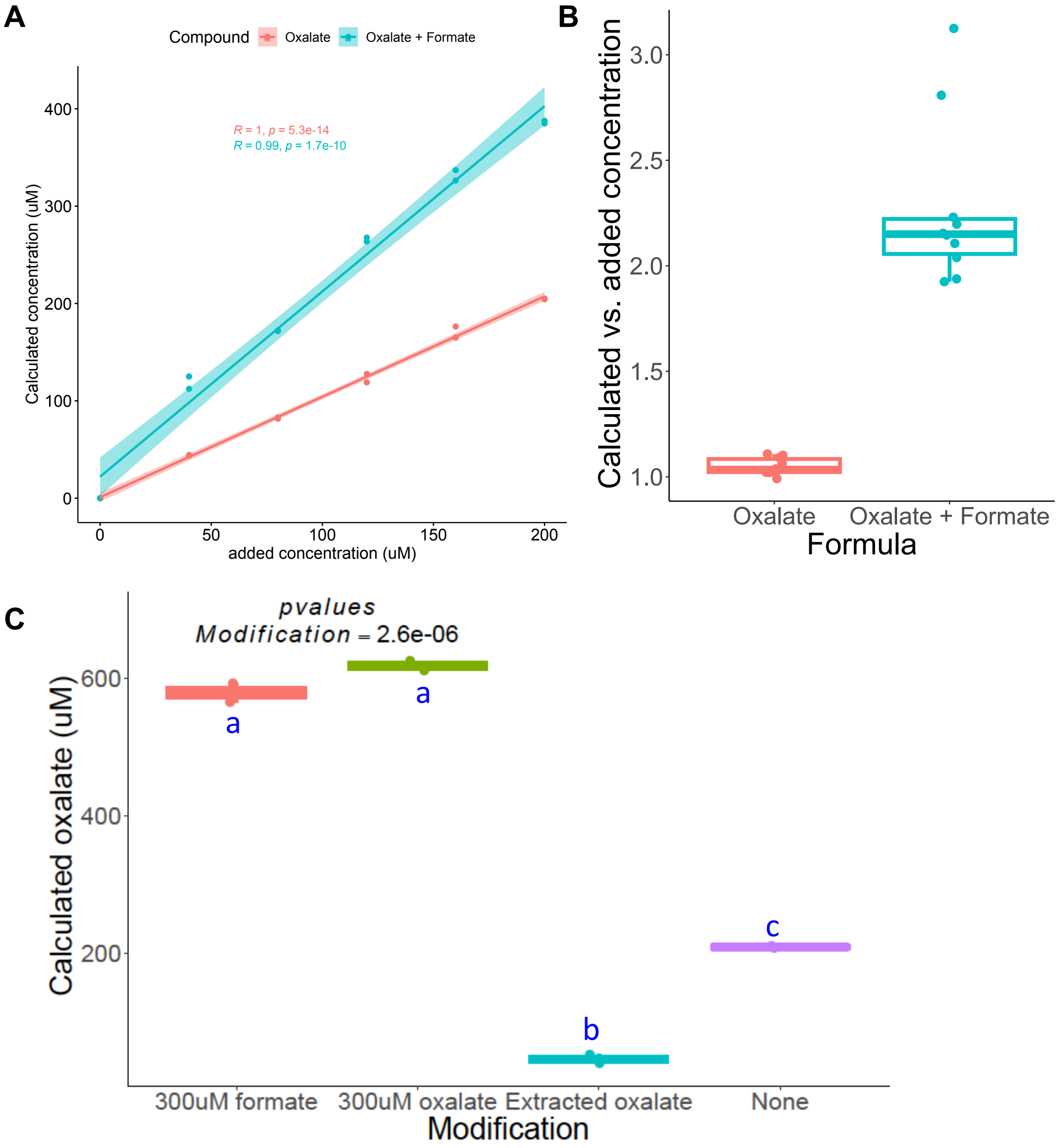

bioRxiv preprint doi: https://doi.org/10.1101/2024.10.28.620613; this version posted October 28, 2024. The copyright holder for this preprint (which was not certified by peer review) is the author/funder, who has granted bioRxiv a license to display the preprint in perpetuity. It is made available under aCC-BY 4.0 International license.

Supplementary Tables

Table S1. Composition of the diets used in the diet trials.

Ingredients	0% oxalate (g/Kg)	1.5% oxalate (g/Kg)	3% oxalate (g/Kg)
Casein	200	200	200
L-Cysteine	3	3	3
Corn Starch	285.5	NA	NA
Maltodextrin	120	120	120
Sucrose	100	185.4	185.4
Anhydrous Milkfat	NA	200	200
Soybean Oil	70	70	70
Cellulose	150	150	150
Mineral Mix	42	42	42
Vitamin Mix	12	12	12
Choline Bitartrate	2.5	2.5	2.5
TBHQ, antioxidant	0.014	0.054	0.054
Sodium Oxalate	0	15	30

bioRxiv preprint doi: https://doi.org/10.1101/2024.10.28.620613; this version posted October 28, 2024. The copyright holder for this preprint (which was not certified by peer review) is the author/funder, who has granted bioRxiv a license to display the preprint in perpetuity. It is made available under aCC-BY 4.0 International license.

Table S2. The number of host hepatic genes in each metabolic pathway that exhibited a significant increase or decrease in expression upon consumption of 1.5% oxalate.

Microbiome	tracking_id	gene	locus	Ave No Oxalate	Ave 1.5% Oxalate	log2(fold_change)	test_stat	p_value	FDR	direction	Protein	Gene ontology (biological process)	Gene ontology (cellular component)	Gene ontology (molecular function)
SWM	XLOC_000766	Spp2	3:37307748-37579096	3.89744	0.046197	-6.39858	-0.335453	5.00E-05	5.91E-03	Down	Secreted phosphoprotein 24 (Fragment)	bone remodeling	extracellular region	unknown
SWM	XLOC_000242	Zap70	12:111758848-111813873	2.12314	0.04184	-5.66517	-0.336216	5.00E-05	5.91E-03	Down	Tyrosine-protein kinase ZAP-70 (Fragment)	beta selection	cell-cell junction	ATP binding
SWM	XLOC_028591	Cd5l	6:127122965-127212411	2.02446	0.0425156	-5.5734	-0.161489	5.00E-05	5.91E-03	Down	CD5 antigen-like (Apoptosis inhibitor expressed by macrophages) (mAIM) (Apoptosis inhibitory 6) (SP-alpha)	apoptotic process	cytoplasm	scavenger receptor activity
SWM	XLOC_052270	Ndufb11	3:27182964-27284608	54.9449	1.76651	-4.95901	-3.01937	5.00E-05	5.91E-03	Down	NADH dehydrogenase [ubiquinone] 1 beta subcomplex subunit 11, mitochondrial (Complex I-ESSS) (CI-ESSS)	mitochondrial respiratory chain complex I ass	integral component of membrane	unknown
SWM	XLOC_014654	Rft1	7:119760922-119849010	1.29615	0.0571921	-4.50227	-0.265954	5.00E-05	5.91E-03	Down	Protein RFT1 homolog	carbohydrate transport	endoplasmic reticulum membrane	lipid transporter activity
SWM	XLOC_040594	Gm3375	11:3639784-3690477	11.5821	0.557754	-4.37612	-1.1918	5.00E-05	5.91E-03	Down	unknown	unknown	unknown	unknown
SWM	XLOC_041694	Tarsl2	5:33634951-33678995	1.02634	0.109071	-3.23417	-0.490847	5.00E-05	5.91E-03	Down	Probable threonine--tRNA ligase 2, cytoplasmic (EC 6.1.1.3) (Threonyl-tRNA synthetase) (ThrRS) (Threonyl-tl	threonyl-tRNA aminoacylation	cytoplasm	ATP binding
SWM	XLOC_016014	Fam25c	11:61517248-61579687	3.88844	0.590921	-2.71816	-1.48144	5.00E-05	5.91E-03	Down	Protein FAM25C	unknown	unknown	unknown
SWM	XLOC_008130	Leap2	17:53566860-53672720	8.2091	1.85957	-2.14226	-1.46723	5.00E-05	5.91E-03	Down	Liver-expressed antimicrobial peptide 2 (LEAP-2)	defense response to bacterium	extracellular region	unknown
SWM	XLOC_053271	Prdx4	8:35367749-35388140	26.6387	7.05001	-1.91782	-3.26408	5.00E-05	5.91E-03	Down	Peroxiredoxin-4 (Fragment)	4-hydroxyproline metabolic process	cytosol	antioxidant activity
SWM	XLOC_007693	Mrpl12	X:10707175-10719697	72.276	19.8756	-1.86252	-2.2074	5.00E-05	5.91E-03	Down	39S ribosomal protein L12, mitochondrial (L12mt) (MRP-L12)	mitochondrial transcription	mitochondrial large ribosomal subuni	structural constituent of ribosome
SWM	XLOC_040191	Ccnd2	6:108577038-108666925	53.2437	18.9519	-1.49027	-2.52067	5.00E-05	5.91E-03	Down	G1/S-specific cyclin-D2 (Fragment)	adult locomotory behavior	cyclin D2-CDK4 complex	unknown
SWM	XLOC_035338	Aacs	5:125475564-125517847	44.7957	16.0942	-1.47682	-2.34974	5.00E-05	5.91E-03	Down	Acetoacetyl-CoA synthetase (EC 6.2.1.16)	adipose tissue development	cytosol	acetoacetate-CoA ligase activity
SWM	XLOC_046996	Ankrd37	8:45975527-46029477	2.40161	0	NA	nan	5.00E-05	5.91E-03	Down	Ankyrin repeat domain-containing protein 37	unknown	cytoplasm	unknown
SWM	XLOC_010285	Slc25a47	X:48623412-48662294	1.22321	0	NA	nan	5.00E-05	5.91E-03	Down	Solute carrier family 25 member 47	unknown	integral component of membrane	unknown
SWM	XLOC_031741	0610037L13F	16:98095052-98095432	7.72747	0	NA	nan	5.00E-05	5.91E-03	Down	RIKEN cDNA 0610037L13 gene	unknown	unknown	unknown
SWM	XLOC_002201	Tyw5	12:98920573-98983238	1.11242	0	NA	nan	5.00E-05	5.91E-03	Down	tRNA wybutosine-synthesizing protein 5 (EC 1.14.11.42) (tRNA(Phe) (7-(3-amino-3-carboxypropyl)wyosine(3	wybutosine biosynthetic process	unknown	iron ion binding
SWM	XLOC_033898	-	2:91071073-91071619	3.30154	0	NA	nan	5.00E-05	5.91E-03	Down	unknown	unknown	unknown	unknown
SWM	XLOC_047019	-	5:87322395-87322857	3.19854	0	NA	nan	5.00E-05	5.91E-03	Down	unknown	unknown	unknown	unknown
SWM	XLOC_003554	-	1:179831052-179831964	1.45818	0	NA	nan	5.00E-05	5.91E-03	Down	unknown	unknown	unknown	unknown
SWM	XLOC_006208	-	1:194467979-194468321	73.9843	0	NA	nan	5.00E-05	5.91E-03	Down	unknown	unknown	unknown	unknown
SWM	XLOC_020119	-	14:31866306-31866457	121.302	0	NA	nan	5.00E-05	5.91E-03	Down	unknown	unknown	unknown	unknown
SWM	XLOC_020135	-	16:70998967-70999115	287.223	0	NA	nan	5.00E-05	5.91E-03	Down	unknown	unknown	unknown	unknown
SWM	XLOC_022306	Rps14	17:27628167-27636862	444.001	951.25	1.09926	1.8632	5.00E-05	5.91E-03	Up	40S ribosomal protein S14	erythrocyte differentiation	cytosolic small ribosomal subunit	mRNA 5'-UTR binding
SWM	XLOC_011468	Xrcc3	11:83746939-83752642	663.108	1421.64	1.10024	2.00737	5.00E-05	5.91E-03	Up	DNA repair protein XRCC3 (X-ray repair cross-complementing protein 3)	DNA repair	cytoplasm	ATP binding
SWM	XLOC_017459	Lgals1	11:53350739-53423188	192.239	417.359	1.11839	1.86222	5.00E-05	5.91E-03	Up	Galectin-1 (Gal-1) (14 kDa lectin) (Beta-galactoside-binding lectin L-14-I) (Galaptin) (Lactose-binding lectin 1	apoptotic process	cell surface	carbohydrate binding
SWM	XLOC_019066	Eif4a2	7:28971371-28981864	123.61	272.874	1.14244	1.94689	5.00E-05	5.91E-03	Up	Eukaryotic initiation factor 4A-II	cellular response to leukemia inhibitory facto	unknown	ATP-dependent RNA helicase activity
SWM	XLOC_043396	Eif3k	4:129591959-129600648	101.57	227.731	1.16486	2.03664	5.00E-05	5.91E-03	Up	Eukaryotic translation initiation factor 3 subunit K	regulation of translational initiation	eukaryotic translation initiation facto	ribosome binding
SWM	XLOC_026412	Rabepk	11:70525366-70527858	313.219	713.628	1.188	2.03536	5.00E-05	5.91E-03	Up	Rab9 effector protein with kelch motifs	unknown	endosome membrane	unknown
SWM	XLOC_012145	Gm11361	13:97751111-97760965	493.411	1132.5	1.19865	2.07551	5.00E-05	5.91E-03	Up	MCG116671	translation	ribosome	RNA binding
SWM	XLOC_008282	Epn2	4:41569774-41647594	113.905	266.464	1.22612	2.06514	5.00E-05	5.91E-03	Up	Epsin-2	endocytosis	clathrin coat of endocytic vesicle	unknown
SWM	XLOC_010981	Nemf	19:3908760-3912797	181.505	425.086	1.22774	2.11908	5.00E-05	5.91E-03	Up	Nuclear export mediator factor Nemf (Serologically defined colon cancer antigen 1 homolog)	nuclear export	nucleus	unknown
SWM	XLOC_016357	Psmb5	11:97703398-97713500	176.08	413.427	1.2314	2.07886	5.00E-05	5.91E-03	Up	Proteasome subunit beta type-5 (EC 3.4.25.1) (Macropain epsilon chain) (Multicatalytic endopeptidase comp	proteasome-mediated ubiquitin-dependent p	centrosome	peptidase activity
SWM	XLOC_019562	Mzt2	10:128490858-128494145	458.265	1101.62	1.26537	2.11043	5.00E-05	5.91E-03	Up	Mitotic-spindle organizing protein 2 (Mitotic-spindle organizing protein associated with a ring of gamma-tul	unknown	centrosome	unknown
SWM	XLOC_046046	Use1	4:116066939-116075097	697.802	1693.95	1.2795	2.21912	5.00E-05	5.91E-03	Up	Vesicle transport protein USE1	unknown	endoplasmic reticulum	unknown
SWM	XLOC_051419	Rps24-ps3	6:71124609-71124963	121.931	296.177	1.2804	1.99821	5.00E-05	5.91E-03	Up	unknown	unknown	unknown	unknown
SWM	XLOC_040030	Rpl32	1:39367841-39478755	438.388	1064.89	1.28042	2.14979	5.00E-05	5.91E-03	Up	60S ribosomal protein L32	cellular response to dexamethasone stimulus	cytosolic large ribosomal subunit	structural constituent of ribosome
SWM	XLOC_040769	Rps5	9:66946085-66949516	166.062	405.679	1.28862	2.14341	5.00E-05	5.91E-03	Up	40S ribosomal protein S5 [Cleaved into: 40S ribosomal protein S5, N-terminally processed]	regulation of translational fidelity	cytosolic small ribosomal subunit	mRNA binding
SWM	XLOC_028744	S100a10	3:90511033-90524581	256.241	634.327	1.30772	2.1224	5.00E-05	5.91E-03	Up	Uncharacterized protein	unknown	integral component of membrane	calcium ion binding
SWM	XLOC_018276	Tsta3	4:45105207-45108114	44.9318	112.059	1.31846	2.01424	5.00E-05	5.91E-03	Up	GDP-L-fucose synthase (EC 1.1.1.271) (GDP-4-keto-6-deoxy-D-mannose-3,5-epimerase-4-reductase) (Protein	'de novo' GDP-L-fucose biosynthetic process	unknown	coenzyme binding
SWM	XLOC_041146	Rps16	10:80292452-80294114	1452.2	3648.55	1.32908	2.13331	5.00E-05	5.91E-03	Up	40S ribosomal protein S16	cellular response to leukemia inhibitory facto	cytosolic small ribosomal subunit	RNA binding
SWM	XLOC_006118	Myl6	17:56175552-56184185	59.7204	151.88	1.34664	1.9909	5.00E-05	5.91E-03	Up	Myosin light polypeptide 6 (17 kDa myosin light chain) (LC17) (Myosin light chain 3) (MLC-3) (Myosin light ch	muscle contraction	brush border	actin-dependent ATPase activity
SWM	XLOC_052377	Rpl39	11:114668523-114672331	497.073	1278.62	1.36306	2.34475	5.00E-05	5.91E-03	Up	60S ribosomal protein L39	antibacterial humoral response	cytosolic large ribosomal subunit	RNA binding
SWM	XLOC_016432	Gjb2	7:140093394-140102441	105.546	271.658	1.36392	2.2596	5.00E-05	5.91E-03	Up	Gap junction protein	cell communication	connexin complex	unknown
SWM	XLOC_006803	B9d1	5:137981408-137991543	784.532	2036.25	1.37601	2.07244	5.00E-05	5.91E-03	Up	B9 domain-containing protein 1 (Fragment)	camera-type eye development	membrane	hedgehog receptor activity
SWM	XLOC_033148	Dio1	7:127256958-127260709	92.4545	241.511	1.38527	2.27822	5.00E-05	5.91E-03	Up	Iodothyronine deiodinase	hormone biosynthetic process	unknown	thyroxine 5'-deiodinase activity
SWM	XLOC_046164	Ndufb7	X:20615236-20650901	281.881	740.298	1.39302	2.19893	5.00E-05	5.91E-03	Up	NADH dehydrogenase [ubiquinone] 1 beta subcomplex subunit 7 (Complex I-B18) (CI-B18) (NADH-ubiquinone	mitochondrial respiratory chain complex I ass	mitochondrial inner membrane	NADH dehydrogenase (ubiquinone) activity
SWM	XLOC_006270	Gm11946	4:3973091-3973595	1354.15	3561.57	1.39513	2.35491	5.00E-05	5.91E-03	Up	unknown	unknown	unknown	unknown
SWM	XLOC_008555	Rpl23a	11:98001507-98030492	861.151	2294.02	1.41354	2.20997	5.00E-05	5.91E-03	Up	60S ribosomal protein L23a	ribosomal large subunit assembly	cytosolic large ribosomal subunit	large ribosomal subunit rRNA binding
SWM	XLOC_007579	Rpl38	X:134585653-134588062	263.08	708.445	1.42915	2.43573	5.00E-05	5.91E-03	Up	60S ribosomal protein L38	90S preribosome assembly	cytosolic large ribosomal subunit	RNA binding
SWM	XLOC_018265	9030619P08I	6:116624015-116741284	119.655	324.737	1.4404	2.21879	5.00E-05	5.91E-03	Up	Uncharacterized protein	unknown	unknown	unknown
SWM	XLOC_023011	Fau	14:34351697-34358242	113.404	308.165	1.44223	2.25368	5.00E-05	5.91E-03	Up	Ubiquitin-like protein FUBI (Monoclonal non-specific suppressor factor beta) (MNSF-beta)	antibacterial humoral response	cytosolic small ribosomal subunit	unknown
SWM	XLOC_009749	Gm22513	8:129252997-129253594	55.2929	150.892	1.44835	2.31224	5.00E-05	5.91E-03	Up	unknown	unknown	unknown	unknown
SWM	XLOC_020823	Rpl36	2:38996712-39016057	402.655	1099.28	1.44894	1.99129	5.00E-05	5.91E-03	Up	60S ribosomal protein L36	cytoplasmic translation	cytosolic large ribosomal subunit	RNA binding
SWM	XLOC_027453	Cst3	10:110919788-110939622	31.62	87.1833	1.46321	2.05231	5.00E-05	5.91E-03	Up	Cystatin (Fragment)	unknown	unknown	cysteine-type endopeptidase inhibitor activity
SWM	XLOC_023974	Usmg5	8:71366847-71369869	80.1252	223.859	1.48226	2.41719	5.00E-05	5.91E-03	Up	Up-regulated during skeletal muscle growth protein 5 (Diabetes-associated protein in insulin-sensitive tissue	unknown	integral component of membrane	unknown
SWM	XLOC_028647	Dpm3	4:107291464-107307169	27.1944	76.7989	1.49778	2.36228	5.00E-05	5.91E-03	Up	Dolichol-phosphate mannosyltransferase subunit 3 (Dolichol-phosphate mannose synthase subunit 3) (DPM 3	GPI anchor biosynthetic process	dolichol-phosphate-mannose synthas	dolichyl-phosphate beta-D-mannosyltransferase activity
SWM	XLOC_045780	Zdhhc2	1:36761797-36782818	11.3803	32.1737	1.49935	2.09276	5.00E-05	5.91E-03	Up	Palmitoyltransferase ZDHHC2 (EC 2.3.1.225) (Zinc finger DHHC domain-containing protein 2) (DHHC-2)	protein palmitoylation	endoplasmic reticulum	palmitoyltransferase activity
SWM	XLOC_035208	Tbx3	7:65644888-65701913	49.567	141.618	1.51455	2.18414	5.00E-05	5.91E-03	Up	T-box transcription factor TBX3 (T-box protein 3)	animal organ morphogenesis	nucleus	DNA binding transcription factor activity
SWM	XLOC_010234	Serpina3c	12:104028768-104044443	6.22892	18.0525	1.53514	2.29542	5.00E-05	5.91E-03	Up	Serine (Or cysteine) peptidase inhibitor, clade A, member 3C	unknown	extracellular space	unknown
SWM	XLOC_030162	S100a1	7:12922289-12926686	561.523	1666.53	1.56943	2.47885	5.00E-05	5.91E-03	Up	Protein S100-A1 (S-100 protein alpha chain) (S-100 protein subunit alpha) (S100 calcium-binding protein A1)	negative regulation of transcription by RNA p	A band	ATPase binding
SWM	XLOC_048544	Rps27l	3:90212521-90213651	2306.64	6850.25	1.57036	2.60517	5.00E-05	5.91E-03	Up	40S ribosomal protein S27-like	activation of cysteine-type endopeptidase act	cytosolic small ribosomal subunit	cysteine-type endopeptidase activator activity involved in apoptotic proce
SWM	XLOC_033564	Rpl11	17:28328470-28331201	493.661	1477.62	1.58168	2.57486	5.00E-05	5.91E-03	Up	60S ribosomal protein L11	cytoplasmic translation	cytoplasm	5S rRNA binding
SWM	XLOC_006123	Rpl41	X:37082519-37085402	162.611	486.862	1.58209	2.61288	5.00E-05	5.91E-03	Up	60S ribosomal protein L41	cytoplasmic translation	cytosolic large ribosomal subunit	mRNA 3'-UTR binding
SWM	XLOC_007216	Supt4a	3:149982947-150073619	315.7	948.858	1.58764	2.29477	5.00E-05	5.91E-03	Up	MCG7669, isoform CRA_b (Suppressor of Ty 4A)	negative regulation of transcription elongatic	nucleus	zinc ion binding
SWM	XLOC_024885	Metap1d	14:41151228-41166864	141.202	426.959	1.59634	2.47192	5.00E-05	5.91E-03	Up	Methionine aminopeptidase 1D, mitochondrial (MAP 1D) (MetAP 1D) (EC 3.4.11.18) (Methionyl aminopeptid	unknown	intracellular membrane-bounded orga	aminopeptidase activity
SWM	XLOC_046417	Tmem208	9:21920795-21948907	999.986	3028.35	1.59855	2.39918	5.00E-05	5.91E-03	Up	Transmembrane protein 208	autophagy	endoplasmic reticulum membrane	unknown
SWM	XLOC_011368	Serpina12	11:16257723-16508484	103.745	314.816	1.60147	2.28737	5.00E-05	5.91E-03	Up	Serpin A12 (Visceral adipose tissue-derived serine protease inhibitor) (Vaspin) (Visceral adipose-specific ser	negative regulation of gluconeogenesis	extracellular space	serine-type endopeptidase inhibitor activity
SWM	XLOC_048845	Rbp1	6:48504336-48572789	764.993	2411.72	1.65654	2.48797	5.00E-05	5.91E-03	Up	Retinol-binding protein 1 (Cellular retinol-binding protein) (CRBP) (Cellular retinol-binding protein I) (CRBP-I)	lipid homeostasis	cell body	all-trans-retinol binding
SWM	XLOC_012728	Gm4149	7:3181092-3181973	32.6968	104.378	1.6746	2.57197	5.00E-05	5.91E-03	Up	unknown	unknown	unknown	unknown
SWM	XLOC_005976	Lyz2	15:78926724-78930465	30.8477	99.4177	1.68834	2.48288	5.00E-05	5.91E-03	Up	Lysozyme C-2 (EC 3.2.1.17) (1,4-beta-N-acetylmuramidase C) (Lysozyme C type M)	cell wall macromolecule catabolic process	cytoplasm	hydrolase activity, acting on glycosyl bonds
SWM	XLOC_042707	Mrpl23	4:155802877-155809975	64.8571	210.734	1.70009	2.33899	5.00E-05	5.91E-03	Up	39S ribosomal protein L23, mitochondrial	translation	ribosome	structural constituent of ribosome
SWM	XLOC_019159	Stfa2l1	X:73787027-73790997	190.705	620.608	1.70234	2.54113	5.00E-05	5.91E-03	Up	Stefin A2 protein	unknown	intracellular	cysteine-type endopeptidase inhibitor activity
SWM	XLOC_023510	Gstp1	3:107877228-107898686	20.2903	67.5885	1.73598	2.43173	5.00E-05	5.91E-03	Up	Glutathione S-transferase P 1 (Gst P1) (EC 2.5.1.18) (GST YF-YF) (GST class-pi) (GST-piB) (Preadipocyte grov	animal organ regeneration	cytoplasm	dinitrosyl-iron complex binding
SWM	XLOC_021660	Mydgf	4:61670176-61674136	46.1031	159.881	1.79406	2.27763	5.00E-05	5.91E-03	Up	Myeloid-derived growth factor (MYDGF) (Interleukin-25) (IL-25) (Stromal cell-derived growth factor SF20)	angiogenesis	endoplasmic reticulum-Golgi interme	unknown
SWM	XLOC_053235	Gm6472	15:81972389-81972845	158.294	568.245	1.84391	3.03377	5.00E-05	5.91E-03	Up	unknown	unknown	unknown	unknown
SWM	XLOC_003125	Gm16418	5:3236430-3236868	575.417	2094.36	1.86383	2.89284	5.00E-05	5.91E-03	Up	unknown	unknown	unknown	unknown
SWM	XLOC_004295	Rps15	18:60774450-60800944	492.493	1902.12	1.94943	2.87883	5.00E-05	5.91E-03	Up	40S ribosomal protein S15	translation	small ribosomal subunit	RNA binding
SWM	XLOC_038129	Rpl34-ps1	9:78175302-78175633	121.145	471.672	1.96105	2.91932	5.00E-05	5.91E-03	Up	unknown	unknown	unknown	unknown
SWM	XLOC_030860	Rpsa-ps10	X:79195046-79195448	39.3222	157.303	2.00013	3.31129	5.00E-05	5.91E-03	Up	unknown	unknown	unknown	unknown
SWM	XLOC_017518	Gm5805	13:75644895-75645171	214.745	932.559	2.11857	2.98716	5.00E-05	5.91E-03	Up	unknown	unknown	unknown	unknown
SWM	XLOC_032991	Mrpl48-ps	7:103812523-103813996	113.169	496.085	2.13211	3.28464	5.00E-05	5.91E-03	Up	unknown	unknown	unknown	unknown
SWM	XLOC_003457	-	7:6131489-6156447	14.2393	64.7037	2.18397	3.75027	5.00E-05	5.91E-03	Up	unknown	unknown	unknown	unknown
SWM	XLOC_010237	Serpina3j	12:104146381-104153870	1471.69	8229.85	2.48339	2.70233	5.00E-05	5.91E-03	Up	MCG54087 (Serine (or cysteine) peptidase inhibitor, clade A (alpha-1 antiproteinase, antitrypsin), member 3	unknown	extracellular space	serine-type endopeptidase inhibitor activity
SWM	XLOC_000857	Gm15427	17:31152751-31165053	19.2857	121.177	2.65152	5.25498	5.00E-05	5.91E-03	Up	unknown	unknown	unknown	unknown
SWM	XLOC_038471	Bhlhe40	11:61505143-61512931	1.73843	11.1536	2.68165	1.44537	5.00E-05	5.91E-03	Up	Class E basic helix-loop-helix protein 40	circadian regulation of gene expression	nucleus	E-box binding
SWM	XLOC_032868	Mup18	4:61435789-61439743	20.1632	134.441	2.73718	3.70194	5.00E-05	5.91E-03	Up	Major urinary protein 18	behavior	extracellular region	pheromone binding
SWM	XLOC_017264	AC158777.2	15:82687724-82707357	11.227	95.8239	3.09341	3.19919	5.00E-05	5.91E-03	Up	unknown	unknown	unknown	unknown
SWM	XLOC_004637	Csrp2	5:113075838-113081584	2.42958	24.732	3.3476	1.93201	5.00E-05	5.91E-03	Up	Cysteine and glycine-rich protein 2 (Fragment)	unknown	unknown	metal ion binding
SWM	XLOC_047600	Adat1	8:111966809-111992302	1.15568	13.5883	3.55555	3.32278	5.00E-05	5.91E-03	Up	tRNA-specific adenosine deaminase 1 (mADAT1) (EC 3.5.4.34) (tRNA-specific adenosine-37 deaminase)	tRNA processing	unknown	metal ion binding
SWM	XLOC_022691	Gm4950	5:148871583-149015813	1.13629	24.9011	4.4538	2.16394	5.00E-05	5.91E-03	Up	Proteasome subunit beta type (EC 3.4.25.1)	proteasome-mediated ubiquitin-dependent p	cytoplasm	threonine-type endopeptidase activity
SWM	XLOC_050062	Rn7sk	1:190117767-190219846	10.9942	266.797	4.60093	4.45315	5.00E-05	5.91E-03	Up	unknown	unknown	unknown	unknown
SWM	XLOC_008666	Ints2	5:136908149-136918905	11.6973	352.617	4.91385	6.34443	5.00E-05	5.91E-03	Up	Integrator complex subunit 2 (Int2)	snRNA 3'-end processing	cytoplasm	unknown
SWM	XLOC_045957	Tmem192	1:127205014-127774092	2.09566	80.2555	5.25912	4.04201	5.00E-05	5.91E-03	Up	Transmembrane protein 192 (Fragment)	unknown	integral component of membrane	unknown
SWM	XLOC_032180	Sdhb	8:89027234-89068283	5.05356	216.928	5.42377	3.95296	5.00E-05	5.91E-03	Up	Succinate dehydrogenase [ubiquinone] iron-sulfur subunit, mitochondrial (EC 1.3.5.1) (Iron-sulfur subunit of c	aerobic respiration	mitochondrial inner membrane	2 iron, 2 sulfur cluster binding
SWM	XLOC_020793	Kat2b	9:118606689-119043998	2.18445	97.1023	5.47417	3.89022	5.00E-05	5.91E-03	Up	Histone acetyltransferase KAT2B (EC 2.3.1.48) (Histone acetyltransferase PCAF) (Histone acetylase PCAF) (Ly	cell cycle	A band	acetyltransferase activity
SWM	XLOC_021572	Slc22a7	10:89574019-89632022	1.08835	83.4606	6.26088	5.05567	5.00E-05	5.91E-03	Up	Solute carrier family 22 member 7	unknown	integral component of membrane	organic anion transmembrane transporter activity
SWM	XLOC_034328	Tacc3	16:36156810-36161948	0	4.95788	inf	nan	5.00E-05	5.91E-03	Up	Transforming acidic coiled-coil-containing protein 3 (ARNT-interacting protein)	astral microtubule organization	centrosome	protein domain specific binding
SWM	XLOC_021670	2410015M20	6:125083436-125084029	0	2.45882	inf	nan	5.00E-05	5.91E-03	Up	Sr104 protein (Fragment)	unknown	unknown	unknown
SWM	XLOC_027946	-	1:49555658-49555858	0	33.3146	inf	nan	5.00E-05	5.91E-03	Up	unknown	unknown	unknown	unknown
SWM	XLOC_037319	-	4:86670282-86670417	0	342.137	inf	nan	5.00E-05	5.91E-03	Up	unknown	unknown	unknown	unknown
SWM	XLOC_047692	-	8:47701637-47706774	0	7.98059	inf	nan	5.00E-05	5.91E-03	Up	unknown	unknown	unknown	unknown
SWM	XLOC_001495	-	8:18845941-18846120	0	76.7844	inf	nan	5.00E-05	5.91E-03	Up	unknown	unknown	unknown	unknown
SWM	XLOC_003528	-	1:105114691-105114848	0	104.561	inf	nan	5.00E-05	5.91E-03	Up	unknown	unknown	unknown	unknown
SWM	XLOC_011810	-	10:40175629-40175761	0	445.59	inf	nan	5.00E-05	5.91E-03	Up	unknown	unknown	unknown	unknown
SWM	XLOC_016884	-	12:108366893-108367033	0	280.071	inf	nan	5.00E-05	5.91E-03	Up	unknown	unknown	unknown	unknown
SWM	XLOC_032459	Gm11808	9:96895657-96896147	0	3.64262	inf	nan	5.00E-05	5.91E-03	Up	unknown	unknown	unknown	unknown
SWM	XLOC_020467	Gm15318	X:8042942-8074760	0	1.81994	inf	nan	5.00E-05	5.91E-03	Up	unknown	unknown	unknown	unknown
SWM	XLOC_003938	Gpx4-ps2	16:50665007-50666346	0	1.00234	inf	nan	5.00E-05	5.91E-03	Up	unknown	unknown	unknown	unknown
SWM	XLOC_006694	Hint1	5:34007178-34187834	1.71874	0.112112	-3.93833	-0.295492	1.00E-04	0.01	Down	Histidine triad nucleotide-binding protein 1 (EC 3.-.-.-) (Adenosine 5'-monophosphoramidase) (Protein kinase	intrinsic apoptotic signaling pathway by p53 c	cytosol	hydrolase activity
SWM	XLOC_008915	Acly	11:100476352-100528000	75.412	35.0778	-1.10424	-1.72448	1.00E-04	0.01	Down	ATP-citrate synthase (EC 2.3.3.8) (ATP-citrate (pro-S-)-lyase) (Citrate cleavage enzyme)	acetyl-CoA biosynthetic process	cytoplasm	ATP binding
SWM	XLOC_007160	Wfdc21	6:72385216-72390703	201.077	424.163	1.07687	1.89358	1.00E-04	0.01	Up	Protein Wfdc21 (Wdnm1-like protein)	response to lipopolysaccharide	extracellular space	enzyme activator activity
SWM	XLOC_026473	Rpl35	6:115774537-115808747	749.218	1612.32	1.10568	2.00251	1.00E-04	0.01	Up	60S ribosomal protein L35	maturation of LSU-rRNA from tricistronic rRN	cytosolic large ribosomal subunit	mRNA binding
SWM	XLOC_019847	Gm9575	X:152908777-152910296	58.6668	128.206	1.12785	1.78258	1.00E-04	0.01	Up	unknown	unknown	unknown	unknown
SWM	XLOC_035453	Ift22	13:58157594-58164693	74.1863	163.205	1.13746	1.90452	1.00E-04	0.01	Up	Intraflagellar transport protein 22 homolog (Fragment)	unknown	centrosome	unknown
SWM	XLOC_007840	Sec61g	4:140961202-140979193	323.696	719.752	1.15286	1.94941	1.00E-04	0.01	Up	Protein transport protein Sec61 subunit gamma	protein targeting	endoplasmic reticulum membrane	P-P-bond-hydrolysis-driven protein transmembrane transporter activity
SWM	XLOC_006105	Hsd17b6	X:152001844-152004442	236.747	531.079	1.16558	2.00227	1.00E-04	0.01	Up	17-beta-hydroxysteroid dehydrogenase type 6 (17-beta-HSD 6) (17-beta-HSD6) (EC 1.1.1.105) (EC 1.1.1.239)	steroid metabolic process	early endosome membrane	estradiol 17-beta-dehydrogenase activity
SWM	XLOC_033437	Eif3i	3:89259357-89267079	118.899	268.847	1.17704	1.88353	1.00E-04	0.01	Up	Eukaryotic translation initiation factor 3 subunit I (eIF3i) (Eukaryotic translation initiation factor 3 subunit 2)	formation of cytoplasmic translation initiatio	eukaryotic 43S preinitiation complex	translation initiation factor activity
SWM	XLOC_031095	Usp45	19:47067604-47090625	233.624	574.259	1.29751	2.12385	1.00E-04	0.01	Up	Ubiquitin carboxyl-terminal hydrolase 45 (Fragment)	protein deubiquitination	unknown	thiol-dependent ubiquitinyl hydrolase activity
SWM	XLOC_036002	Haus3	11:59183856-59192212	25.8127	63.6594	1.30229	1.86318	1.00E-04	0.01	Up	HAUS augmin-like complex subunit 3	cell division	centrosome	unknown
SWM	XLOC_047178	Rpl18a	4:136028264-136053428	651.59	1623.81	1.31735	2.10346	1.00E-04	0.01	Up	60S ribosomal protein L18a	cytoplasmic translation	cytosolic large ribosomal subunit	RNA binding
SWM	XLOC_049693	Atp5l	5:145183697-145192062	274.537	696.407	1.34293	2.13398	1.00E-04	0.01	Up	ATP synthase subunit g, mitochondrial (ATPase subunit g)	ATP metabolic process	mitochondrial inner membrane	proton-exporting ATPase activity, phosphorylative mechanism
SWM	XLOC_031460	Orm1	7:125467639-125491596	18.6564	47.4575	1.34696	1.98907	1.00E-04	0.01	Up	Alpha-1-acid glycoprotein	regulation of immune system process	extracellular space	unknown
SWM	XLOC_051132	Slc25a14	17:46432184-46438839	44.0917	113.696	1.3666	2.09321	1.00E-04	0.01	Up	Brain mitochondrial carrier protein 1	mitochondrial transport	integral component of membrane	unknown
SWM	XLOC_021254	Rps10	10:128548113-128549305	2111.29	5681.71	1.4282	2.09877	1.00E-04	0.01	Up	40S ribosomal protein S10	ribosomal small subunit assembly	cytosol	RNA binding
SWM	XLOC_045130	Fuom	7:45125443-45136432	1582.51	4963.97	1.64928	2.26119	1.00E-04	0.01	Up	Fucose mutarotase (Fragment)	monosaccharide metabolic process	unknown	isomerase activity
SWM	XLOC_018449	AC118710.5	15:75427604-75431829	139.474	304.185	1.12495	1.82258	1.50E-04	0.01	Up	unknown	unknown	unknown	unknown
SWM	XLOC_046257	Sall1	3:93555079-93564643	227.4	504.787	1.15044	1.93055	1.50E-04	0.01	Up	Sal-like 1 (Drosophila) (Sal-like protein 1)	adrenal gland development	chromocenter	beta-catenin binding
SWM	XLOC_022009	Greb1l	15:75596627-75599481	38.3697	96.1514	1.32534	1.9403	1.50E-04	0.01	Up	GREB1-like protein	branching involved in ureteric bud morphoger	integral component of membrane	unknown
SWM	XLOC_007740	Gm27517	12:54696781-54696945	274.672	1247.73	2.18353	8.23066	1.50E-04	0.01	Up	unknown	unknown	unknown	unknown
SWM	XLOC_038562	8430408G22	17:56607451-56609771	143.538	308.578	1.1042	1.90867	2.00E-04	0.02	Up	RIKEN cDNA 8430408G22 gene (Fragment)	unknown	unknown	unknown
SWM	XLOC_033233	Uqcrh	17:27122662-27133916	130.375	285.848	1.13258	1.88208	2.00E-04	0.02	Up	Uncharacterized protein (Fragment)	mitochondrial electron transport, ubiquinol tc	mitochondrion	ubiquinol-cytochrome-c reductase activity
SWM	XLOC_028242	Spata5	7:19149721-19153542	31.5164	69.1703	1.13405	1.9132	2.00E-04	0.02	Up	Spermatogenesis-associated protein 5 (Spermatogenesis-associated factor protein)	brain development	cytoplasm	ATP binding
SWM	XLOC_017994	Rpl30	11:78176710-78183584	381.225	840.956	1.14139	1.83288	2.00E-04	0.02	Up	60S ribosomal protein L30	antimicrobial humoral immune response mec	cytosolic large ribosomal subunit	RNA binding
SWM	XLOC_008219	Guk1	12:87106860-87266272	628.971	1601.89	1.34871	1.89305	2.00E-04	0.02	Up	Guanylate kinase	ATP metabolic process	cytosol	guanylate kinase activity
SWM	XLOC_050093	Cox7a2	3:57644348-57651715	13.3958	38.1704	1.51068	1.84229	2.00E-04	0.02	Up	Cytochrome c oxidase subunit 7A2, mitochondrial (Cytochrome c oxidase subunit VIIa-liver/heart) (Cytochron	unknown	mitochondrial inner membrane	cytochrome-c oxidase activity
SWM	XLOC_003491	-	1:172990330-172998325	1.37385	224.804	7.3543	5.04905	2.00E-04	0.02	Up	unknown	unknown	unknown	unknown
SWM	XLOC_042838	Zfp787	2:170108642-170148103	2.08352	0.34853	-2.57967	-1.11419	2.50E-04	0.02	Down	Zinc finger protein 787 (Fragment)	unknown	unknown	nucleic acid binding
SWM	XLOC_021481	Ppp1r11	4:105157202-105232764	26.3918	12.3983	-1.08995	-1.7781	2.50E-04	0.02	Down	E3 ubiquitin-protein ligase PPP1R11 (EC 2.3.2.27) (Protein phosphatase 1 regulatory subunit 11) (T-complex t	defense response to Gram-positive bacterium	cytoplasm	protein phosphatase 1 binding
SWM	XLOC_024430	Psd4	X:155323917-155340754	136.653	275.176	1.00984	1.81781	2.50E-04	0.02	Up	PH and SEC7 domain-containing protein 4 (Exchange factor for ADP-ribosylation factor guanine nucleotide fa	regulation of ARF protein signal transduction	ruffle membrane	ARF guanyl-nucleotide exchange factor activity
SWM	XLOC_051984	Hsd17b10	4:43654125-43656466	176.467	360.049	1.02879	1.79708	2.50E-04	0.02	Up	3-hydroxyacyl-CoA dehydrogenase type-2 (EC 1.1.1.35) (17-beta-hydroxysteroid dehydrogenase 10) (17-beta-	mitochondrial tRNA 3'-end processing	endoplasmic reticulum	3-hydroxy-2-methylbutyryl-CoA dehydrogenase activity
SWM	XLOC_019279	Cpox	9:79598990-79759878	219.07	462.621	1.07844	1.88059	2.50E-04	0.02	Up	Oxygen-dependent coproporphyrinogen-III oxidase, mitochondrial (COX) (Coprogen oxidase) (Coproporphyrin	heme biosynthetic process	cytosol	coproporphyrinogen oxidase activity
SWM	XLOC_045384	Pet100	4:63344453-63353952	644.457	1363.06	1.0807	1.84322	2.50E-04	0.02	Up	Protein PET100 homolog, mitochondrial	mitochondrial respiratory chain complex IV as	integral component of mitochondrial	unfolded protein binding
SWM	XLOC_032862	Gm21320	13:28257527-28258134	81.6221	174.827	1.0989	1.81621	2.50E-04	0.02	Up	Major urinary protein 1	unknown	unknown	small molecule binding
SWM	XLOC_018316	AL772386.1	15:58888942-58939488	506.038	1121.77	1.14847	1.92354	2.50E-04	0.02	Up	unknown	unknown	unknown	unknown
SWM	XLOC_037660	Adck2	6:39573872-39725463	166.385	388.878	1.22479	1.84968	2.50E-04	0.02	Up	Uncharacterized aarF domain-containing protein kinase 2 (EC 2.7.11.-)	unknown	integral component of membrane	ATP binding
SWM	XLOC_014105	Gm10260	7:137437613-137468594	32.0139	80.291	1.32654	1.78833	2.50E-04	0.02	Up	Uncharacterized protein	ribosome biogenesis	cytosol	RNA binding
SWM	XLOC_020425	Rpl10a	14:30654359-30691317	4.93868	13.1774	1.41587	1.87394	2.50E-04	0.02	Up	Ribosomal protein	translation	large ribosomal subunit	RNA binding
SWM	XLOC_036727	Crybb3	16:58669913-58680391	12.3	3.89098	-1.66045	-1.90196	3.00E-04	0.02	Down	Beta-crystallin B3 (Fragment)	visual perception	unknown	structural constituent of eye lens
SWM	XLOC_007437	Rdm1	9:98422960-98446575	24.778	51.7557	1.06266	1.7975	3.00E-04	0.02	Up	RAD52 motif-containing protein 1 (Fragment)	DNA recombination	unknown	unknown
SWM	XLOC_007256	Tob1	11:69838513-69839618	328.715	689.435	1.06858	1.86975	3.00E-04	0.02	Up	Uncharacterized protein	negative regulation of cell proliferation	unknown	unknown
SWM	XLOC_037192	Atp5j2	2:10056015-10080510	266.47	563.612	1.08073	1.85191	3.00E-04	0.02	Up	ATP synthase subunit f, mitochondrial	ATP biosynthetic process	integral component of membrane	ATPase activity
SWM	XLOC_048263	Rps25	17:33951998-33967036	460.73	1031.51	1.16277	1.89807	3.00E-04	0.02	Up	40S ribosomal protein S25	ribosomal small subunit assembly	cytosolic small ribosomal subunit	structural constituent of ribosome
SWM	XLOC_027627	Mafb	10:117277330-117282321	21.6167	49.7655	1.203	1.85004	3.00E-04	0.02	Up	Transcription factor MafB (Maf-B) (Kreisler) (Segmentation protein Kr) (Transcription factor Maf-1) (V-maf i	abducens nerve formation	nucleoplasm	DNA binding
SWM	XLOC_028143	Nceh1	16:15848440-15863369	19.5808	47.1865	1.26893	1.83437	3.00E-04	0.02	Up	Neutral cholesterol ester hydrolase 1	protein dephosphorylation	membrane	phosphate ion binding
SWM	XLOC_045749	Ppp1r3b	17:36942903-36951741	83.9312	223.049	1.41008	1.90339	3.00E-04	0.02	Up	Protein phosphatase 1 regulatory subunit 3B (Hepatic glycogen-targeting protein phosphatase 1 regulatory s	glycogen metabolic process	glycogen granule	enzyme binding
SWM	XLOC_002726	Tmem163	11:76406951-76416292	16.1913	61.7572	1.93139	1.6499	3.00E-04	0.02	Up	Transmembrane protein 163 (Synaptic vesicle membrane protein of 31 kDa)	unknown	cell junction	zinc ion binding
SWM	XLOC_029004	Gstm5	18:10325176-10562934	0.118304	2.4567	4.37615	0.518082	3.00E-04	0.02	Up	Glutathione S-transferase Mu 5	metabolic process	sperm fibrous sheath	glutathione transferase activity
SWM	XLOC_040912	Snrpd2	12:108836020-108856815	304.93	603.554	0.985006	1.72228	3.50E-04	0.03	Up	Small nuclear ribonucleoprotein Sm D2 (Sm-D2) (snRNP core protein D2)	spliceosomal snRNP assembly	catalytic step 2 spliceosome	U1 snRNP binding
SWM	XLOC_044978	Dctpp1	2:148871721-148875692	157.726	329.906	1.06463	1.75997	3.50E-04	0.03	Up	dCTP pyrophosphatase 1 (EC 3.6.1.12) (Deoxycytidine-triphosphatase 1) (dCTPase 1) (RS21-C6)	dCTP catabolic process	cytosol	dCTP diphosphatase activity
SWM	XLOC_048834	Gm10123	18:75005859-75009935	60.7098	130.422	1.10318	1.83432	3.50E-04	0.03	Up	unknown	unknown	unknown	unknown
SWM	XLOC_032425	Mrpl20	11:120484612-120489065	191.741	421.3	1.13569	1.82527	3.50E-04	0.03	Up	Mrpl20 protein	translation	ribosome	rRNA binding
SWM	XLOC_012473	Idnk	10:127990852-128007903	395.668	919.901	1.21719	1.62338	3.50E-04	0.03	Up	Probable gluconokinase (EC 2.7.1.12) (Gluconate kinase)	D-gluconate catabolic process	unknown	ATP binding
SWM	XLOC_007336	Psmb3	17:15460497-15498301	172.68	447.593	1.37409	1.89122	3.50E-04	0.03	Up	Proteasome subunit beta type-3 (EC 3.4.25.1) (Proteasome chain 13) (Proteasome component C10-II) (Protea	proteasome-mediated ubiquitin-dependent p	cytosol	endopeptidase activity
SWM	XLOC_027845	Psma7	2:24367579-24414954	6.57771	1.11554	-2.55985	-1.81094	4.00E-04	0.03	Down	Proteasome subunit alpha type-7 (EC 3.4.25.1) (Proteasome subunit RC6-1)	ubiquitin-dependent protein catabolic proces	cytosol	identical protein binding
SWM	XLOC_051337	Ssr4	1:88406960-88426577	206.107	417.703	1.01908	1.76926	4.00E-04	0.03	Up	Translocon-associated protein subunit delta (TRAP-delta) (Signal sequence receptor subunit delta) (SSR-delt	unknown	integral component of membrane	unknown
SWM	XLOC_025502	Atrn	9:44912556-44921489	359.507	733.524	1.02882	1.79117	4.00E-04	0.03	Up	Attractin	unknown	integral component of membrane	unknown
SWM	XLOC_029832	Commd2	8:3707063-3739044	56.2955	116.186	1.04534	1.75942	4.00E-04	0.03	Up	COMM domain-containing protein 2	regulation of transcription, DNA-templated	cytoplasm	unknown
SWM	XLOC_021347	Rps18	7:81342731-81345254	654.602	1353.22	1.04771	1.81989	4.00E-04	0.03	Up	40S ribosomal protein S18 (Ke-3) (Ke3)	ribosome biogenesis	cytosol	protein kinase binding
SWM	XLOC_043187	Apoc2	7:19668096-19681576	5438.52	15105.4	1.47378	1.89623	4.00E-04	0.03	Up	Apolipoprotein C-II (Apo-CII) (ApoC-II) (Apolipoprotein C2)	cholesterol efflux	chylomicron	lipase inhibitor activity
NALB	XLOC_042306	Ampd3	7:110766676-110844457	8.74551	0.0958994	-6.51088	-0.431011	5.00E-05	0.03	Down	AMP deaminase 3 (EC 3.5.4.6) (AMP deaminase H-type) (AMP deaminase isoform E) (Heart-type AMPD)	ADP metabolic process	unknown	AMP deaminase activity
NALB	XLOC_028270	Larp1b	3:40949834-41040234	3.03979	0.0948926	-5.00153	-0.315235	5.00E-05	0.03	Down	La ribonucleoprotein domain family, member 1B	unknown	unknown	RNA binding
NALB	XLOC_000446	Carf	1:60069784-60153953	1.22639	0.142422	-3.10617	-0.476699	5.00E-05	0.03	Down	Calcium-responsive transcription factor (ECBRC-FC1)	cellular response to potassium ion	nucleolus	RNA polymerase II proximal promoter sequence-specific DNA binding
NALB	XLOC_003819	Sult3a1	10:33851034-33879557	33.9722	4.78347	-2.82823	-2.68461	5.00E-05	0.03	Down	Amine sulfotransferase (EC 2.8.2.3) (SULT-X2) (Sulfotransferase 3A1) (ST3A1)	sulfation	cytoplasm	amine sulfotransferase activity
NALB	XLOC_027967	-	2:147936781-147942965	4.08209	0.646196	-2.65926	-2.18222	5.00E-05	0.03	Down	unknown	unknown	unknown	unknown
NALB	XLOC_023566	Neat1	19:5817196-5845554	75.0545	13.3262	-2.49367	-2.46453	5.00E-05	0.03	Down	unknown	unknown	unknown	unknown
NALB	XLOC_013346	Carmil1	13:24012343-24280795	3.29912	0.725186	-2.18566	-0.998317	5.00E-05	0.03	Down	F-actin-uncapping protein LRRC16A	barbed-end actin filament uncapping	lamellipodium	unknown
NALB	XLOC_009161	Socs3	11:117966078-117970047	8.53324	1.88917	-2.17534	-1.87857	5.00E-05	0.03	Down	Uncharacterized protein	JAK-STAT cascade	intracellular	unknown
NALB	XLOC_018774	-	15:75214574-75215317	1.04784	0	NA	nan	5.00E-05	0.03	Down	unknown	unknown	unknown	unknown
NALB	XLOC_022525	-	18:20933547-20935222	5.32172	0	NA	nan	5.00E-05	0.03	Down	unknown	unknown	unknown	unknown
NALB	XLOC_033976	-	4:150707642-150708195	1.75879	0	NA	nan	5.00E-05	0.03	Down	unknown	unknown	unknown	unknown
NALB	XLOC_039187	-	6:35848047-35864211	4.06048	0	NA	nan	5.00E-05	0.03	Down	unknown	unknown	unknown	unknown
NALB	XLOC_040587	-	6:148637430-148637996	1.17531	0	NA	nan	5.00E-05	0.03	Down	unknown	unknown	unknown	unknown
NALB	XLOC_045256	-	7:14403590-14404300	1.08034	0	NA	nan	5.00E-05	0.03	Down	unknown	unknown	unknown	unknown
NALB	XLOC_045338	-	7:106619579-106620440	1.41315	0	NA	nan	5.00E-05	0.03	Down	unknown	unknown	unknown	unknown
NALB	XLOC_006874	Rpl26	11:68901582-68906989	135.272	419.111	1.63147	1.99441	5.00E-05	0.03	Up	60S ribosomal protein L26	translation	large ribosomal subunit	structural constituent of ribosome
NALB	XLOC_010279	Cyp46a1	12:108334380-108362338	38.2233	124.461	1.70317	2.25732	5.00E-05	0.03	Up	Cholesterol 24-hydroxylase (Fragment)	cholesterol catabolic process	unknown	heme binding
NALB	XLOC_021857	-	17:5492009-5492371	0	2.88395	inf	nan	5.00E-05	0.03	Up	unknown	unknown	unknown	unknown
NALB	XLOC_014326	-	13:37539625-37539944	0	5.55185	inf	nan	5.00E-05	0.03	Up	unknown	unknown	unknown	unknown
NALB	XLOC_003058	Gm15429	1:161726241-161727489	0	2.56526	inf	nan	5.00E-05	0.03	Up	unknown	unknown	unknown	unknown
NALB	XLOC_045342	-	7:110648596-110648781	0	28.406	inf	nan	5.00E-05	0.03	Up	unknown	unknown	unknown	unknown
SWM	XLOC_021247	Uqcc2	1:57388236-57417953	2.33211	0.325196	-2.84225	-0.705679	4.50E-04	0.03	Down	Ubiquinol-cytochrome-c reductase complex assembly factor 2	mitochondrial respiratory chain complex III as	mitochondrial inner membrane	unknown
SWM	XLOC_014766	Mbl1	2:160363702-160367065	8.07645	2.89733	-1.479	-1.99424	4.50E-04	0.03	Down	Mannose-binding protein A (MBP-A) (Mannan-binding protein) (Ra-reactive factor polysaccharide-binding cc	complement activation, classical pathway	collagen trimer	calcium-dependent protein binding
SWM	XLOC_047518	Agrp	8:105566323-105682922	1.83011	0	NA	nan	4.50E-04	0.03	Down	Agouti-related protein (Fragment)	adult feeding behavior	extracellular region	neuropeptide hormone activity
SWM	XLOC_032712	Hint2	11:54866382-54870501	421.626	852.732	1.01613	1.76841	4.50E-04	0.03	Up	Histidine triad nucleotide-binding protein 2, mitochondrial (HINT-2) (EC 3.-.-.-) (HINT-3)	apoptotic process	mitochondrion	hydrolase activity
SWM	XLOC_006936	Psmb6	14:54609318-54618022	79.2541	174.833	1.14142	1.67858	4.50E-04	0.03	Up	Proteasome subunit beta type-6 (EC 3.4.25.1) (Low molecular mass protein 19) (Macropain delta chain) (Mu	proteolysis involved in cellular protein catabo	cytosol	cadherin binding
SWM	XLOC_049403	Tmem205	8:64945589-64969036	19.1097	45.1035	1.23894	1.75531	4.50E-04	0.03	Up	Transmembrane protein 205 (Fragment)	unknown	integral component of membrane	unknown
SWM	XLOC_044930	Nsmce1	9:109832748-109843290	46.09	112.389	1.28598	1.84584	4.50E-04	0.03	Up	Non-structural maintenance of chromosomes element 1 homolog (Fragment)	DNA repair	Smc5-Smc6 complex	unknown
SWM	XLOC_037245	Gm42791	4:60817588-60821463	9.31844	30.2652	1.6995	1.94697	4.50E-04	0.03	Up	Uncharacterized protein (Fragment)	unknown	unknown	unknown
SWM	XLOC_032734	Tomm5	11:94211453-94215495	12.4472	4.66849	-1.4148	-1.86856	5.00E-04	0.03	Down	Mitochondrial import receptor subunit TOM5 homolog	protein targeting to mitochondrion	integral component of membrane	unknown
SWM	XLOC_010065	Gstz1	19:4035405-4046006	125.317	265.042	1.08064	1.86649	5.00E-04	0.03	Up	Maleylacetoacetate isomerase (MAAI) (EC 5.2.1.2) (GSTZ1-1) (Glutathione S-transferase zeta 1) (EC 2.5.1.18	glutathione metabolic process	mitochondrion	glutathione transferase activity
SWM	XLOC_034000	Gm15772	1:95498620-95499151	80.0287	178.95	1.16096	1.82796	5.00E-04	0.03	Up	unknown	unknown	unknown	unknown
SWM	XLOC_050897	Mid1ip1	2:71453275-71537891	12.8178	30.8731	1.26821	1.82978	5.00E-04	0.03	Up	Mid1-interacting protein 1 (Gastrulation-specific G12-like protein) (Mid1-interacting G12-like protein) (Prote	lipid metabolic process	cytoplasm	identical protein binding
SWM	XLOC_051783	Rpl36a	17:56613169-56626927	1496.41	3729.34	1.31741	1.88239	5.00E-04	0.03	Up	60S ribosomal protein L36a	translation	ribosome	structural constituent of ribosome
SWM	XLOC_032865	Mup15	5:100798626-100804589	22.1474	57.7352	1.38231	1.85759	5.00E-04	0.03	Up	Major urinary protein 15	unknown	unknown	small molecule binding
SWM	XLOC_047929	Pin1	8:3621547-3625848	96.7577	271.279	1.48733	1.70627	5.00E-04	0.03	Up	Peptidyl-prolyl cis-trans isomerase NIMA-interacting 1 (EC 5.2.1.8) (Peptidyl-prolyl cis-trans isomerase Pin1)	cell cycle	cytoplasm	beta-catenin binding
SWM	XLOC_050854	Gm14820	11:3517522-3540612	0.0171814	1.05483	5.94001	0.182076	5.00E-04	0.03	Up	unknown	unknown	unknown	unknown
SWM	XLOC_039650	Vamp8	4:21727700-21837872	0.239882	17.1138	6.15669	1.54679	5.00E-04	0.03	Up	Vesicle-associated membrane protein 8	vesicle-mediated transport	integral component of membrane	unknown
SWM	XLOC_023504	Ndufs8	8:83566670-83571626	327.943	643.66	0.972855	1.71888	5.50E-04	0.04	Up	NADH dehydrogenase [ubiquinone] iron-sulfur protein 8, mitochondrial (EC 1.6.5.3) (EC 1.6.99.3) (Complex I-	mitochondrial respiratory chain complex I ass	mitochondrial respiratory chain comp	4 iron, 4 sulfur cluster binding
SWM	XLOC_046580	Gm21399	1:166431704-166431875	2277.68	4766.93	1.06549	1.79645	5.50E-04	0.04	Up	unknown	unknown	unknown	unknown
SWM	XLOC_038677	2010008C14I	4:107889812-107899384	44.2561	106.518	1.26715	1.70686	5.50E-04	0.04	Up	unknown	unknown	unknown	unknown
SWM	XLOC_014603	Anxa11	14:25841827-25886931	13.39	4.61486	-1.5368	-1.7569	6.00E-04	0.04	Down	Annexin	unknown	cytoplasm	calcium-dependent phospholipid binding
SWM	XLOC_006909	Tmem256	8:105326353-105347953	50.0982	107.197	1.09744	1.72782	6.00E-04	0.04	Up	Transmembrane protein 256	unknown	integral component of membrane	unknown
SWM	XLOC_031709	Plpp3	9:20652094-20666584	5.80706	12.7528	1.13493	1.76243	6.00E-04	0.04	Up	Phospholipid phosphatase 3 (EC 3.1.3.4) (Lipid phosphate phosphohydrolase 3) (PAP2-beta) (Phosphatidate p	Bergmann glial cell differentiation	endoplasmic reticulum membrane	integrin binding
SWM	XLOC_044621	Hbb-bt	10:45875290-45876086	51.087	114.872	1.169	1.76843	6.00E-04	0.04	Up	unknown	unknown	unknown	unknown
SWM	XLOC_026117	Atp5c1	6:124739182-124756524	290.945	703.705	1.27422	1.78537	6.00E-04	0.04	Up	ATP synthase subunit gamma	ATP synthesis coupled proton transport	myelin sheath	proton-transporting ATP synthase activity, rotational mechanism
SWM	XLOC_005763	Slc17a8	12:104314552-104414338	3291.17	11268.9	1.77568	1.5156	6.00E-04	0.04	Up	Vesicular glutamate transporter 3	unknown	integral component of membrane	L-glutamate transmembrane transporter activity
SWM	XLOC_027745	Zfp217	8:40423814-40634797	0.0539578	3.18596	5.88375	0.385705	6.00E-04	0.04	Up	Zfp217 protein (Fragment)	cell aging	histone deacetylase complex	RNA polymerase II proximal promoter sequence-specific DNA binding
SWM	XLOC_010175	Ttc8	15:75924675-75929832	39.3685	79.2619	1.00959	1.64283	6.50E-04	0.04	Up	Tetratricopeptide repeat protein 8 (Fragment)	non-motile cilium assembly	BBSome	unknown
SWM	XLOC_032671	Enho	16:23107443-23127742	37.4561	75.7228	1.01553	2.39844	6.50E-04	0.04	Up	Adropin (Energy homeostasis-associated protein)	positive regulation of Notch signaling pathwa	extracellular region	unknown
SWM	XLOC_022392	BC031181	15:77369493-77376925	22.8059	92.6589	2.02252	2.25699	6.50E-04	0.04	Up	unknown	unknown	unknown	unknown
SWM	XLOC_043722	Flt3l	19:6057887-6059524	692.145	1378.83	0.994301	1.71779	7.00E-04	0.04	Up	FMS-like tyrosine kinase 3 ligand, isoform CRA_g	embryonic hemopoiesis	integral component of membrane	cytokine activity
SWM	XLOC_034929	Mrps18c	7:142532685-142540747	90.8077	190.217	1.06676	1.73124	7.00E-04	0.04	Up	28S ribosomal protein S18c, mitochondrial (MRP-S18-c) (Mrps18-c) (S18mt-c) (28S ribosomal protein S18-1,	mitochondrial translation	cytosolic small ribosomal subunit	structural constituent of ribosome
SWM	XLOC_046592	Clec4g	11:120710941-120713756	74.5929	164.511	1.14107	1.73281	7.00E-04	0.04	Up	C-type lectin domain family 4 member G	negative regulation of T cell mediated immu	integral component of membrane	polysaccharide binding
SWM	XLOC_017380	Gpihbp1	18:51865263-51865881	26.867	65.0326	1.27533	1.73554	7.00E-04	0.04	Up	Glycosylphosphatidylinositol-anchored high density lipoprotein-binding protein 1 (GPI-HBP1) (GPI-anchored H	cholesterol homeostasis	anchored component of plasma mem	chylomicron binding
SWM	XLOC_042369	Rexo5	11:101627194-101636100	17.9566	51.057	1.5076	1.79397	7.00E-04	0.04	Up	RNA exonuclease 5 short isoform (Fragment)	unknown	nucleolus	exonuclease activity
SWM	XLOC_049134	Itga9	11:86201360-86357602	2.61583	0.218142	-3.58393	-0.864545	7.50E-04	0.05	Down	Integrin alpha 9 protein	cell adhesion	alpha9-beta1 integrin-ADAM8 comple	unknown
SWM	XLOC_046710	Tdrp	5:119570129-119684724	11.5896	1.07202	-3.43442	-1.81955	7.50E-04	0.05	Down	Testis development-related protein	spermatogenesis	cytoplasm	unknown
SWM	XLOC_007042	Timm22	8:13952007-13975032	3.37096	0.67684	-2.31627	-1.20591	7.50E-04	0.05	Down	Mitochondrial import inner membrane translocase subunit Tim22	protein import into mitochondrial inner mem	integral component of membrane	mitochondrion targeting sequence binding
SWM	XLOC_042606	Glrx3	14:57069425-57104750	93.8177	46.3058	-1.01867	-1.74769	7.50E-04	0.05	Down	Glutaredoxin-3	cell redox homeostasis	cell	electron transfer activity
SWM	XLOC_021079	Psmb1	2:180036366-180042445	103.174	208.348	1.01392	1.73627	7.50E-04	0.05	Up	Proteasome subunit beta type-1 (EC 3.4.25.1) (Macropain subunit C5) (Multicatalytic endopeptidase complex	proteolysis involved in cellular protein catabo	cytosol	threonine-type endopeptidase activity
SWM	XLOC_035483	Azgp1	2:130906494-131030333	2.54545	1.13519	-1.16499	-1.46848	8.00E-04	0.05	Down	Zinc-alpha-2-glycoprotein (Zn-alpha-2-GP) (Zn-alpha-2-glycoprotein)	cell adhesion	extracellular matrix	unknown
SWM	XLOC_009221	Cenpx	3:87357880-87371073	41.7943	79.1048	0.920458	1.61309	8.00E-04	0.05	Up	Centromere protein X	DNA repair	unknown	unknown
SWM	XLOC_000270	Rpl31	15:34440504-34444812	384.218	792.903	1.04522	1.70888	8.00E-04	0.05	Up	60S ribosomal protein L31	translation	ribosome	structural constituent of ribosome
SWM	XLOC_030150	Rps27	9:44403697-44418569	630.608	1348.08	1.09609	1.7015	8.00E-04	0.05	Up	40S ribosomal protein S27	ribosomal small subunit assembly	cytosolic small ribosomal subunit	metal ion binding
SWM	XLOC_001687	Prox1os	4:88599163-88599832	24.7964	57.7715	1.22023	1.65838	8.00E-04	0.05	Up	unknown	unknown	unknown	unknown
SWM	XLOC_049043	Nme6	12:69296680-69357165	0.177446	2.11875	3.57776	0.695285	8.00E-04	0.05	Up	Nucleoside diphosphate kinase 6 (NDK 6) (NDP kinase 6) (EC 2.7.4.6) (nm23-M6)	CTP biosynthetic process	mitochondrion	ATP binding
SWM	XLOC_044321	Rps17	7:28350651-28353155	841.347	1807.16	1.10295	1.77101	8.50E-04	0.05	Up	40S ribosomal protein S17	erythrocyte homeostasis	cytosolic small ribosomal subunit	structural constituent of ribosome
SWM	XLOC_007346	Rpl19	8:70894721-70897427	1574.18	3653.75	1.21478	1.71146	8.50E-04	0.05	Up	60S ribosomal protein L19	cytoplasmic translation	cytosolic large ribosomal subunit	5.8S rRNA binding
SWM	XLOC_039298	Rarres2	2:34777555-34799912	16.3829	49.2627	1.5883	1.81442	8.50E-04	0.05	Up	Retinoic acid receptor responder protein 2	antifungal humoral response	extracellular matrix	signaling receptor binding

bioRxiv preprint doi: https://doi.org/10.1101/2024.10.28.620613; this version posted October 28, 2024. The copyright holder for this preprint (which was not certified by peer review) is the author/funder, who has granted bioRxiv a license to display the preprint in perpetuity. It is made available under aCC-BY 4.0 International license.

Table S3. The number of microbial metabolites that exhibited a significant increase or decrease in expression upon consumption of 1.5% oxalate by the host.

Microbiome	Molecular mass	Retention time	Electron spra	Ave No Oxala	Ave 1.5% Ox	p-value	FDR	Log2 fold cha	Direction	HMDB identi	KEGG identif	LIPIDMAPS id	BioCyc identi	Consensus_id
NALB	153.131	0.4	Positive	-0.956	-1.073	0	0	-0.169	Down	0/0	0/0	0	0	0
NALB	294.228	1.022	Positive	-0.594	-0.738	0	0	-0.209	Down	0/0	0/0	0	0	0
NALB	125.108	0.288	Positive	-0.545	-0.731	0	0	-0.268	Down	1/1,[Benzyla	0/0	0	0	1/1,[Benzylamine]
NALB	247.144	3.685	Positive	-0.505	-0.872	0	0	-0.53	Down	0/0	0/0	0	0	0
NALB	57.045	0.305	Positive	-0.454	-0.604	0	0	-0.216	Down	0/0	0/0	0	0	0
NALB	152.118	0.286	Positive	-0.445	-0.55	0	0	-0.152	Down	0/0	0/0	0	0	0
NALB	126.103	0.286	Positive	-0.411	-0.835	0	0	-0.611	Down	2/2,[1-Methy	1/2,[C05127:	0	1,[N-METHYI	2/2,[1-Methylhistamine],[3-Methylhistamine]
NALB	588.552	12.079	Positive	-0.34	-0.89	0	0	-0.794	Down	0/0	0/0	0	0	0
NALB	188.512	13.594	Positive	-0.052	-0.369	0	0	-0.457	Down	0/0	0/0	0	0	0
NALB	393.263	5.494	Positive	0.029	-0.379	0	0	-0.589	Down	0/0	0/0	0	0	0
NALB	100.076	9.166	Positive	0.166	-1.101	0	0	-1.828	Down	2/2,[2-Piperi	1/1,[C18796:	0	0	2/2,[2-Piperidinone],[2-Methylfuran]
NALB	387.321	6.581	Positive	0.262	0.031	0	0	-0.334	Down	2/2,[cis-5-Te	0/0	3,{LMFA0103	0	2/2,[cis-5-Tetradecenoylcarnitine],[trans-2-Tetradecenoylcarnitine]
NALB	494.284	3.676	Positive	0.353	0.037	0	0	-0.455	Down	0/0	0/0	0	0	0
NALB	449.362	9.242	Positive	0.628	0.14	0	0	-0.704	Down	0/0	2/2,[C15791:	4,{LMST0103	0	2/2,[C15791: Teasterone],[C15793: Typhasterol]
NALB	328.321	7.731	Positive	0.905	0.191	0	0	-1.03	Down	3/3,[Stearoy	1/1,[C16526:	28,{LMFA080	0	3/3,[Stearoylethanolamide],[N,N-Dimethylsphingosine],[Eicosenoic acid]
NALB	84.058	9.159	Positive	0.977	-0.364	0	0	-1.935	Down	0/0	0/0	0	0	0
NALB	160.097	3.041	Positive	1.049	0.724	0	0	-0.469	Down	6/6,[2-Methy	1/2,[C03087:	2,{LMFA0105	0	6/6,[2-Methylbutyrylglycine],[Isovalerylglycine],[Valerylglycine],[N-Acetylvaline],[3-Dehydrocarnitine],[5-[illegible]
NALB	90.055	0.625	Positive	1.676	1.202	0	0	-0.684	Down	6/6,[Beta-Al	6/7,[C00041:	1,{LMFA0103	5,[B-ALANIN	6/6,[Beta-Alanine],[L-Alanine],[Sarcosine],[D-Alanine],[Pyruvaldehyde],[Malondialdehyde]
NALB	105.043	10.586	Positive	2.553	1.706	0	0	-1.222	Down	0/0	0/0	0	0	0
NALB	86.08	0.319	Negative	-0.821	-0.979	7.76E-04	0.01	-0.227	Down	0/0	0/0	0	0	0
NALB	136.087	0.286	Negative	-0.383	-0.5	7.76E-04	0.01	-0.169	Down	0/0	0/0	0	0	0
NALB	158.118	13.952	Negative	-0.092	-0.236	7.76E-04	0.01	-0.208	Down	0/0	2/2,[C06727:	10,{LMFA010	0	2/2,[C06727: cis-1,2-Dihydro-3-ethylcatechol; cis-2,3-Dihydroxy-2,3-dihydroethylbenzene; cis-3-Ethyl-cycl[illegible]
NALB	138.103	0.286	Negative	-0.06	-0.293	7.76E-04	0.01	-0.336	Down	0/0	0/0	0	0	0
NALB	181.978	13.479	Negative	-0.005	-0.234	7.76E-04	0.01	-0.331	Down	0/0	0/0	0	0	0
NALB	98.072	0.288	Negative	0.08	-0.022	7.76E-04	0.01	-0.147	Down	0/0	0/0	0	0	0
NALB	112.087	0.287	Negative	0.123	-0.045	7.76E-04	0.01	-0.243	Down	1/1,[Histami	1/1,[C00388:	0	1,[HISTAMIN	1/1,[Histamine]
NALB	434.189	1.575	Negative	0.262	-0.162	7.76E-04	0.01	-0.611	Down	0/0	0/0	1,{LMGP020:	0	1,{LMGP02010104: PE(6:0/6:0) < Glycerophosphoethanolamines [GP02] < Glycerophospholipids [GP]}
NALB	167.999	13.495	Negative	0.588	0.432	7.76E-04	0.01	-0.226	Down	0/0	0/0	0	0	0
NALB	404.816	13.496	Negative	0.785	0.411	7.76E-04	0.01	-0.54	Down	0/0	0/0	0	0	0
NALB	104.107	13.145	Negative	-0.415	-0.731	1.78E-03	0.03	-0.456	Down	3/3,[Iso-Vale	0/0	9,{LMFA0500	2,[CPD-7031:	3/3,[Iso-Valeraldehyde],[2-Methyl-butanal],[2-Pentanone]
NALB	436.185	1.566	Positive	-0.383	-0.5	1.78E-03	0.03	-0.169	Down	0/0	0/0	1,{LMFA0109	0	1,{LMFA01090107: 18-bromo-8E,17E19Z-tricosatrien-4,6-diynoic acid < Fatty Acids and Conjugates [FA0[illegible]
NALB	179.129	0.268	Negative	-1.108	-1.45	3.21E-03	0.04	-0.494	Down	0/0	0/0	0	0	0
NALB	594.24	4.775	Negative	-0.411	-0.858	3.21E-03	0.04	-0.645	Down	0/0	0/0	0	0	0
NALB	165.983	13.467	Negative	0.218	0.04	3.21E-03	0.04	-0.257	Down	0/0	0/0	0	0	0
NALB	171.993	13.765	Negative	0.825	0.192	3.21E-03	0.04	-0.913	Down	0/0	0/0	0	0	0
NALB	266.196	1.021	Negative	-0.311	-0.507	4.50E-03	0.05	-0.282	Down	0/0	0/0	0	0	0
NALB	135.102	0.418	Negative	1.637	1.468	4.50E-03	0.05	-0.243	Down	0/0	0/0	0	0	0
NALB	198.128	4.052	Positive	-1.422	-0.836	0	0	0.846	Up	0/0	0/0	0	0	0
NALB	204.967	0.362	Positive	-1.227	-0.86	0	0	0.529	Up	0/0	0/0	0	0	0
NALB	394.225	5.788	Positive	-1.075	-0.568	0	0	0.731	Up	0/0	0/0	0	0	0
NALB	270.03	3.206	Positive	-0.981	-0.774	0	0	0.298	Up	0/0	0/0	0	0	0
NALB	143.06	6.413	Positive	-0.858	0.137	0	0	1.435	Up	0/0	0/0	0	0	0
NALB	352.15	3.172	Positive	-0.817	-0.69	0	0	0.183	Up	1/1,[(S)-Reti	3/3,[C02105:	0	0	1/1,[(S)-Reticuline]
NALB	246.218	2.028	Positive	-0.752	-0.121	0	0	0.91	Up	0/0	0/0	0	0	0
NALB	217.064	1.133	Positive	-0.625	-0.297	0	0	0.472	Up	1/1,[Bisnorb	1/2,[C20384:	0	0	1/1,[Bisnorbiotin]
NALB	284.045	4.131	Positive	-0.608	-0.374	0	0	0.339	Up	0/0	0/0	0	0	0
NALB	461.274	7.024	Positive	-0.598	-0.15	0	0	0.647	Up	0/0	0/0	0	0	0
NALB	469.36	10.492	Positive	-0.511	-0.088	0	0	0.611	Up	0/0	0/0	0	0	0
NALB	329.182	2.667	Positive	-0.496	-0.163	0	0	0.48	Up	0/0	0/0	0	0	0
NALB	325.187	3.594	Positive	-0.483	-0.082	0	0	0.578	Up	0/0	0/0	0	0	0
NALB	146.98	11.57	Positive	-0.158	1.178	0	0	1.926	Up	0/0	0/0	0	0	0
NALB	224.111	1.258	Positive	-0.116	0.538	0	0	0.943	Up	0/0	0/0	0	0	0
NALB	102.067	11.562	Positive	-0.07	0.279	0	0	0.504	Up	1/1,[Imidazo	1/1,[C06195:	0	0	1/1,[Imidazolone]
NALB	127.05	2.332	Positive	-0.06	0.221	0	0	0.406	Up	2/2,[Thymin	2/2,[C00178:	0	2,[4-IMIDAZ(	2/2,[Thymine],[Imidazoleacetic acid]
NALB	214.144	4.055	Positive	-0.056	0.566	0	0	0.899	Up	0/0	0/0	1,{LMFA0803	0	1,{LMFA08030006: N-heptanoyl-homoserine lactone < Fatty amides [FA08] < Fatty Acyls [FA]}
NALB	59.061	0.607	Positive	0.159	1.596	0	0	2.073	Up	0/0	0/0	0	0	0
NALB	144.982	11.585	Positive	0.38	1.719	0	0	1.932	Up	0/0	0/0	0	0	0
NALB	192.159	0.813	Positive	0.399	0.727	0	0	0.473	Up	0/0	0/0	8,{LMFA0105	0	8,{LMFA01050024: 2-hydroxy pelargonic acid < Fatty Acids and Conjugates [FA01] < Fatty Acyls [FA]},{LM[illegible]
NALB	136.022	11.258	Positive	0.4	1.275	0	0	1.262	Up	0/0	0/0	0	0	0
NALB	171.149	13.998	Positive	0.418	0.646	0	0	0.328	Up	0/0	0/0	0	0	0
NALB	215.139	1.856	Positive	0.435	0.848	0	0	0.596	Up	1/2,[Metane	2/2,[C01909:	0	0	1/2,[Metanephrine]
NALB	241.068	0.548	Positive	1.018	1.118	0	0	0.144	Up	0/0	0/0	1,{LMGL0301	0	1,{LMGL03012615: Triacetin < Triradylglycerols [GL03] < Glycerolipids [GL]}
NALB	60.045	11.104	Positive	1.261	2.225	0	0	1.39	Up	0/0	0/0	0	0	0
NALB	445.217	2.368	Negative	-1.306	-0.963	7.76E-04	0.01	0.494	Up	0/0	0/0	0	0	0
NALB	213.179	1.436	Negative	-1.269	-0.585	7.76E-04	0.01	0.986	Up	0/0	0/0	0	0	0
NALB	326.135	1.861	Negative	-1.088	-0.684	7.76E-04	0.01	0.583	Up	1/1,[Scopola	2/2,[C01416:	0	0	1/1,[Scopolamine]
NALB	256.175	2.964	Negative	-0.761	-0.613	7.76E-04	0.01	0.213	Up	0/0	0/0	0	0	0
NALB	236.113	14.002	Negative	-0.658	-0.527	7.76E-04	0.01	0.189	Up	0/0	0/0	1,{LMGL0301	0	1,{LMGL03012615: Triacetin < Triradylglycerols [GL03] < Glycerolipids [GL]}
NALB	123.097	0.713	Negative	-0.37	-0.003	7.76E-04	0.01	0.53	Up	0/0	0/0	0	0	0
NALB	212.176	1.436	Negative	0.095	0.589	7.76E-04	0.01	0.713	Up	0/0	0/0	0	0	0
NALB	243.098	2.331	Negative	-0.706	-0.089	1.78E-03	0.03	0.89	Up	1/1,[Thymidi	2/2,[C00214:	0	1,[THYMIDIN	1/1,[Thymidine]
NALB	144.982	10.526	Negative	0.565	1.584	1.78E-03	0.03	1.471	Up	0/0	0/0	0	0	0
NALB	130.533	6.08	Positive	0	5814197.78	1.78E-03	0.03	0	Up	0/0	0/0	0	0	0
NALB	130.533	6.784	Positive	0	167796701	1.78E-03	0.03	0	Up	0/0	0/0	0	0	0
NALB	158.129	0.768	Positive	0	107309747	1.78E-03	0.03	0	Up	0/0	0/0	0	0	0
NALB	178.134	3.086	Positive	0	17721961	1.78E-03	0.03	0	Up	2/2,[Tryptam	1/2,[C00398:	0	1,[TRYPTAM	2/2,[Tryptamine],[Anatabine]
NALB	243.158	2.496	Positive	0	2.3106E+10	1.78E-03	0.03	0	Up	0/0	0/0	0	0	0
NALB	267.268	9.607	Positive	0	6632265832	1.78E-03	0.03	0	Up	0/0	0/0	15,{LMFA050	0	15,{LMFA05000047: 3Z,13Z-octadecadien-1-ol < Fatty alcohols [FA05] < Fatty Acyls [FA]},{LMFA0500004[illegible]
NALB	129.074	0.787	Negative	0.243	0.596	3.21E-03	0.04	0.509	Up	0/0	0/0	0	0	0
NALB	219.19	2.028	Negative	-1.055	-0.672	4.50E-03	0.05	0.552	Up	0/0	0/0	0	0	0
NALB	102.092	11.958	Negative	-0.84	0.001	4.50E-03	0.05	1.213	Up	3/3,[5-Amin	4/5,[C12455:	4,{LMFA0801	1,[PRENAL: 3	3/3,[5-Aminopentanal],[Methyl propenyl ketone],[3-Methyl-2-butenal]
NALB	125.043	0.809	Negative	0.17	0.345	4.50E-03	0.05	0.252	Up	0/0	0/0	0	0	0
NALB	105.043	9.635	Negative	2.366	2.727	4.50E-03	0.05	0.521	Up	0/0	0/0	0	0	0
SWM	324.885	13.323	Negative	-1.353	-1.686	0	0	-0.481	Down	0/0	0/0	0	0	0
SWM	318.129	1.4	Positive	-1.35	-1.538	0	0	-0.27	Down	0/0	0/0	0	0	0
SWM	201.039	1.571	Negative	-1.262	-1.618	0	0	-0.513	Down	3/3,[Succinyl	4/4,[C00502:	0	0	3/3,[Succinylacetoacetate],[Arabinonic acid],[Ribonic acid]
SWM	277.939	13.255	Negative	-1.143	-1.4	0	0	-0.37	Down	0/0	0/0	0	0	0
SWM	681.483	10.126	Positive	-1.114	-1.739	0	0	-0.901	Down	0/0	0/0	8,{LMGP1002	0	8,{LMGP10020076: PA(O-16:0/20:5(5Z,8Z,11Z,14Z,17Z)) < Glycerophosphates [GP10] < Glycerophospholi[illegible]
SWM	686.439	10.159	Positive	-1.11	-1.605	0	0	-0.714	Down	0/0	0/0	1,{LMGP0303	0	1,{LMGP03030001: PS(P-16:0/12:0) < Glycerophosphoserines [GP03] < Glycerophospholipids [GP]}
SWM	249.144	1.463	Positive	-0.981	-1.22	0	0	-0.345	Down	2/2,[Isovaler	0/0	0	0	2/2,[Isovalerylglutamic acid],[Suberylglycine]
SWM	258.218	2.553	Positive	-0.956	-1.394	0	0	-0.632	Down	0/0	0/0	0	0	0
SWM	328.907	13.231	Negative	-0.943	-1.336	0	0	-0.567	Down	0/0	0/0	0	0	0
SWM	200.128	4.075	Positive	-0.935	-1.107	0	0	-0.248	Down	2/2,[Ecgonin	2/2,[C12448:	4,{LMFA0103	1,[CPD-3183:	2/2,[Ecgonine methyl ester],[N-Methylnicotinium]
SWM	180.159	14.002	Positive	-0.843	-0.922	0	0	-0.113	Down	0/0	0/0	0	0	0
SWM	447.208	1.609	Positive	-0.751	-1.693	0	0	-1.358	Down	0/0	0/0	0	0	0
SWM	256.014	1.646	Positive	-0.739	-0.888	0	0	-0.215	Down	0/0	0/0	0	0	0
SWM	202.11	1.366	Negative	-0.658	-1.386	0	0	-1.05	Down	1/1,[L-Acetyl	0/1	0	1,[CPD-4209:	1/1,[L-Acetylcarnitine]
SWM	131.162	4.16	Positive	-0.603	-0.766	0	0	-0.235	Down	0/0	0/0	0	0	0
SWM	245.161	1.576	Positive	-0.6	-1.494	0	0	-1.29	Down	0/0	0/0	0	0	0
SWM	330.177	0.782	Positive	-0.597	-1.019	0	0	-0.608	Down	0/0	0/0	0	0	0
SWM	349.87	13.481	Negative	-0.587	-0.784	0	0	-0.284	Down	0/0	0/0	0	0	0
SWM	618.527	9.601	Negative	-0.557	-1.156	0	0	-0.864	Down	0/0	0/0	0	0	0
SWM	153.031	0.907	Negative	-0.528	-0.93	0	0	-0.579	Down	0/0	1/1,[C03277:	0	0	1/1,[C03277: Imidazol-5-yl-pyruvate; 3-(Imidazol-5-yl)pyruvate; Imidazole pyruvate; Imidazole pyruvic ac[illegible]
SWM	154.929	0.573	Negative	-0.357	-0.998	0	0	-0.924	Down	0/0	0/0	0	0	0
SWM	157.134	0.917	Positive	-0.33	-1.014	0	0	-0.987	Down	0/0	1/2,[C00783:	0	0	1/2,[C00783: Tropinone; 3-Tropanone]
SWM	117.044	1.577	Negative	-0.31	-0.915	0	0	-0.873	Down	0/0	0/0	0	0	0
SWM	192.062	13.288	Positive	-0.298	-0.496	0	0	-0.286	Down	0/0	0/0	0	0	0
SWM	331.975	13.126	Negative	-0.297	-0.466	0	0	-0.245	Down	0/0	0/0	0	0	0
SWM	262.119	0.86	Positive	-0.256	-0.463	0	0	-0.298	Down	1/1,[Indolyla	0/0	0	0	1/1,[Indolylacryloylglycine]
SWM	317.149	5.486	Positive	-0.245	-0.54	0	0	-0.425	Down	0/0	0/0	0	0	0
SWM	136.91	13.443	Negative	-0.155	-0.284	0	0	-0.187	Down	0/0	0/0	0	0	0
SWM	130.537	13.231	Positive	-0.096	-0.295	0	0	-0.286	Down	0/0	0/0	0	0	0
SWM	107.071	13.962	Positive	-0.085	-0.644	0	0	-0.807	Down	0/0	1/1,[C06178:	0	0	1/1,[C06178: 1-Methylpyrrolinium; 1-Methyl-delta(1)-pyrrolinium]
SWM	746.562	8.838	Positive	0.01	-0.54	0	0	-0.793	Down	0/0	0/0	0	0	0
SWM	328.223	3.463	Positive	0.031	-0.776	0	0	-1.164	Down	0/0	0/0	0	0	0
SWM	189.933	13.276	Negative	0.072	-0.039	0	0	-0.161	Down	0/0	0/0	0	0	0
SWM	132.537	13.253	Positive	0.072	-0.088	0	0	-0.232	Down	0/0	0/0	0	0	0
SWM	477.9	13.677	Positive	0.089	-0.292	0	0	-0.549	Down	0/0	0/0	0	0	0
SWM	206.047	2.722	Negative	0.13	-0.102	0	0	-0.335	Down	1/1,[4-(2-Am	3/4,[C01252:	0	1,[CPD-476:	1/1,[4-(2-Aminophenyl)-2,4-dioxobutanoic acid]
SWM	617.524	10.729	Negative	0.146	-0.317	0	0	-0.668	Down	0/0	0/0	0	0	0
SWM	395.367	9.796	Negative	0.157	-0.254	0	0	-0.593	Down	0/0	0/0	1,{LMST0101	0	1,{LMST01010085: 5beta-cholestane < Sterols [ST01] < Sterol Lipids [ST]}
SWM	702.536	8.851	Positive	0.16	-0.31	0	0	-0.678	Down	0/0	0/0	0	0	0
SWM	128.536	13.329	Positive	0.202	-0.097	0	0	-0.431	Down	0/0	0/0	0	0	0
SWM	139.135	0.53	Positive	0.216	-0.029	0	0	-0.354	Down	0/0	0/0	0	0	0
SWM	232.085	1.078	Negative	0.225	-0.723	0	0	-1.368	Down	0/0	0/0	0	0	0
SWM	147.067	3.088	Negative	0.286	-0.176	0	0	-0.666	Down	2/2,[Mevalo	3/3,[C00418:	1,{LMFA0105	1,[MEVALON	2/2,[Mevalonic acid],[(R) 2,3-Dihydroxy-3-methylvalerate]
SWM	347	13.099	Negative	0.306	0.052	0	0	-0.367	Down	0/0	0/0	0	0	0
SWM	76.04	13.745	Positive	0.313	-1.041	0	0	-1.953	Down	0/0	0/0	0	0	0
SWM	320.889	13.46	Negative	0.329	0.184	0	0	-0.21	Down	0/0	0/0	0	0	0
SWM	137.025	4.613	Negative	0.361	0.095	0	0	-0.384	Down	8/10,[4-Hydr	7/10,[C0015(	6,{LMFA0105	5,[4-hydroxyl	8/10,[4-Hydroxybenzoic acid],[Gentisate aldehyde],[2-Ketobutyric acid],[Acetoacetic acid],[2-Methyl-3-o[illegible]
SWM	164.093	1.637	Positive	0.419	-0.002	0	0	-0.608	Down	1/1,[6-Dimet	0/0	0	0	1/1,[6-Dimethylaminopurine]
SWM	552.341	13.174	Negative	0.454	-1.196	0	0	-2.38	Down	0/0	0/0	0	0	0
SWM	117.056	5.075	Negative	0.548	-1.242	0	0	-2.582	Down	11/11,[3-Hyc	0/0	13,{LMFA010	0	11/11,[3-Hydroxy-2-methyl-[R-(R,S)]-butanoic acid],[2-Methyl-3-hydroxybutyric acid],[2-Ethylhydracrylic [illegible]
SWM	453.266	8.479	Negative	0.564	-0.509	0	0	-1.547	Down	1/1,[Simvast	0/0	1,{LMST0116	0	1/1,[Simvastatin]
SWM	180.974	13.153	Negative	0.573	0.466	0	0	-0.155	Down	0/0	0/0	0	0	0
SWM	190.107	0.797	Positive	0.651	0.148	0	0	-0.726	Down	3/3,[2-Octen	0/0	3,{LMFA0106	0	3/3,[2-Octenedioic acid],[cis-4-Octenedioic acid],[trans-3-Octenedioic acid]
SWM	482.307	13.233	Negative	0.667	-0.323	0	0	-1.428	Down	0/0	0/0	0	0	0
SWM	161.046	1.568	Negative	0.669	0.274	0	0	-0.57	Down	6/7,[2-Hydro	10/10,[C010(	4,{LMFA0117	0	6/7,[2-Hydroxyadipic acid],[3-Hydroxyadipic acid],[3-Hydroxymethylglutaric acid],[2(R)-Hydroxyadipic aci[illegible]
SWM	117.056	0.835	Negative	0.811	0.036	0	0	-1.118	Down	11/11,[3-Hyc	0/0	13,{LMFA010	0	11/11,[3-Hydroxy-2-methyl-[R-(R,S)]-butanoic acid],[2-Methyl-3-hydroxybutyric acid],[2-Ethylhydracrylic [illegible]
SWM	103.051	0.483	Positive	0.83	0.663	0	0	-0.241	Down	0/0	1/1,[C02718:	1,{LMPK1400	0	1/1,[C02718: N-Formiminoglycine; Formiminoglycine; N-Formimidoylglycine]
SWM	267.053	2.635	Negative	0.844	-0.326	0	0	-1.689	Down	0/0	0/0	0	0	0
SWM	133.045	1.541	Negative	0.889	0.395	0	0	-0.713	Down	0/0	0/0	0	0	0
SWM	144.982	13.605	Positive	1.075	0.975	0	0	-0.144	Down	0/0	0/0	0	0	0
SWM	235.928	6.55	Negative	1.075	0.698	0	0	-0.545	Down	0/0	0/0	0	0	0
SWM	450.262	6.509	Negative	1.125	0.627	0	0	-0.719	Down	2/2,[LysoPE((	0/0	2,{LMGP0102	0	2/2,[LysoPE(0:0/16:1(9Z))],[LysoPE(16:1(9Z)/0:0)]
SWM	138.091	1.031	Positive	1.283	0.775	0	0	-0.732	Down	8/8,[Tyramir	7/9,[C00483:	0	3,[NN-DIMET	8/8,[Tyramine],[2-Hydroxyphenethylamine],[Dimethylaniline-N-oxide],[m-Tyramine],[4-Hydroxystyrene],[illegible]
SWM	106.046	12.134	Positive	1.338	1.038	0	0	-0.434	Down	0/0	0/0	0	0	0
SWM	481.303	11.202	Negative	1.517	0.238	0	0	-1.845	Down	0/0	0/0	0	0	0
SWM	118.123	13.098	Positive	2.61	2.48	0	0	-0.188	Down	6/6,[3-Hexar	1/2,[C02373:	12,{LMFA050	3,[4-METHYL	6/6,[3-Hexanone],[4-Methylpentanal],[Methyl isobutyl ketone],[2-Oxohexane],[Ethyl isopropyl ketone],[H[illegible]
SWM	72.081	13.806	Positive	180417.846	0	1.78E-03	0.03	44326312.4	Down	0/0	0/0	0	0	0
SWM	228.05	1.742	Positive	197541.913	0	1.78E-03	0.03	4125709.65	Down	2/2,[5-(2'-Ca	3/3,[C05655:	1,{LMFA0801	1,[LIPOAMID	2/2,[5-(2'-Carboxyethyl)-4,6-Dihydroxypicolinate],[Lipoamide]
SWM	114.055	13.603	Positive	208179.923	0	1.78E-03	0.03	958244.359	Down	2/2,[1-Pyrrol	2/4,[C03564:	0	1,[L-DELTA1-	2/2,[1-Pyrroline-5-carboxylic acid],[1-Pyrroline-2-carboxylic acid]
SWM	352.857	0.307	Negative	237298.98	0	1.78E-03	0.03	1253665.58	Down	0/0	0/0	0	0	0
SWM	220.056	0.888	Negative	245297.848	0	1.78E-03	0.03	2933853.8	Down	0/0	0/0	0	0	0
SWM	517.224	4.543	Negative	298385.023	0	1.78E-03	0.03	1723902.28	Down	0/0	0/0	0	0	0
SWM	167.024	1.129	Negative	331191.528	0	1.78E-03	0.03	2585925.45	Down	0/0	0/0	0	0	0
SWM	323.078	4.754	Negative	358462.204	0	1.78E-03	0.03	8581314.09	Down	0/0	0/0	0	0	0
SWM	185.083	4.254	Negative	385808.622	0	1.78E-03	0.03	2520362.75	Down	0/0	0/0	0	0	0
SWM	446.254	7.028	Positive	392030.907	0	1.78E-03	0.03	872908.912	Down	0/0	0/0	0	0	0
SWM	170.083	3.519	Negative	395219.219	0	1.78E-03	0.03	5766647.69	Down	0/0	0/0	1,{LMFA0803	0	1,{LMFA08030002: N-butanoyl-lhomoserine lactone < Fatty amides [FA08] < Fatty Acyls [FA]}
SWM	95.05	0.805	Negative	449212.162	0	1.78E-03	0.03	6023521.18	Down	0/0	2/2,[C01845:	3,{LMFA0600	0	2/2,[C01845: Propan-2-ol; 2-Propanol; Isopropanol; Isopropyl alcohol],[C05979: Propane-1-ol; Propanol; [illegible]
SWM	185.01	1.335	Negative	516164.464	0	1.78E-03	0.03	3095532.74	Down	0/0	4/8,[C01163:	1,{LMFA0117	0	4/8,[C01163: 3-Carboxy-cis,cis-muconate; beta-Carboxy-cis,cis-muconate; cis,cis-Butadiene-1,2,4-tricarb[illegible]
SWM	162.055	1.79	Positive	517665.852	0	1.78E-03	0.03	8759063.68	Down	2/3,[2-Indole	2/3,[C05637:	0	0	2/3,[2-Indolecarboxylic acid],[4,6-Dihydroxyquinoline]
SWM	449.111	0.629	Negative	530208.136	0	1.78E-03	0.03	3767612.62	Down	0/0	1/3,[C16999:	21,{LMPK120	0	1/3,[C16999: Futalosine]
SWM	94.038	1.126	Negative	541850.066	0	1.78E-03	0.03	3738788.63	Down	0/0	0/0	0	0	0
SWM	212.03	0.805	Negative	641772.167	0	1.78E-03	0.03	7905888.61	Down	0/0	0/0	0	0	0
SWM	274.059	0.853	Negative	831403.955	0	1.78E-03	0.03	6008845.68	Down	0/0	0/0	0	0	0
SWM	190.052	3.336	Negative	858303.961	0	1.78E-03	0.03	5740942.52	Down	1/1,[5-Hydro	2/2,[C05635:	0	1,[INDOLE-3-	1/1,[5-Hydroxyindoleacetic acid]
SWM	280.674	2.702	Positive	1135824.56	0	1.78E-03	0.03	7363539.82	Down	0/0	0/0	0	0	0
SWM	177.066	3.182	Negative	1246197.22	0	1.78E-03	0.03	5782989.76	Down	3/3,[4-Oxo-4	1/1,[C06231:	0	1,[CPD-3190:	3/3,[4-Oxo-4-(3-pyridyl)-butanamide],[BOX B],[BOX A]
SWM	218.069	0.714	Negative	1263383.54	0	1.78E-03	0.03	15484929.5	Down	1/1,[2,6-Diar	0/0	0	0	1/1,[2,6-Diamino-4-hydroxy-5-N-methylformamidopyrimidine]
SWM	101.079	13.983	Positive	1506104.87	0	1.78E-03	0.03	587275.482	Down	0/0	0/0	0	0	0
SWM	234.043	2.084	Negative	1819157.67	0	1.78E-03	0.03	21548541.5	Down	0/0	0/0	0	0	0
SWM	345.242	5.798	Positive	2209342.72	0	1.78E-03	0.03	23312840.6	Down	4/5,[17-HDol	0/0	33,{LMFA030	0	4/5,[17-HDoHE],[19(20)-EpDPE],[16(17)-EpDPE],[15(S)-Hydroxyeicosatrienoic acid]
SWM	203.184	4.349	Positive	2746185.18	0	1.78E-03	0.03	24454060.6	Down	0/0	0/0	0	0	0
SWM	156.066	1.125	Positive	3310826.17	0	1.78E-03	0.03	8651714.77	Down	2/4,[4-Hydro	3/6,[C00156:	0	1,[4-hydroxyl	2/4,[4-Hydroxybenzoic acid],[Gentisate aldehyde]
SWM	145.049	4.431	Negative	5347213.16	0	1.78E-03	0.03	4491798.26	Down	0/0	0/0	0	0	0
SWM	347.262	9.375	Negative	6556272.07	0	1.78E-03	0.03	1645518.55	Down	0/0	0/0	0	0	0
SWM	221.121	2.575	Positive	6796712.52	0	1.78E-03	0.03	20348555.3	Down	0/0	0/0	0	0	0
SWM	162.076	0.658	Positive	20091517.8	0	1.78E-03	0.03	162586432	Down	5/5,[Aminoa	6/8,[C00956:	5,{LMFA0117	1,[CPD-468: 2	5/5,[Aminoadipic acid],[3-Hexenedioic acid],[3-Methylglutaconic acid],[(E)-2-Methylglutaconic acid],[tra[illegible]
SWM	288.192	3.395	Positive	27061070.4	0	1.78E-03	0.03	29627531.3	Down	0/0	0/0	1,{LMSP0108	0	1,{LMSP01080050: Micropine < Sphingoid bases [SP01] < Sphingolipids [SP]}
SWM	379.183	10.092	Positive	-2.142	-1.962	0	0	0.26	Up	0/0	0/0	0	0	0
SWM	596.487	10.644	Negative	-2.029	-0.507	0	0	2.196	Up	0/0	0/0	0	0	0
SWM	310.005	1.943	Negative	-1.57	-1.046	0	0	0.755	Up	0/0	0/0	0	0	0
SWM	417.245	2.113	Positive	-1.538	-1.091	0	0	0.645	Up	0/0	0/0	0	0	0
SWM	592.267	5.271	Negative	-1.346	-0.733	0	0	0.885	Up	0/0	0/0	0	0	0
SWM	477.308	13.127	Negative	-1.303	0.335	0	0	2.364	Up	0/0	0/0	0	0	0
SWM	306.112	1.831	Positive	-1.298	-0.421	0	0	1.265	Up	2/2,[N-Phen	0/1	0	0	2/2,[N-Phenylacetylphenylalanine],[Morphinone]
SWM	623.412	8.911	Negative	-1.229	-0.745	0	0	0.698	Up	0/0	0/0	1,{LMPR0107	0	1,{LMPR01070154: Diketospirilloxanthin/ 2,2'-Diketospirilloxanthin < Isoprenoids [PR01] < Prenol Lipids [[illegible]
SWM	546.452	10.424	Positive	-1.068	-0.579	0	0	0.705	Up	0/0	0/0	0	0	0
SWM	297.121	4.869	Negative	-1.062	1.614	0	0	3.862	Up	0/0	0/0	0	0	0
SWM	404.036	0.688	Positive	-0.927	-0.07	0	0	1.236	Up	0/0	0/0	0	0	0
SWM	356.145	1.851	Positive	-0.917	-0.685	0	0	0.336	Up	0/0	0/0	0	0	0
SWM	419.215	4.542	Negative	-0.852	-0.25	0	0	0.869	Up	0/0	0/0	0	0	0
SWM	282.133	13.331	Negative	-0.735	-0.157	0	0	0.835	Up	0/0	0/0	0	0	0
SWM	413.323	9.78	Positive	-0.651	-0.201	0	0	0.65	Up	0/0	0/0	0	0	0
SWM	245.095	4.223	Negative	-0.641	0.133	0	0	1.117	Up	0/0	0/0	0	0	0
SWM	356.352	5.377	Positive	-0.636	0.126	0	0	1.099	Up	2/2,[Erucic a	1/1,[C08316:	18,{LMFA080	0	2/2,[Erucic acid],[Cetoleic acid]
SWM	281.135	7.223	Negative	-0.634	0.486	0	0	1.616	Up	0/0	0/0	0	0	0
SWM	486.363	11.034	Positive	-0.632	-0.023	0	0	0.878	Up	0/0	0/0	0	0	0
SWM	172.118	2.626	Negative	-0.623	-0.194	0	0	0.62	Up	0/0	0/0	0	0	0
SWM	289.132	6.514	Negative	-0.618	1.676	0	0	3.309	Up	1/1,[Homoar	0/0	0	0	1/1,[Homoanserine]
SWM	73.029	1.02	Negative	-0.587	-0.15	0	0	0.631	Up	2/5,[D-Lacta	6/6,[C00163:	1,{LMFA0101	3,[ACETOL: a	2/5,[D-Lactaldehyde],[Hydroxyacetone]
SWM	413.218	3.014	Positive	-0.533	0.04	0	0	0.828	Up	0/0	0/0	0	0	0
SWM	358.368	5.271	Positive	-0.49	0.031	0	0	0.751	Up	1/1,[Behenic	1/1,[C08281:	13,{LMFA010	0	1/1,[Behenic acid]
SWM	207.087	4.061	Negative	-0.441	0.322	0	0	1.101	Up	0/0	0/0	0	0	0
SWM	168.123	1.342	Positive	-0.378	0.803	0	0	1.704	Up	0/0	0/0	0	0	0
SWM	534.25	5.544	Negative	-0.374	0.159	0	0	0.769	Up	0/0	0/0	0	0	0
SWM	455.215	5.521	Negative	-0.232	0.26	0	0	0.709	Up	0/0	0/0	0	0	0
SWM	168.025	0.8	Negative	-0.18	0.108	0	0	0.415	Up	0/0	0/0	0	0	0
SWM	263.062	2.844	Negative	-0.044	0.591	0	0	0.916	Up	0/0	0/0	0	0	0
SWM	296.62	13.541	Negative	-0.033	1.103	0	0	1.639	Up	0/0	0/0	0	0	0
SWM	115.921	11.195	Negative	-0.024	2.349	0	0	3.423	Up	0/0	0/0	0	0	0
SWM	157.099	1.07	Negative	0.032	0.7	0	0	0.963	Up	0/0	0/0	0	0	0
SWM	485.36	11.038	Positive	0.058	0.654	0	0	0.86	Up	0/0	0/0	10,{LMST030	0	10,{LMST03020485: (22R)-1alpha,25-dihydroxy-22-methoxy-26,27-dimethyl-23,23,24,24-tetradehydro-24[illegible]
SWM	123.081	11.945	Positive	0.107	1.118	0	0	1.459	Up	0/0	5/6,[C05853:	1,{LMFA0600	1,[CPD-7035:	5/6,[C05853: Phenylethyl alcohol; Phenethyl alcohol; 2-Phenylethanol],[C06757: 4-Methylbenzyl alcohol; [illegible]
SWM	415.183	4.291	Negative	0.197	0.614	0	0	0.602	Up	0/0	0/0	0	0	0
SWM	289.132	7.57	Negative	0.225	2.316	0	0	3.017	Up	1/1,[Homoar	0/0	0	0	1/1,[Homoanserine]
SWM	201.147	2.318	Positive	0.235	0.547	0	0	0.45	Up	0/0	0/0	0	0	0
SWM	208.097	4.076	Positive	0.262	0.763	0	0	0.724	Up	3/3,[N-Acety	2/3,[C03519:	0	0	3/3,[N-Acetyl-L-phenylalanine],[Phenylpropionylglycine],[3-Phenylpropionylglycine]
SWM	297.121	6.086	Negative	0.288	0.792	0	0	0.727	Up	0/0	0/0	0	0	0
SWM	125.986	0.504	Positive	0.345	0.906	0	0	0.808	Up	0/0	0/0	0	0	0
SWM	256.84	0.692	Negative	0.393	0.628	0	0	0.338	Up	0/0	0/0	0	0	0
SWM	186.16	1.107	Positive	0.704	1.967	0	0	1.822	Up	0/0	0/0	0	0	0
SWM	132.868	10.124	Negative	0.82	1.454	0	0	0.915	Up	0/0	0/0	0	0	0
SWM	109.522	13.543	Positive	0.979	1.115	0	0	0.197	Up	0/0	0/0	0	0	0
SWM	126.905	12.053	Negative	0	5024741.1	1.78E-03	0.03	0	Up	0/0	0/0	0	0	0
SWM	170.022	12.051	Negative	0	203441569	1.78E-03	0.03	0	Up	0/0	0/0	0	0	0
SWM	170.022	11.193	Negative	0	244450534	1.78E-03	0.03	0	Up	0/0	0/0	0	0	0
SWM	189.933	13.522	Negative	0	5819313.82	1.78E-03	0.03	0	Up	0/0	0/0	0	0	0
SWM	253.166	1.643	Positive	0	1722933.72	1.78E-03	0.03	0	Up	0/0	0/0	0	0	0
SWM	279.617	13.523	Negative	0	19965115.3	1.78E-03	0.03	0	Up	0/0	0/0	0	0	0
SWM	290.13	13.443	Negative	0	17725890.4	1.78E-03	0.03	0	Up	0/0	0/0	0	0	0
SWM	296.62	5.595	Negative	0	114296798	1.78E-03	0.03	0	Up	0/0	0/0	0	0	0
SWM	351.188	6.322	Negative	0	8536301.88	1.78E-03	0.03	0	Up	0/0	0/0	1,{LMFA0109	0	1,{LMFA01090059: 9,10-dichloro-octadecanoic acid < Fatty Acids and Conjugates [FA01] < Fatty Acyls [F[illegible]
SWM	362.098	13.487	Negative	0	3488101.25	1.78E-03	0.03	0	Up	0/0	0/0	0	0	0
SWM	365.203	6.665	Negative	0	2701421.58	1.78E-03	0.03	0	Up	0/0	0/0	0	0	0
SWM	371.358	12.146	Positive	0	2779941.1	1.78E-03	0.03	0	Up	0/0	0/0	0	0	0
SWM	416.237	4.123	Positive	0	2876232.24	1.78E-03	0.03	0	Up	0/0	0/0	0	0	0
SWM	483.264	7.008	Negative	0	2792034.38	1.78E-03	0.03	0	Up	0/0	0/0	0	0	0
SWM	490.425	12.847	Positive	0	2896562.19	1.78E-03	0.03	0	Up	0/0	1/1,[C13202:	5,{LMST0104	0	1/1,[C13202: alpha-Tocopherol acetate; Tocopherol acetate]
SWM	544.509	10.722	Positive	0	1676487.53	1.78E-03	0.03	0	Up	0/0	0/0	5,{LMFA0103	0	5,{LMFA01030836: 36:5(21Z,24Z,27Z,30Z,33Z) < Fatty Acids and Conjugates [FA01] < Fatty Acyls [FA]},{L[illegible]
SWM	590.475	12.137	Positive	0	1184556.92	1.78E-03	0.03	0	Up	0/0	0/0	2,{LMPR0400	0	2,{LMPR04000014: bacteriohopane-32,33, 34-triol-35-carbamate < Hopanoids [PR04] < Prenol Lipids [PR[illegible]
SWM	661.538	12.045	Positive	0	4524165.41	1.78E-03	0.03	0	Up	3/3,[TG(12:0	0/0	2,{LMGL0301	0	3/3,[TG(12:0/12:0/12:0)],[Glucosylceramide (d18:1/12:0)],[Galactosylceramide (d18:1/12:0)]
SWM	94.547	13.292	Positive	0	6322295.25	1.78E-03	0.03	0	Up	0/0	0/0	0	0	0

bioRxiv preprint doi: https://doi.org/10.1101/2024.10.28.620613; this version posted October 28, 2024. The copyright holder for this preprint (which was not certified by peer review) is the author/funder, who has granted bioRxiv a license to display the preprint in perpetuity. It is made available under aCC-BY 4.0 International license.

Table S4. The significant positive and negative correlations between microbial metabolites and host hepatic gene expression for those metabolites/genes significantly altered by 1.5% oxalate consumpti[illegible]

Microbiome	Metabolite ID	Mouse Gene	Pearson Correlation	FDR
NALB	Unknown	Amine sulfotransferase (EC 2.8.2.3) (SULT-X2) (Sulfotransferase 3A1) (ST3A1)	-0.886	0
NALB	Unknown	Amine sulfotransferase (EC 2.8.2.3) (SULT-X2) (Sulfotransferase 3A1) (ST3A1)	-0.867	0
NALB	Unknown	unknown	-0.759	0
NALB	Unknown	Amine sulfotransferase (EC 2.8.2.3) (SULT-X2) (Sulfotransferase 3A1) (ST3A1)	-0.782	0.01
NALB	Unknown	unknown	-0.737	0
NALB	Unknown	unknown	-0.735	0
NALB	Unknown	unknown	-0.73	0
NALB	Unknown	unknown	-0.718	0
NALB	Unknown	unknown	-0.696	0.02
NALB	Unknown	Amine sulfotransferase (EC 2.8.2.3) (SULT-X2) (Sulfotransferase 3A1) (ST3A1)	-0.691	0.05
NALB	Unknown	60S ribosomal protein L26	-0.69	0
NALB	Unknown	unknown	-0.673	0.01
NALB	Unknown	Amine sulfotransferase (EC 2.8.2.3) (SULT-X2) (Sulfotransferase 3A1) (ST3A1)	-0.669	0.05
NALB	Unknown	F-actin-uncapping protein LRRC16A	-0.668	0
NALB	Unknown	Uncharacterized protein	-0.656	0.01
NALB	Unknown	F-actin-uncapping protein LRRC16A	-0.655	0
NALB	Unknown	La ribonucleoprotein domain family, member 1B	-0.647	0.02
NALB	Unknown	60S ribosomal protein L26	-0.643	0.01
NALB	Unknown	unknown	-0.636	0.06
NALB	Unknown	60S ribosomal protein L26	-0.631	0.08
NALB	Unknown	60S ribosomal protein L26	-0.63	0.06
NALB	Unknown	Calcium-responsive transcription factor (ECBRC-FC1)	-0.624	0.05
NALB	Unknown	unknown	-0.624	0.02
NALB	Unknown	F-actin-uncapping protein LRRC16A	-0.623	0
NALB	Unknown	F-actin-uncapping protein LRRC16A	-0.609	0
NALB	Unknown	unknown	-0.602	0
NALB	Unknown	Calcium-responsive transcription factor (ECBRC-FC1)	-0.597	0.06
NALB	Unknown	unknown	-0.589	0.06
NALB	Unknown	unknown	-0.579	0.08
NALB	Unknown	unknown	-0.571	0.05
NALB	Unknown	Uncharacterized protein	-0.569	0
NALB	Unknown	Cholesterol 24-hydroxylase (Fragment)	-0.561	0
NALB	Unknown	unknown	-0.561	0
NALB	Unknown	unknown	-0.557	0.08
NALB	Unknown	unknown	-0.55	0.06
NALB	Unknown	unknown	-0.534	0.02
NALB	Unknown	unknown	-0.533	0.05
NALB	Unknown	unknown	-0.529	0
NALB	Unknown	unknown	-0.529	0.01
NALB	Unknown	unknown	-0.525	0.05
NALB	Unknown	Uncharacterized protein	-0.522	0
NALB	Unknown	unknown	-0.517	0.06
NALB	Unknown	Uncharacterized protein	-0.506	0.01
NALB	Unknown	60S ribosomal protein L26	-0.503	0.08
NALB	Unknown	Cholesterol 24-hydroxylase (Fragment)	-0.503	0.02
NALB	Unknown	unknown	-0.502	0.06
NALB	Unknown	AMP deaminase 3 (EC 3.5.4.6) (AMP deaminase H-type) (AMP deaminase isoform E) (Heart-type AMPD)	-0.496	0.06
NALB	Unknown	unknown	-0.496	0.05
NALB	Unknown	unknown	-0.494	0.01
NALB	Unknown	unknown	-0.494	0.02
NALB	Unknown	Cholesterol 24-hydroxylase (Fragment)	-0.494	0
NALB	Unknown	unknown	-0.492	0.08
NALB	Unknown	Uncharacterized protein	-0.491	0.01
NALB	Unknown	Uncharacterized protein	-0.487	0.08
NALB	Unknown	La ribonucleoprotein domain family, member 1B	-0.485	0.05
NALB	Unknown	unknown	-0.485	0.02
NALB	Unknown	unknown	-0.483	0.05
NALB	Unknown	Cholesterol 24-hydroxylase (Fragment)	-0.48	0.02
NALB	Unknown	unknown	-0.48	0.06
NALB	Unknown	unknown	-0.477	0.08
NALB	Unknown	Uncharacterized protein	-0.474	0.01
NALB	Unknown	60S ribosomal protein L26	-0.472	0.05
NALB	Unknown	unknown	-0.471	0
NALB	Unknown	unknown	-0.468	0.05
NALB	Unknown	unknown	-0.461	0.08
NALB	Unknown	Uncharacterized protein	-0.456	0.05
NALB	Unknown	Uncharacterized protein	-0.454	0.01
NALB	Unknown	unknown	-0.45	0
NALB	Unknown	unknown	-0.449	0.05
NALB	Unknown	unknown	-0.448	0.05
NALB	Unknown	Cholesterol 24-hydroxylase (Fragment)	-0.448	0.06
NALB	Unknown	Uncharacterized protein	-0.446	0.01
NALB	Unknown	unknown	-0.446	0.08
NALB	Unknown	Uncharacterized protein	-0.445	0.02
NALB	Unknown	unknown	-0.445	0
NALB	Unknown	unknown	-0.444	0.05
NALB	Unknown	Uncharacterized protein	-0.436	0.08
NALB	Unknown	Uncharacterized protein	-0.434	0.05
NALB	Unknown	Cholesterol 24-hydroxylase (Fragment)	-0.431	0.02
NALB	Unknown	unknown	-0.428	0.06
NALB	Unknown	unknown	-0.424	0.05
NALB	Unknown	Uncharacterized protein	-0.405	0.05
NALB	Unknown	Uncharacterized protein	-0.39	0.05
NALB	Unknown	unknown	0.329	0.05
NALB	Unknown	unknown	0.329	0.05
NALB	Unknown	unknown	0.352	0.06
NALB	Unknown	Cholesterol 24-hydroxylase (Fragment)	0.354	0.05
NALB	Unknown	unknown	0.36	0.05
NALB	Unknown	unknown	0.367	0.06
NALB	Unknown	unknown	0.388	0.08
NALB	Unknown	Cholesterol 24-hydroxylase (Fragment)	0.413	0.02
NALB	Unknown	unknown	0.424	0.06
NALB	Unknown	unknown	0.434	0
NALB	Unknown	unknown	0.436	0.02
NALB	Unknown	unknown	0.44	0
NALB	Unknown	unknown	0.461	0.06
NALB	Unknown	unknown	0.462	0.01
NALB	Unknown	unknown	0.463	0.06
NALB	Unknown	unknown	0.464	0.02
NALB	Unknown	unknown	0.469	0.01
NALB	Unknown	Cholesterol 24-hydroxylase (Fragment)	0.474	0.08
NALB	Unknown	unknown	0.479	0.06
NALB	Unknown	unknown	0.514	0.06
[illegible]	[illegible]	[illegible]	[illegible]	[illegible]

1613

bioRxiv preprint doi: https://doi.org/10.1101/2024.10.28.620613; this version posted October 28, 2024. The copyright holder for this preprint (which was not certified by peer review) is the author/funder, who has granted bioRxiv a license to display the preprint in perpetuity. It is made available under aCC-BY 4.0 International license.

1614

Table S5. The number of microbial genes in each metabolic pathway that exhibited a significant increase or decrease in abundance upon consumption of 6% oxalate by the host.

OTU	Test-Statistic	P	FDR	6% oxalate a	0% oxalate averag	Gene	Direction	Kegg pathways
[illegible]_179040	9.706	6.86E-05	7.93E-03	7.272	3.647	ATP phosphoribosyltransferase	Up	histidine metabolism
[illegible]_91376	8.146	1.88E-04	7.93E-03	6.201	2.317	ATP phosphoribosyltransferase regulat	Up	histidine metabolism
[illegible]_88298	7.774	2.38E-04	7.93E-03	6.402	2.647	UDP-3-O-acylglucosamine N-acyltransc	Up	lipopolysaccharide metabolism
[illegible]_170696	8.279	1.68E-04	7.93E-03	6.372	2.773	Ribulose-phosphate 3-epimerase	Up	pentose phosphate pathway; pentose and glucuronate
[illegible]_79182	9.633	7.17E-05	7.93E-03	6.408	2.982	Thiamine-monophosphate kinase	Up	thiamine metabolism
[illegible]_245042	7.869	2.26E-04	7.93E-03	6.564	3.671	Acetolactate synthase isozyme 3 large	Up	Valine, leucine, and isoleucine biosynthesis
[illegible]_42186	11.772	2.27E-05	7.93E-03	5.868	1.529	Lipopolysaccharide export system prot	Up	
[illegible]_98397	11.084	3.21E-05	7.93E-03	6.407	2.281	hypothetical protein	Up	
[illegible]_121225	10.993	3.37E-05	7.93E-03	6.291	2.697	hypothetical protein	Up	
[illegible]_204892	10.601	4.15E-05	7.93E-03	5.917	0.786	hypothetical protein	Up	
[illegible]	[illegible]	[illegible]	[illegible]	[illegible]	[illegible]	[illegible]	[illegible]	[illegible]

bioRxiv preprint doi: https://doi.org/10.1101/2024.10.28.620613; this version posted October 28, 2024. The copyright holder for this preprint (which was not certified by peer review) is the author/funder, who has granted bioRxiv a license to display the preprint in perpetuity. It is made available under aCC-BY 4.0 International license.

Table S7. Minimal media used to quantify the proportion of the microbiota that can use sole carb

**Component**	**Amount/L**	**Concentration**	
KH_2PO4	100 mL	100 mM	
$NaHCO_3$	0.4 g	4.8 mM	
$NaCl_2$	0.08 g	1.37 mM	
CaCl2	1 mL	0.80%	0.8g/100mL stock; autoclave
Vitamin K (menadione)	1 mL	5.8 mM	1 mg/mL stock solution; protect from light; autoclave
$FeSO_4$	1 mL	1.44 mM	0.4 mg $FeSO_4$/mL stock solution; filter sterilize
Histidine Hematin Solution	1 mL	0.1%	1.2 mg hematin/mL in 0.2M histidine; filter sterilize after preparation of stock; protect from light; store at 4C
NH4Cl	1 g		
Tween 80	2 mL	0.05%	
$MgSO_4$-$7H_20$***	1 mL	0.008 mM	
ATCC Vitamin Mix***	10 mL	1%	
ATCC Trace Mineral Mix***	10 mL	1%	
For sole C&E media, add one of the following to Media A			
Aspartic acid	0.02g		
Mannose	0.9g		
Galactose	1.8g		
Sodium acetate	6g	0.60%	
Sodium formate	4g	0.40%	Requires sodium sulfate
D-glucose	0.4 g	2.2 mM	Add to 10ml DI H2O and filter sterilize
Fructose	1 g	2.2 mM	Add to 10ml DI H2O and filter sterilize
Sucrose	1.71g		Add to 10ml DI H2O and filter sterilize
Methylvaleric acid	561ul		Add both methylvaeric and methylbutyric
Methylbutyric acid	517ul		
Oxalate	6.75g	50mM	
pH to 7			
Filter sterilize and bring into anaerobic chamber			
Cysteine***	0.5g		Add to 10ml DI H2O and filter sterilize in anaerobic chamber
sodium sulfate	2g	0.20%	Only add if adding sodium formate
**Let media sit in anaerobic chamber for at least 24hrs to be fully reduced before inoculation			

1615

bioRxiv preprint doi: https://doi.org/10.1101/2024.10.28.620613; this version posted October 28, 2024. The copyright holder for this preprint (which was not certified by peer review) is the author/funder, who has granted bioRxiv a license to display the preprint in perpetuity. It is made available under aCC-BY 4.0 International license.

Table S8. Microorganisms used in the taxonomic cohort.

**Species**	**Commercial source**	**Isolation source**
Oxalobacter formgienes	ATCC 35274	human
Desulfovibrio piger	DSMZ749	Sheep rumen
Methanobrevibacter smithii	DSMZ11975	animal feces
Ruminococcus gnavus	ATCC 29149	human
Coprococcus comes	ATCC 27758	human
Bacteroides thetaiotaomicron	ATCC 700349	human
Lactobacillus helveticus	ATCC 55796	Unknown
Lactobacillus plantarum	ATCC 700210	Unknown

bioRxiv preprint doi: https://doi.org/10.1101/2024.10.28.620613; this version posted October 28, 2024. The copyright holder for this preprint (which was not certified by peer review) is the author/funder, who has granted bioRxiv a license to display the preprint in perpetuity. It is made available under aCC-BY 4.0 International license.

Supplemental references

1. Kohl, K.D., and Dearing, M.D. (2014). Wild-caught rodents retain a majority of their natural gut microbiota upon entrance into captivity. Environmental microbiology reports *6*, 191-195.
2. Miller, A.W., Oakeson, K.F., Dale, C., and Dearing, M.D. (2016). Effect of Dietary Oxalate on the Gut Microbiota of the Mammalian Herbivore Neotoma albigula. Applied and environmental microbiology *82*, 2669-2675.
3. Belenguer, A., Bati, M.B., Hervás, G., Toral, P.G., Yáñez-Ruiz, D.R., and Frutos, P. (2013). Impact of oxalic acid on rumen function and bacterial community in sheep. animal *7*, 940-947.
4. Miller, A.W., Dale, C., and Dearing, M.D. (2017). Microbiota Diversification and Crash Induced by Dietary Oxalate in the Mammalian Herbivore Neotoma albigula. mSphere *2*. 10.1128/mSphere.00428-17.
5. Miller, A.W., Orr, T., Dearing, D., and Monga, M. (2019). Loss of function dysbiosis associated with antibiotics and high fat, high sugar diet. The ISME journal *13*, 1379-1390.
6. Hatch, M., Gjymishka, A., Salido, E.C., Allison, M.J., and Freel, R.W. (2011). Enteric oxalate elimination is induced and oxalate is normalized in a mouse model of primary hyperoxaluria following intestinal colonization with Oxalobacter. American Journal of Physiology-Gastrointestinal and Liver Physiology *300*, G461-G469.
7. Miller, A.W., Dale, C., and Dearing, M.D. (2017). The Induction of Oxalate Metabolism In Vivo Is More Effective with Functional Microbial Communities than with Functional Microbial Species. MSystems *2*, e00088-00017.
8. Miller, A.W., Oakeson, K.F., Dale, C., and Dearing, M.D. (2016). Microbial community transplant results in increased and long-term oxalate degradation. Microbial ecology *72*, 470-478.
9. Vijay-Kumar, M., Aitken, J.D., Carvalho, F.A., Cullender, T.C., Mwangi, S., Srinivasan, S., Sitaraman, S.V., Knight, R., Ley, R.E., and Gewirtz, A.T. (2010). Metabolic syndrome and altered gut microbiota in mice lacking Toll-like receptor 5. Science *328*, 228-231.
10. Batagello, C.A., Monga, M., and Miller, A.W. (2018). Calcium oxalate urolithiasis: a case of missing microbes? Journal of endourology *32*, 995-1005.
11. Le Naour, J., Montégut, L., Joseph, A., Garbin, K., Vacchelli, E., Kroemer, G., Pol, J.G., and Maiuri, M.C. (2022). Improved Swiss-rolling method for histological analyses of colon tissue. MethodsX *9*, 101630.
12. Koelink, P.J., Wildenberg, M.E., Stitt, L.W., Feagan, B.G., Koldijk, M., van ‘t Wout, A.B., Atreya, R., Vieth, M., Brandse, J.F., and Duijst, S. (2018). Development of reliable, valid and responsive scoring systems for endoscopy and histology in animal models for inflammatory bowel disease. Journal of Crohn's and Colitis *12*, 794-803.
13. Rungby, J., Kassem, M., Eriksen, E.F., and Danscher, G. (1993). The von Kossa reaction for calcium deposits: silver lactate staining increases sensitivity and reduces background. The Histochemical Journal *25*, 446-451.
14. Bankhead, P., Loughrey, M.B., Fernández, J.A., Dombrowski, Y., McArt, D.G., Dunne, P.D., McQuaid, S., Gray, R.T., Murray, L.J., and Coleman, H.G. (2017).

bioRxiv preprint doi: https://doi.org/10.1101/2024.10.28.620613; this version posted October 28, 2024. The copyright holder for this preprint (which was not certified by peer review) is the author/funder, who has granted bioRxiv a license to display the preprint in perpetuity. It is made available under aCC-BY 4.0 International license.

QuPath: Open source software for digital pathology image analysis. Scientific reports *7*, 1-7.

15. Miller, A.W., Kohl, K.D., and Dearing, M.D. (2014). The gastrointestinal tract of the white-throated woodrat (Neotoma albigula) harbors distinct consortia of oxalate-degrading bacteria. Applied and environmental microbiology *80*, 1595-1601.
16. Goodman, A.L., Kallstrom, G., Faith, J.J., Reyes, A., Moore, A., Dantas, G., and Gordon, J.I. (2011). Extensive personal human gut microbiota culture collections characterized and manipulated in gnotobiotic mice. Proceedings of the National Academy of Sciences *108*, 6252-6257.
17. Mak, T.D., Laiakis, E.C., Goudarzi, M., and Fornace Jr, A.J. (2013). Metabolyzer: A novel statistical workflow for analyzing postprocessed lc–ms metabolomics data. Analytical chemistry *86*, 506-513.
18. Okuda, S., Yamada, T., Hamajima, M., Itoh, M., Katayama, T., Bork, P., Goto, S., and Kanehisa, M. (2008). KEGG Atlas mapping for global analysis of metabolic pathways. Nucleic acids research *36*, W423-W426.
19. Wishart, D.S., Feunang, Y.D., Marcu, A., Guo, A.C., Liang, K., Vázquez-Fresno, R., Sajed, T., Johnson, D., Li, C., and Karu, N. (2017). HMDB 4.0: the human metabolome database for 2018. Nucleic acids research *46*, D608-D617.
20. O'Donnell, V.B., Dennis, E.A., Wakelam, M.J., and Subramaniam, S. (2019). LIPID MAPS: Serving the next generation of lipid researchers with tools, resources, data, and training. Science signaling *12*, eaaw2964.
21. Karp, P.D., Billington, R., Caspi, R., Fulcher, C.A., Latendresse, M., Kothari, A., Keseler, I.M., Krummenacker, M., Midford, P.E., and Ong, Q. (2019). The BioCyc collection of microbial genomes and metabolic pathways. Briefings in bioinformatics *20*, 1085-1093.
22. Qi, Z., and Voit, E.O. (2016). Strategies for comparing metabolic profiles: Implications for the inference of biochemical mechanisms from metabolomics data. IEEE/ACM transactions on computational biology and bioinformatics *14*, 1434-1445.
23. Oksanen, J., Blanchet, F.G., Kindt, R., Legendre, P., O'hara, R., Simpson, G.L., Solymos, P., Stevens, M.H.H., and Wagner, H. (2010). Vegan: community ecology package. R package version 1.17-4. http://cran. r-project. org>. Acesso em *23*, 2010.
24. Delhomme, N., Mähler, N., Schiffthaler, B., Sundell, D., Mannapperuma, C., Hvidsten, T.R., and Street, N.R. (2014). Guidelines for RNA-Seq data analysis. Epigenesys Protoc *67*, 1-24.
25. Kopylova, E., Noé, L., and Touzet, H. (2012). SortMeRNA: fast and accurate filtering of ribosomal RNAs in metatranscriptomic data. Bioinformatics *28*, 3211-3217.
26. Andrews, S., Krueger, F., Segonds-Pichon, A., Biggins, L., Krueger, C., and Wingett, S. (2010). FastQC. A quality control tool for high throughput sequence data *370*.
27. Bolger, A.M., Lohse, M., and Usadel, B. (2014). Trimmomatic: a flexible trimmer for Illumina sequence data. Bioinformatics *30*, 2114-2120.

bioRxiv preprint doi: https://doi.org/10.1101/2024.10.28.620613; this version posted October 28, 2024. The copyright holder for this preprint (which was not certified by peer review) is the author/funder, who has granted bioRxiv a license to display the preprint in perpetuity. It is made available under aCC-BY 4.0 International license.

28. Karolchik, D., Barber, G.P., Casper, J., Clawson, H., Cline, M.S., Diekhans, M., Dreszer, T.R., Fujita, P.A., Guruvadoo, L., and Haeussler, M. (2014). The UCSC genome browser database: 2014 update. Nucleic acids research *42*, D764-D770.
29. Pertea, M., Kim, D., Pertea, G.M., Leek, J.T., and Salzberg, S.L. (2016). Transcript-level expression analysis of RNA-seq experiments with HISAT, StringTie and Ballgown. Nature protocols *11*, 1650-1667.
30. Team, R.C. (2013). R: A language and environment for statistical computing.
31. Trapnell, C., Hendrickson, D.G., Sauvageau, M., Goff, L., Rinn, J.L., and Pachter, L. (2013). Differential analysis of gene regulation at transcript resolution with RNA-seq. Nature biotechnology *31*, 46-53.
32. Bult, C.J., Blake, J.A., Smith, C.L., Kadin, J.A., and Richardson, J.E. (2019). Mouse genome database (MGD) 2019. Nucleic acids research *47*, D801-D806.
33. Watts, S.C., Ritchie, S.C., Inouye, M., and Holt, K.E. (2019). FastSpar: rapid and scalable correlation estimation for compositional data. Bioinformatics *35*, 1064-1066.
34. Shannon, P., Markiel, A., Ozier, O., Baliga, N.S., Wang, J.T., Ramage, D., Amin, N., Schwikowski, B., and Ideker, T. (2003). Cytoscape: a software environment for integrated models of biomolecular interaction networks. Genome research *13*, 2498-2504.
35. Bushnell, B., Rood, J., and Singer, E. (2017). BBMerge–accurate paired shotgun read merging via overlap. PLoS One *12*, e0185056.
36. Campbell, M., Oakeson, K.F., Yandell, M., Halpert, J.R., and Dearing, D. (2016). The draft genome sequence and annotation of the desert woodrat Neotoma lepida. Genomics Data *9*, 58-59.
37. Li, H. (2013). Aligning sequence reads, clone sequences and assembly contigs with BWA-MEM. arXiv preprint arXiv:1303.3997.
38. Nurk, S., Meleshko, D., Korobeynikov, A., and Pevzner, P.A. (2017). metaSPAdes: a new versatile metagenomic assembler. Genome research *27*, 824-834.
39. Seemann, T. (2014). Prokka: rapid prokaryotic genome annotation. Bioinformatics *30*, 2068-2069.
40. Consortium, U. (2015). UniProt: a hub for protein information. Nucleic acids research *43*, D204-D212.
41. Pedruzzi, I., Rivoire, C., Auchincloss, A.H., Coudert, E., Keller, G., De Castro, E., Baratin, D., Cuche, B.A., Bougueleret, L., and Poux, S. (2015). HAMAP in 2015: updates to the protein family classification and annotation system. Nucleic acids research *43*, D1064-D1070.
42. Fu, L., Niu, B., Zhu, Z., Wu, S., and Li, W. (2012). CD-HIT: accelerated for clustering the next-generation sequencing data. Bioinformatics *28*, 3150-3152.
43. Love, M.I., Huber, W., and Anders, S. (2014). Moderated estimation of fold change and dispersion for RNA-seq data with DESeq2. Genome biology *15*, 1.
44. Miller, I.J., Rees, E.R., Ross, J., Miller, I., Baxa, J., Lopera, J., Kerby, R.L., Rey, F.E., and Kwan, J.C. (2019). Autometa: automated extraction of microbial genomes from individual shotgun metagenomes. Nucleic acids research *47*, e57-e57.

bioRxiv preprint doi: https://doi.org/10.1101/2024.10.28.620613; this version posted October 28, 2024. The copyright holder for this preprint (which was not certified by peer review) is the author/funder, who has granted bioRxiv a license to display the preprint in perpetuity. It is made available under aCC-BY 4.0 International license.

45. Kachroo, N., Monga, M., and Miller, A.W. (2022). Comparative functional analysis of the urinary tract microbiome for individuals with or without calcium oxalate calculi. Urolithiasis *50*, 303-317. 10.1007/s00240-022-01314-5.
46. Chen, K.-T., Liu, C.-L., Huang, S.-H., Shen, H.-T., Shieh, Y.-K., Chiu, H.-T., and Lu, C.L. (2018). CSAR: a contig scaffolding tool using algebraic rearrangements. Bioinformatics *34*, 109-111.
47. Simpson, J.T., Wong, K., Jackman, S.D., Schein, J.E., Jones, S.J., and Birol, I. (2009). ABySS: a parallel assembler for short read sequence data. Genome research *19*, 1117-1123.
48. Asnicar, F., Thomas, A.M., Beghini, F., Mengoni, C., Manara, S., Manghi, P., Zhu, Q., Bolzan, M., Cumbo, F., and May, U. (2020). Precise phylogenetic analysis of microbial isolates and genomes from metagenomes using PhyloPhlAn 3.0. Nature communications *11*, 1-10.
49. Bowers, R.M., Kyrpides, N.C., Stepanauskas, R., Harmon-Smith, M., Doud, D., Reddy, T., Schulz, F., Jarett, J., Rivers, A.R., and Eloe-Fadrosh, E.A. (2017). Minimum information about a single amplified genome (MISAG) and a metagenome-assembled genome (MIMAG) of bacteria and archaea. Nature biotechnology *35*, 725-731.
50. Kanehisa, M., Sato, Y., Furumichi, M., Morishima, K., and Tanabe, M. (2018). New approach for understanding genome variations in KEGG. Nucleic acids research *47*, D590-D595.
51. Callahan, B.J., McMurdie, P.J., Rosen, M.J., Han, A.W., Johnson, A.J.A., and Holmes, S.P. (2016). DADA2: high-resolution sample inference from Illumina amplicon data. Nature methods *13*, 581.
52. Balvočiūtė, M., and Huson, D.H. (2017). SILVA, RDP, Greengenes, NCBI and OTT—how do these taxonomies compare? BMC genomics *18*, 114.
53. Bodenhofer, U., Bonatesta, E., Horejš-Kainrath, C., and Hochreiter, S. (2015). msa: an R package for multiple sequence alignment. Bioinformatics *31*, 3997-3999.
54. Schliep, K.P. (2011). phangorn: phylogenetic analysis in R. Bioinformatics *27*, 592-593.
55. McMurdie, P.J., and Holmes, S. (2013). phyloseq: an R package for reproducible interactive analysis and graphics of microbiome census data. PloS one *8*, e61217.
56. Lozupone, C., Hamady, M., and Knight, R. (2006). UniFrac–an online tool for comparing microbial community diversity in a phylogenetic context. BMC bioinformatics *7*, 1.
